# Compressive Big Data Analytics: An ensemble meta-algorithm for high-dimensional multisource datasets

**DOI:** 10.1371/journal.pone.0228520

**Published:** 2020-08-28

**Authors:** Simeone Marino, Yi Zhao, Nina Zhou, Yiwang Zhou, Arthur W. Toga, Lu Zhao, Yingsi Jian, Yichen Yang, Yehu Chen, Qiucheng Wu, Jessica Wild, Brandon Cummings, Ivo D. Dinov

**Affiliations:** 1 Statistics Online Computational Resource, Department of Health Behavior and Biological Sciences, University of Michigan, Ann Arbor, Michigan, United States of America; 2 Department of Microbiology and Immunology, University of Michigan, Ann Arbor, Michigan, United States of America; 3 Department of Computational Medicine and Bioinformatics, University of Michigan, Ann Arbor, Michigan, United States of America; 4 Department of Biostatistics, University of Michigan, Ann Arbor, Michigan, United States of America; 5 Laboratory of Neuro Imaging, USC Stevens Neuroimaging and Informatics Institute, Keck School of Medicine of USC, University of Southern California, Los Angeles, California, United States of America; 6 Michigan Center for Integrative Research in Critical Care, University of Michigan, Ann Arbor, Michigan, United States of America; 7 Michigan Institute for Data Science, University of Michigan, Ann Arbor, Michigan, United States of America; 8 Neuroscience Graduate Program, University of Michigan, Ann Arbor, Michigan, United States of America; Clemson University, UNITED STATES

## Abstract

Health advances are contingent on continuous development of new methods and approaches to foster data-driven discovery in the biomedical and clinical sciences. Open-science and team-based scientific discovery offer hope for tackling some of the difficult challenges associated with managing, modeling, and interpreting of large, complex, and multisource data. Translating raw observations into useful information and actionable knowledge depends on effective domain-independent reproducibility, area-specific replicability, data curation, analysis protocols, organization, management and sharing of health-related digital objects. This study expands the functionality and utility of an ensemble semi-supervised machine learning technique called Compressive Big Data Analytics (CBDA). Applied to high-dimensional data, CBDA (1) identifies salient features and key biomarkers enabling reliable and reproducible forecasting of binary, multinomial and continuous outcomes (i.e., feature mining); and (2) suggests the most accurate algorithms/models for predictive analytics of the observed data (i.e., model mining). The method relies on iterative subsampling, combines function optimization and statistical inference, and generates ensemble predictions for observed univariate outcomes. The novelty of this study is highlighted by a new and expanded set of CBDA features including (1) efficiently handling extremely large datasets (>100,000 cases and >1,000 features); (2) generalizing the internal and external validation steps; (3) expanding the set of base-learners for joint ensemble prediction; (4) introducing an automated selection of CBDA specifications; and (5) providing mechanisms to assess CBDA convergence, evaluate the prediction accuracy, and measure result consistency. To ground the mathematical model and the corresponding computational algorithm, CBDA 2.0 validation utilizes synthetic datasets as well as a population-wide census-like study. Specifically, an empirical validation of the CBDA technique is based on a translational health research using a large-scale clinical study (UK Biobank), which includes imaging, cognitive, and clinical assessment data. The UK Biobank archive presents several difficult challenges related to the aggregation, harmonization, modeling, and interrogation of the information. These problems are related to the complex longitudinal structure, variable heterogeneity, feature multicollinearity, incongruency, and missingness, as well as violations of classical parametric assumptions. Our results show the scalability, efficiency, and usability of CBDA to interrogate complex data into structural information leading to derived knowledge and translational action. Applying CBDA 2.0 to the UK Biobank case-study allows predicting various outcomes of interest, e.g., mood disorders and irritability, and suggests new and exciting avenues of evidence-based research in the context of identifying, tracking, and treating mental health and aging-related diseases. Following open-science principles, we share the entire end-to-end protocol, source-code, and results. This facilitates independent validation, result reproducibility, and team-based collaborative discovery.

## 1. Introduction

Data Science is an emerging transdisciplinary field connecting the theoretical, computational, experimental, biomedical, social, environmental and economic areas. It deals with enormous amounts of complex, incongruent, and dynamic data (Big Data) from multiple sources and aims to develop algorithms, methods, tools, and services capable of ingesting such datasets and generating semi-automated decision support systems. The lack of a comprehensive or canonical mathematical formulation of Data Science is one of the major challenges in the development of its theoretical foundations. Other significant hurdles and gaps pertain to the nature of Big Data and the tools and methods to handle them. Examples of the former are Big Data heterogeneity [1], noise concentration [2], spurious correlations [3], among others. Advanced tools and ensemble methods to handle large, time-varying, and heterogeneous datasets rely on robust predictive models, the specification and implementation of optimal, feasible, scalable, and convergent algorithms, advanced computational workflow protocols, access to appropriate computational resources, and scalable infrastructure.

Previously, we proposed a scalable framework for Big Data representation, high-throughput analytics (variable selection, predictive modeling, and noise reduction), and model-free inference that we called *Compressive Big Data Analytics (CBDA)* [4]. We showed the robustness, efficiency, accuracy and viability of the first generation CBDA method for training predictive models and for feature selection, and validated it on small-to-medium sized data. In this manuscript, we expand the CBDA method and test the new CBDA 2.0 technique on large synthetic datasets (e.g., ranging from 10,000–1,000,000 cases and 1,000–10,000 features). In addition, we validate CBDA 2.0 by applying it for detection and prediction of mood disorders (e.g., irritability) using a large population-based clinical survey, the UK Biobank [5, 6] (see *Datasets* section for details).

The CBDA protocol relies on model-based statistical computing methods and model-free data analytics [7]. Each such method provides efficient parameter estimations, reliable predictions, and robust scientific inference based on imaging, phenotypic, genetics and clinical data. The two main strategies used by CBDA to explore the core principles of distribution-free and model-agnostic methods for scientific inference based on complex datasets are *subsampling*, or bootstrapping, and *ensemble prediction*. Ensemble prediction and subsampling/bootstrapping algorithms use common approaches for objective function optimization, quantification of noise, bias estimation, prediction error estimation, and variance estimation during the learning/training processes.

Standard ensemble methods, such as bagging and boosting [8–10], usually aggregate the results of a *single* “base” learner algorithm, e.g., support vector machine (SVM) [11] or k-nearest neighbor (kNN) [12]. CBDA employs SuperLearner [13, 14] as its ensemble predictor to combine *multiple* "base" learner algorithms into a blend of meta-learners. In addition, CBDA utilizes ensemble methods in two stages, during the *training* step as well as during the subsequent overfitting *testing* step (see S1 Fig in S1 File for details).

Although advanced ensemble methods like Random Forest [15, 16] could change the features’ weights during iterations, they do not directly reveal the importance of each individual feature. CBDA explicates the feature importance at each experimental iteration. Similar to signal estimation in compressive sensing [17], CBDA reduces the problem dimension and efficiently derives reliable and reproducible inference. In the iterative process of computing the final inference, CBDA subsampling selects stochastically both features and cases, much like compressive sensing randomly traverses the state space. CBDA identifies an optimal feature-space, which may not necessarily be an average of the intermediate results.

Since its CRAN publication in 2018 [4], the CBDA package had an average of 328 downloads per months over the past 2 years. The first version of the CBDA method was implemented as a stand-alone R package [4], which can be deployed on any desktop, laptop, or HPC cluster environment. For example, we demonstrated deploying CBDA 1.0 on a high-performance computing platform using the LONI graphical pipeline environment [18]. In this manuscript, we are enhancing the CBDA method, expanding its applications, and testing it on large and very heterogeneous datasets. These improvements are reflected in an integrated and upgraded CBDA 2.0 R package that is also tested on the LONI Pipeline workflow environment. In the Pipeline environment, the entire CBDA 2.0 protocol is implemented via pipeline module wrappers and includes various pre-processing and post-processing steps natively representing bash/shell, R, and Perl scripts that optimize the iterative CBDA subsampling phases. The CBDA 2.0 software release documentation and detailed description of features, improvements, and limitations are available on the CBDA GitHub repository [19].

The upgraded CBDA protocol further expands on the set of machine learning algorithms embedded in the ensemble predictor (i.e., SuperLearner [13, 14]). This new set allows the testing of model performance and overall convergence metrics, which will inform the validation step and help transitioning the predictive analytics into the estimation/inference phase. The analysis of the ensemble predictor weights across the many subsamples and machine learning algorithms can also suggest a way to empirically check the CBDA computational convergence.

As a last contribution, we recast our initial mathematical formulation to improve the study of the ergodic properties and the asymptotics of the specific statistical inference approaches utilized within the CBDA technique. This new simplified and more compact formulation is presented in the S1 Text in S1 File.

Our results suggest that the CBDA methodology is scalable and accurate. One of the strengths of combining a stochastic subsampling strategy with ensemble prediction is the ability of sifting through data where no signal is present, with low false discovery rates. The application to a real case study, like the UK Biobank, highlights the CBDA flexibility and scalability in complex predictive analytics scenarios with incongruent, heterogeneous and highly-correlated data, as well as with data with substantial proportion of missingness (greater than 50%).

## 2. Materials and methods

**Fig 1:**
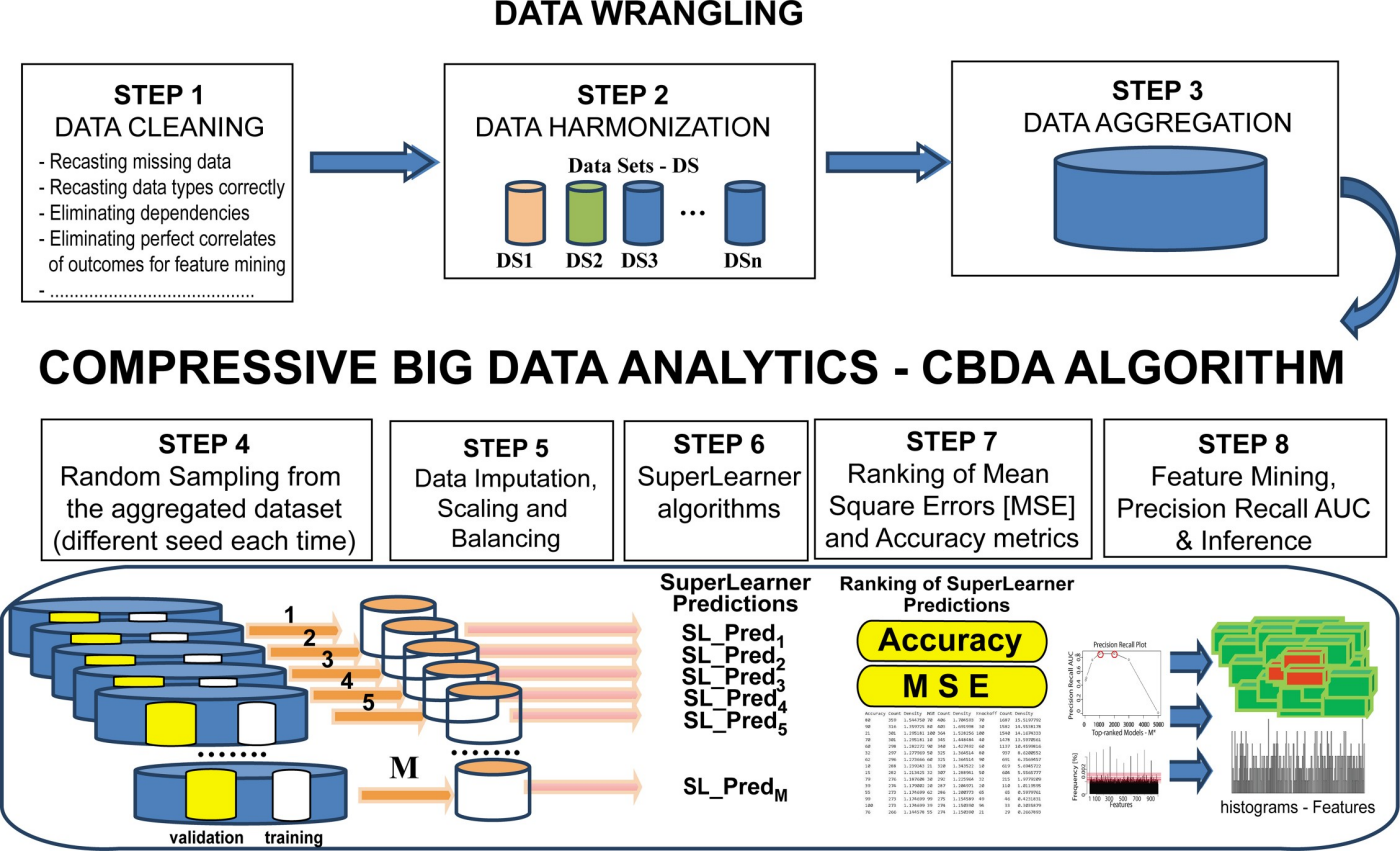
Schematic of the improved CBDA 2.0 workflow.

This section illustrates the new CBDA protocol for representing and analyzing large datasets with binomial, multinomial, and continuous outcomes. First, we briefly review the main steps of the method and then describe in detail the new steps added to the workflow. We then review the validation strategy and examine the results of using synthetic and clinical datasets. The end-to-end CBDA processing workflow is shown in Fig 1.

The entire CBDA protocol is designed, implemented and validated as a reproducible, open-project using the statistical computing language R [4, 20]. A number of training sets have been used to assess the convergence of the CBDA technique through a workflow protocol described in [4, 19]. This validation workflow runs on the LONI pipeline environment [18], a free platform for high performance computing, which allows the simultaneous submission of hundreds of independent components of the CBDA protocol (see [21] for details).

The first generation CBDA methodology and its implementation [4] can handle predictive analytics for datasets up to 1GB. Depending on the available hardware infrastructure and the user-controlled parameters, the enhanced next-generation CBDA 2.0 can handle gigabytes of data for millions of cases. Our new implementation combines shell/bash and Perl scripts to efficiently perform data preprocessing during the subsampling steps, data staging, data post-processing, validation, and predictive analytics. The following sections outline the basics of the CBDA methodology, drawing parallels between CBDA and alternative ensemble predictor and bootstrapping strategies. Later, we will describe in detail the CBDA 2.0 implementation and highlight the new upgrades and improvements. The entire CBDA protocol is shown as pseudocode on S1 Fig in S1 File.

### 2.1 A new CBDA subsampling strategy

The initial CBDA subsampling strategy is fully described in [4]. Briefly, both cases and features in the original Big Data are sampled with certain specifications given by the *Case Sampling Range* (CSR) and *Feature Sampling Range* (FSR). Our new implementation has two major subsampling advantages. The main objective of subsampling is to pass a representative, balanced (if possible), and moderately-small sample of the complete dataset to the ensemble predictor for faster analysis. In order to generalize and automate the subsampling strategy, the first novelty of CBDA 2.0 is to set an upper bound in terms of number of cases and features for the subsampled datasets, namely 300 cases and 30 features. Instead of sampling a proportion of the original dataset (*CSR*×*cases*, *FSR*×*features*), which increases with the increase of the data complexity, the new sampling scheme is data-size independent. Since the sample of features is small enough, it largely avoids the joint presence of highly correlated variables and enables CBDA to deal with potential multicollinearities in the data set. Other ratio cases/features can be further explored augmenting our previous report [4]. More investigation is needed to determine theoretical optimal cases/features ratio values. The goal of this study is to set a reasonably small subsample size (relative to the size of the original dataset) that is sufficient to support predictive analytics and at the same time enable effective use of system resources (memory and computational cycles).

Table 1 shows how the subsample size (e.g., 300x30), combined with the Big Data sizes, can be recasted into the previously used ranges for CSR and FSR. The new subsampling protocol significantly improves on the compression of the data needed to reconstruct the original signal (at least in the synthetic case studies) by *O*(log *n*) in the case of 100,000–1,000,000 million cases (see Table 1 for details). We also reduced the total number of subsamples *M* = 5,000 (comparing to *M* = 9,000 used in our previous study [4]). We tested CBDA 2.0 with synthetic datasets up to 70GB, but the protocol can easily be applied to larger datasets since we operate with pre-defined subsamples size (i.e., 300x30) and total number of subsamples *M* (e.g., 5,000).

**Table 1:** Subsampling specifications for different Big Data sizes. The total number of subsamples M = 5,000.

Description	n (cases)	p (features)	Sampling Rates	
			CSR (cases)	FSR (features)
*Medium Synthetic Datasets*	10,000	1,000	3%	3%
*Large Synthetic Datasets*	100,000	10,000	0.3%	0.3%
*Very Large Synthetic Datasets*	1,000,000	10,000	0.03%	0.3%

There are natural barriers in dealing with large and heterogeneous datasets. Alternatives subsampling approaches can be implemented to facilitate faster access and manipulation of extremely large datasets, including Spark, Scala, Julia, Python, and more (see [22] for comparisons and details). The current CBDA workflow and R code are available and constantly updated on the CBDA GitHub repository [19]. To optimize the computationally intensive subsampling step, we are also actively working on an efficient Python implementation. Please refer to our GitHub repository [19] for code updates.

Upon initializing the subsampling, the second novelty of the CBDA 2.0 protocol is a different way to perform the sampling of the validation set. In [4] we set aside 20% of the original Big Data for validation and never used it for training. However, the 20:80 split is not scalable for Big Data, since the validation set might be too large. Our new strategy samples a validation set each time we generate a subsample for training. Each validation set is twice the number of cases of the training set, with no case overlapping the training sample (see Section 2.3 for details). In this study, we use validation sets of 600 cases. Each sampling is done with replacement. As shown in Table 1, the high compression rates, achieved by fixing the maximum size of each subsamples, masks the potential overfitting risks. We recognize that over *M* subsamples, some records might be used for both training and internal validation. However, this is only occurring in some of the *M* predictive analytics steps, and never within the same training/validation sets. Given the low CSR and FSR, the likelihood to sample the final top-ranked features in the same subsample (as resulting after the CBDA Overfitting Test stage) is relatively small.

To account for possible multi-collinearity among multiple features, we define a scaling factor that augments the FSR: Variance Inflation Factor (VIF), similar to the ANOVA analysis [23].

If the subsampling is deployed on the same server where the original data is stored, the protocol performs extremely fast. The conditions are that the server has (1) a workflow system in place for the submission of multiple simultaneous jobs (e.g., LONI pipeline workflow, but it can be PBS (Portable Batch System) [24], or SLURM (Simple Linux Utility for Resource Management) [25], or other schedulers); (2) shell and Perl scripting is enabled; and (3) access to an R computing environment. The current CBDA 2.0 implementation is based on LONI pipeline server/client 7.0.3 and R 3.3.3.

If any of the conditions above are not fulfilled, either the data must be deployed on a server where the CBDA protocol can be executed, or, as a viable alternative, the subsampling can be done on the server hosting the data and only the subsamples need to be deployed on the server where the CBDA can be executed. The latter will offer a scalable solution, since the size of the total set of subsamples is significantly smaller than the entire dataset and can be reasonably predicted (e.g., approximately 1-2GBs).

### 2.2 CBDA ensemble prediction via the SuperLearner

The SuperLearner [13] and Targeted Maximum Likelihood Estimation (TMLE) [26, 27] theories have been developed in the past 10 years. Both are complimentary methods for parameter estimation in nonparametric statistical models for general data structures. The SuperLearner theory guides the construction of asymptotically optimal estimators of non-pathwise-differentiable parameters, e.g., prediction or density estimation. The TMLE theory guides the construction of efficient estimators of finite dimensional pathwise-differentiable parameters, e.g., marginal means. The CBDA protocol uses SuperLearner as a black-box machine learning ensemble predictor.

The SuperLearner technique could be considered as a data-adaptive machine learning approach to prediction and density estimation. It uses cross-validation to estimate the performance of multiple machine learning models, or the same model with different settings. The results shown in this study have been generated using 55 different classification and regression machine learning algorithms (see Table 2).

**Table 2:** Library of the 55 different classification and regression machine-learning algorithms used by the ensemble predictor SuperLearner (SL.library) in the CBDA 2.0 implementation.

Base Learner Class	Base Learner Different specifications	Reference
Glmnet **(SL.glmnet)**	"SL.glmnet" "SL.glmnet.0" "SL.glmnet.0.25" "SL.glmnet.0.50”"SL.glmnet.0.75“	[28, 29]
bartMachine **(SL.bartMachine)**	"SL.bartMachine" "SL.bartMachine.500" "SL.bartMachine.100”"SL.bartMachine.20“	[30, 31]
Support Vector Machine **(SL.svm)**	"SL.svm" "SL.svm.radial.10" "SL.svm.radial.0.1”"SL.svm.radial.default" "SL.svm.poly.2.0" "SL.svm.poly.3.0" "SL.svm.poly.3.10" "SL.svm.linear" "SL.svm.poly.3.n10" "SL.svm.poly.6.0" "SL.svm.sigmoid”"SL.svm.poly.6.10" "SL.svm.poly.6.n10"	[11]
Random Forest **(SL.randomForest)**	"SL.randomForest" "SL.randomForest.1000" "SL.randomForest.500" "SL.randomForest.300" "SL.randomForest.100" "SL.randomForest.50" "SL.randomForest.20“	[15, 16]
Xgboost **(SL. Xgboost)**	"SL.xgboost" "SL.xgboost.500" "SL.xgboost.300”"SL.xgboost.2000" "SL.xgboost.1500" "SL.xgboost.d3" "SL.xgboost.d5" "SL.xgboost.d6" "SL.xgboost.gau" "SL.xgboost.shrink.15" "SL.xgboost.shrink.2" "SL.xgboost.shrink.05" "SL.xgboost.shrink.25“	[9, 10, 32]
K-nearest neighbor **(SL.knn)**	"SL.knn","SL.knn.100","SL.knn.50","SL.knn.25", "SL.knn.5"	[12]
Others	"SL.glm" "SL.bayesglm" "SL.earth" "SL.glm.interaction”"SL.ipredbagg" "SL.mean" "SL.nnet" "SL.nnls“	

Details on default and modified parameters for each of the machine learning algorithm classes can be found in the S2 Text in S1 File. After each machine learning model has completed the analysis on each subsample, the SuperLearner creates an optimal weighted average of those models (i.e., ensemble predictor), using the test data performance. This approach has been proven to be asymptotically as accurate as the best possible prediction algorithm that is tested [13]. Although we do not directly discuss convergence results of the SuperLearner in this study, we outline a two-pronged approach for assessing the overall performance and computational convergence of our CBDA method (see S1 File).

### 2.3 CBDA two-phase bootstrapping strategy

CBDA resembles various ensemble methods, like bagging and boosting algorithms, in its use of the core principle of stochastic sampling to enhance the model prediction [33]. The purpose and utilization of the derived samples is what makes CBDA unique as it implements a *two-phase bootstrapping strategy*. In *phase one*, similar to the compressive sensing approach for signal reconstruction [34], CBDA bootstrapping is initiated by the divide-and-conquer strategy, where the Big Data is sampled with replacement following some input specifications (e.g., see section 2.1 and Table 1 here and as described in [35]). In *phase two*, during the CBDA-SuperLearner calculations, additional cross-validation and bootstrapping inputs are passed to each base learner included in the meta-learner/SuperLearner. In fact, the SuperLearner uses an internal 10-fold cross-validation, which is applied to each of the base learners available in the SL.library. Moreover, one of the (optional) inputs for the SuperLearner is a set of data for external validation. In this study, the external validation set is specified as twice the number of the cases of the training chunks (600 vs. 300) and the same number of features. Technically, this approach does not represent an external validation, since it comes from the same dataset, but no case included in the internal validation sample (600x30) would also appear in the training sample (300x30). We choose a much larger external validation set simply because there is no scarcity of data and also because the increased size does not affect CPU time (it’s just the application of the ensemble predictor model trained on the 300x30 sample).

As many distinct boosting methods can be included within the meta-learner library (e.g., XGBoost), combining the power of multi-classifier boosting within a single base learner into the larger CBDA ensemble prediction enhances the method’s power by aggregating across multiple base learners. Many studies examine the asymptotic convergence of bagging, boosting and ensemble methods [36–39]. Similar approaches may be employed to validate CBDA inference in terms of upper error bounds, convergence, and reliability. We highlight the strategies we pursue in this study in S3 Text in S1 File.

### 2.4 Signal filtering and False Discovery Rate (FDR) calculation

Since CBDA exploits the subsampling strategy for feature mining purposes, it is important to have an assessment of the False Discovery Rates (FDR) when ranking the most informative and predictive features. We describe now a new and general procedure to filter the signal and approximate CBDA False Discovery Rates. After the CBDA learning/training stage is completed, it is critical to determine the optimal number M* of top-ranked predictive models to choose from the total number *M* computed. We now outline a new procedure, which comprises two steps. The first step selects M* top-ranked models and plots the feature frequencies emerging from the M* subsamples. Then as a second step, we define a probabilistic cut-off *α* to select the likely signal (i.e., a subset of features with significantly higher frequencies). In this context, a signal is a feature frequency significantly higher than the others.

**Fig 2:**
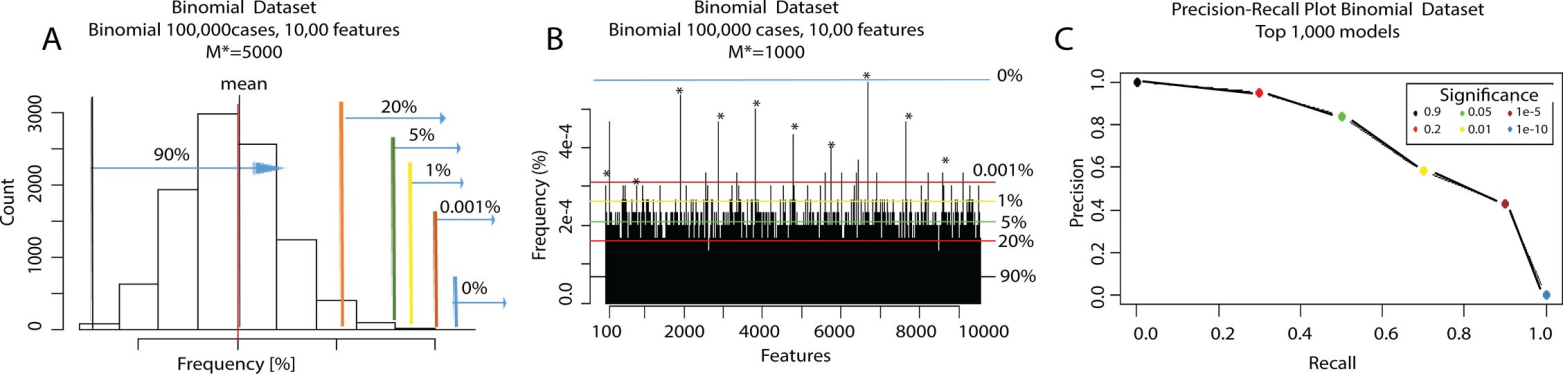
Procedure to generate Precision Recall and AUC plots. The consistent colors in the three Panels indicate identical significance levels based on the “normal” histogram in Panel A. Panel A: histogram of feature frequencies for the Binomial dataset with 100,000 cases and 10,000 features, for M* = 5,000. Panel B: histogram of the features frequencies across the 1,000 Top Ranked models. Panel C: Precision-Recall plot for the Binomial dataset with 100,000 cases and 10,000 features, for M* = 1,000. Each circle represents a cutoff based on Fig 2B distribution for different *α* (shown in the legend of Panel C). The area under the curve (AUC) in Panel C is then used to assess the quality and accuracy of M* (i.e., Top-Ranked models to consider).

Now, M* can be between 0 and *M*. If M* is too low or too high, no signal can be detected (see Fig 2A–2C). For each M* value chosen, we plot the resulting feature frequency distribution. For M* = *M*, each feature frequency follows a binomial distribution, with probability $\frac{1}{p}$. The distribution of the feature frequencies then follows a normal distribution, since it is the result of a linear transformation of a very large number of binomial stochastic variables (see [40] for details). The mean *μ* and standard deviation *σ* of the features frequency distribution across the *M* subsamples can give us a way to control the CBDA False Discovery Rate and suggests a cut-off for selecting True Positives (i.e., TP) for different M*. By construction, if we plot the distribution of the feature frequencies across the *M* subsamples, we see an approximately normal distribution centered on the mean frequency $\mu ∼\frac{1}{p}$ with a certain variability given by the standard deviation *σ* (see Fig 2A). By setting different *α* we can then control false positives (i.e., FP).

**Fig 3:**
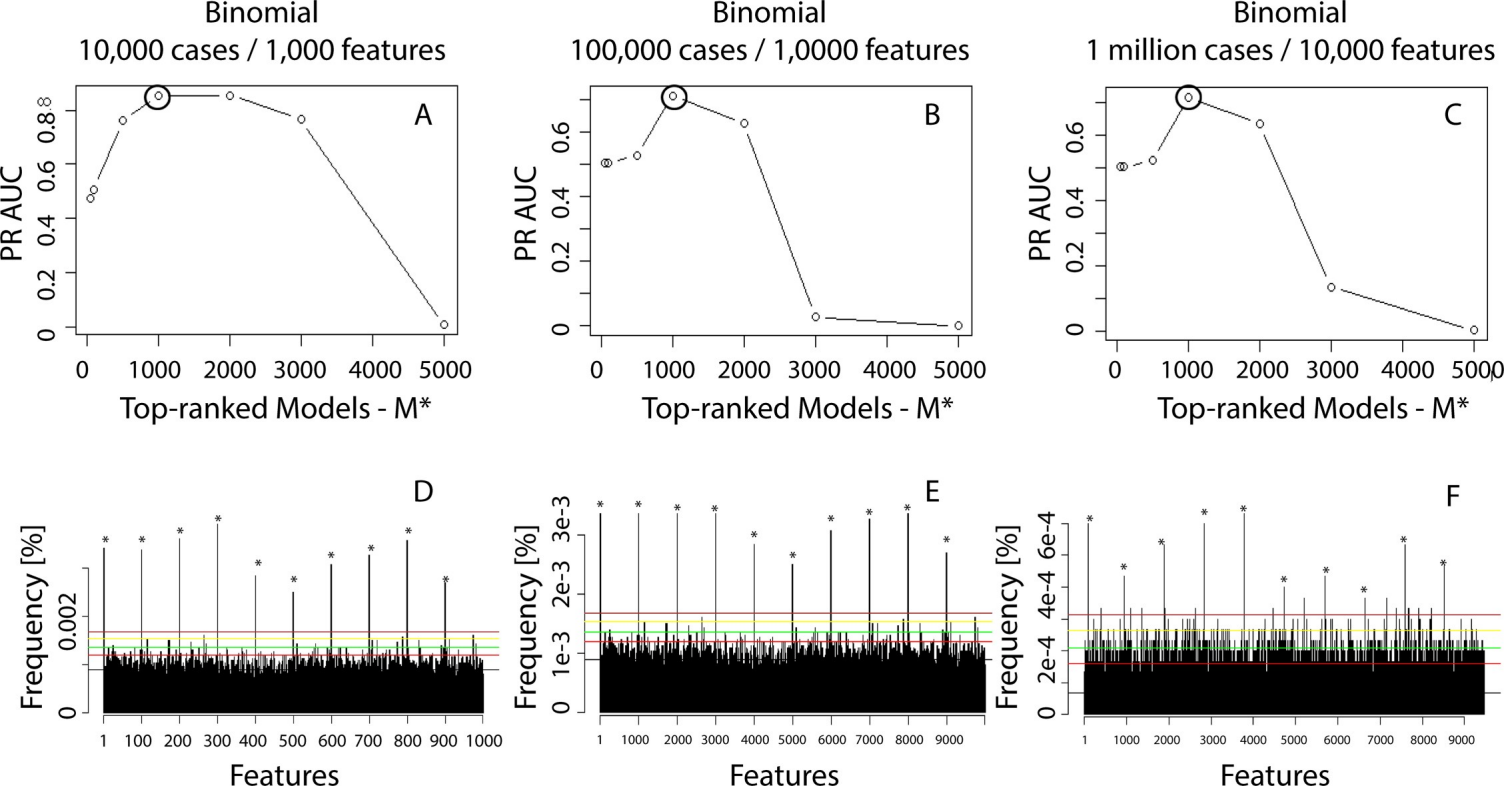
Precision Recall AUC values for different synthetic datasets and top-ranked models M*. We always performed *M* = 5,000 subsamples. We calculated the PR AUV values in Panels A-C for different values of M*, e.g., 100, 500, 1,000, 2,000, 3,000 and 5,000. The horizontal lines in Panels D-F are calculated for *α* values 0.9 (black), 1e-2 (red), 1e-5 (green), 1e-10 (yellow), 1e-16 (brown) and 1e-20 (blue). Fig 2 has more details on how the horizontal line values are calculated.

For example with an *α* = *e*^−6^, we obtain *CBDA*_*FDR−cutoff*_ = *μ*+4.75*σ*. Decreasing *α* forces a more conservative FDR. The criteria will consider any feature density value above the *CBDA*_*FDR−cutoff*_ as a Positive. For the synthetic datasets, the new CBDA function *CBDA_slicer()* will generate AUC (i.e., Area Under the Curve) trajectories based on different M* and *α*. The maximum of each trajectory will determine the optimal M* (see Fig 3A–3C). The False Discovery Rate can be then calculated as the ratio of False Positives over the Positives (i.e., FP/P).

Fig 2A shows an example of the histogram of feature frequencies resulting from one of the synthetic dataset results for M* = 5000 (Fig 2A). Fig 2 is meant to be illustrative of the methodology rather than showing the specific results. Based on the features distribution shown in Fig 2B and the hypothetical cut-off values set in Fig 2A, several *α* determine the values for the Precision-Recall (PR) plots, see Fig 2C, which shows the PR AUC plot for M* = 1,000.

In real case scenarios, the True Positive rate may not be known. Thus, the feature mining process may be guided by the optimal settings suggested by the synthetic case studies. Namely, we used the best top-ranked value (i.e., 1,000) and 5,000, 300x30 and 600x30 for the total number of samples, size of the training and validation sets, respectively.

#### 2.4.1. Precision-Recall and AUC trajectories

Usually, the number of features measured in Big Data is very large (e.g., ≤10K), resulting in a number of True Negatives (TN, referring to features) that is typically 2–3 logs larger than the number of True Positives (TP). Due to this built-in class imbalance, the classical ROC curves (i.e., False Positive Rates-PPR vs. True Positive Rates-TPR) are not very informative in discriminating between different M* combinations. We then decide to use Precision-Recall (PR) plots to select the best M* and *α*, since they do not account for True Negatives (TN). The definitions of Precision and Recall are given below:

$$Recall=\frac{TP}{TP+FP}\;and\;Precision=\frac{TP}{TP+FN}.$$

Where FP and FN represent False Positives and False Negatives, respectively. The Precision Recall Area Under Curve (PR AUC) is defined as the area under the PR curve, and that is what we use to generate our AUC trajectories. An ideal algorithm with only true positives and negatives would have a PR curve in the upper-right corner and a PR AUC of one. The new function called *CBDA_slicer()* generates a set of plots after the training stage of CBDA that display (1) the frequency of each feature as a function of the top-ranked M* models, (2) the correspondent PR plot, and (3) an AUC plot. The AUC plot summarizes the results of the RP as a function of M*. In a real case scenario, the function *CBDA_slicer()* generates a final False Discovery Rate plot, instead of the AUC plot, since no true positives are known. Details on the *CBDA_slicer()* function can be found in the S4 Text in S1 File as well as in through the *help()* method part of CBDA 2.0 package [19].

A new function called *Overfitting_plot()* generates two plots for each dataset after the Overfitting Test stage of the CBDA is completed (see Overfitting Plots in S1 Fig in S1 File). The x axis of each plot shows the 100 top-ranked features resulted from the CBDA training stage. The y axis shows Accuracy and MSE, respectively. By analyzing the trend of the overfitting plots, we can have a general assessment of the overall performance of the CBDA protocol on any dataset. This should be the first function to call at the end of the CBDA Overfitting Test stage. Details on the *Overfitting_plot()* function can be found in the S4 Text in S1 File and the *help()* function in the CBDA package [19].

### 2.5 Datasets

For the purpose of testing the protocol and assessing the CBDA performance. We validate the CBDA technique on three different datasets. The first two, namely the Null and Binomial datasets, are synthetically generated as cases (*n*) and features (*p*), see Table 1 for details. We used synthetic dataset to test the CBDA feature mining capability, controlling for true and false positives. Similar to our previous study, for all the Binomial datasets, only 10 features are used to generate the binary outcome variable (these are what we call truly predictive features, see details below in the Binomial Datasets section). The real case-study represents a real biomedical dataset on aging and neurodegenerative disorders (UK Biobank) [5, 6].

#### 2.5.1. Null datasets

The first set of data is a "white noise" dataset (i.e., Null dataset), where the outcome *Y* is a realization of a Bernoulli vector of length *n* (i.e., *Y* = [*Y*_1_,*Y*_2_,…,*Y*_*n*_], with *Y*_*i*_~*Bernoulli*(0.5), *i* = 1,2,….,*n*) that is completely independent from the set of features *X*. Each column of *X* is an independent realization of a Gaussian random variable with mean 0 and standard deviation 1 (i.e., *X* = [*X*_1_,*X*_2_,…,*X*_*p*_], with *X*_*j*_~*N*(0,1),*j* = 1,2,…,*p*. We will refer to *n* as number of cases and to *p* as number of features.

#### 2.5.2. Binomial datasets

The second set of data is similar to the Null dataset, but the Bernoulli vector *Y* is now an explicit function of the set of features. We establish the dependency of *Y* to *X* by selecting 10 features from *X* to build a linear additive model *Y*~*X*, with non-zero coefficients for only these 10 features:

$$Z=b_{k_{1}}X_{k_{1}}+b_{k_{2}}X_{k_{2}}+b_{k_{3}}X_{k_{3}}+\cdots +b_{k_{10}}X_{k_{10}}+e,\;with\;e∼N(0,1)\;and$$

$$b=b_{k_{j}}(j=1,2,\ldots ,10))$$

The Bernoulli outcome Y is then generated by an inverse logit on the outcome of the linear additive model (i.e., $Pr=\frac{e^{z}}{1+e^{Z}}$ and *Y*_*i*_~*Bernoulli*(*Pr*),*i* = 1,2,…,*n*). When necessary, various strategies may be used to binarize the predicted outcomes using the corresponding probability values.

#### 2.5.3. UK Biobank archive

The UK Biobank dataset is a rich national health resource that provides census-like multisource healthcare data. The archive presents the perfect case study because of its several challenges related to aggregation and harmonization of complex data elements, feature heterogeneity, incongruency and missingness, as well as health analytics. We built on our previous UK Biobank explorative study [6] and expand to include several outcomes for classification and prediction using our CBDA protocol.

The UK Biobank dataset comprises 502,627 subjects with 4,317 features ([41] and www.ukbiobak.ac.uk). A smaller UK Biobank subset with 9,914 subjects has a complete set of neuroimaging biomarkers. By matching the ID field, we are able to merge the two datasets into a more comprehensive one with 9,914 subjects and 7,614 features (see Table 3 for details).

**Table 3:** Aggregated UK Biobank clinical assessments and neuroimaging biomarkers.

Source	Types of Data	Sample Size
**UK Biobank Archive www.ukbiobank.ac.uk**	**Baseline Characteristics**: age, DOB, sex	The collection includes 9,914 subjects with 4,316 clinical/demographic features and 3,297 imaging biomarkers. No longitudinal data are included. The imaging biomarker data are complete, while there is a lot of missing information for the clinical/demographic features. Among the clinical/demographic features, only 23 have complete data, and 1,616 have complete missingness.
	**UK Biobank Assessment Centre:** Interview information, Physical measures, Cognitive function, Imaging: MRI, DXA, Biological sampling: blood, saliva, urine	
	**Questionnaire Information:** Sociodemographics, Life style and environment, Psychosocial factors, Health and medical history, Sex-specific factors.	
	**Biological Samples:** blood, saliva, urine	
	**Genomics:** genotypes, imputation (genotypes/haplotypes), HLA	
	**Online Follow-up:** diet, cognitive function, work environment, mental health.	
	**Additional Exposure:** local environment, physical activity measurement.	
	**Health-related Outcomes:** death, cancer, stroke, myocardial infarction.	
	**Imaging Biomarkers**	
**Challenges**	• Build models to predict these clinical features using imaging biomarkers • Impute the missing data for the clinical/demographic features • Use text mining methods to analyze the free text. Predict clinical features using unstructured data • Validate the prediction model using the hospital admission dataset	

For our CBDA analysis, we then started with this subset of the entire UK Biobank dataset (i.e., 9,914 cases), with 3,297 neuroimaging biomarkers and 4,317 clinical and demographic features. Due to the high frequency of missing data in the clinical/demographic feature subset (~72% of missingness), many of the clinical and demographic features were discarded (see Section 3.4 for details). This data archive includes appropriate and relevant categorical (binomial/binary) outcome features, as well as clinical and neuroimaging measures. Our outcome of interest was irritability (a mood disorder). University of Michigan has signed a materials-transfer-agreement (MTA), 20171017_25641, with the UK Biobank consortium for the use of these UKBB data.

#### 2.5.4. Data availability

Large synthetic datasets can be downloaded using the pipeline workflow script available on our GitHub repository [42]. A client version of LONI pipeline environment [18] can be installed on a local machine and a guest account can be created with the LONI Pipeline Java/WebStart Client [43]. A pipe script can be downloaded from our GitHub repository, in the Data section [42]. After making the appropriate edits to the script in order to point to the appropriate local directories and remote data file name, the selected dataset will be compressed first and then saved in the local directory specified. Otherwise, if a client version is not available, the LONI webapp [44] can be used. Similar edits should be made to the pipeline script before loading on the LONI webapp.

Every synthetic dataset used in this manuscript can be generated from scratch using the R script in the Data Section of our GitHub repository [42]. For larger datasets, it is recommended to use the R script locally. Small size synthetic datasets are available on our GitHub repository [42]. The UK Biobank dataset is not publicly accessible, unless an IRB approval is available. A modified publicly available version of it can be downloaded from our GitHub repository [42]. A proxy of the UK BioBank dataset is publicly available on GitHub; the entire UK Biobank data is available separately (www.ukbiobank.ac.uk).

## 3. Results

We review now all the Results of the CBDA protocol applied to the synthetic datasets first, and then on the real case study datasets. We will describe the feature mining performance, test the proposed performance procedure as well as the two pronged approach in assessing CBDA convergence.

### 3.1 Binomial datasets results

The next sets of results highlight the performance of our CBDA protocol on three synthetic Binomial datasets as described in Table 1 and in the Methods section. Each of the Binomial datasets has only 10 “true” features out of 1,000 and 10,000 features total, respectively. Fig 3A–3C shows the AUC trajectories based on different M* (from 100 up to 5,000) and *α* (from 0.9 down to *e*^−20^). After setting the M*, then True and False Positives, as well as True and False Negatives are calculated for each *α* (displayed as horizontal red lines in Fig 3D–3F). Fig 3D–3F show the correspondent frequency plot for the best M* (selected as the maximum of the AUC trajectories, large circles in black in Fig 3A–3C). For each circle in Fig 3A–3C, a histogram like Fig 3D–3F is generated and, based on the cutoffs calculated on the histogram for M* = 5,000 (e.g., Fig 2A shows the histogram for Fig 3B at M* = 5,000), a Precision-Recall AUC curve is created (e.g., the PR AUC plot for Fig 3B at M* = 1,000 is shown in Fig 2C). For ease of illustration, Fig 3 does not display the cutoff with different colors (as in Fig 2). We used the accuracy of the predictions as performance metric to rank each CBDA predictive model applied to each subsample.

### 3.2 SuperLearner coefficients distributions as indicators of CBDA convergence

Similarly to the analysis performed in Section 3.1, we look at the distribution of the SuperLearner coefficients/weights across the M* top-ranked predictive models to gain insights into CBDA overall convergence. S2 Fig in S1 File shows the results for the Binomial dataset with 10,000 cases and 1,000 features, using Accuracy as performance metric. Similar plots for all the Synthetic datasets analyzed in this study are shown in S3 Fig in S1 File, and they include both Accuracy and MSE as performance metric. For M* = *M* (i.e., 5,000), the coefficients/weights always show a flat distribution (S2A Fig in S1 File), since many of the subsamples do not have any true feature in it, resulting in a poor predictive model. If we only look at the distribution of the M* top-ranked predictive models, some of the algorithms in the SuperLearner library performs better than others (S2B Fig in S1 File). We could use the largest coefficient/weight obtained for M* = *M* as a cut-off for “true positive” algorithms in the SuperLearner library for the optimal M* (as returned by the procedure described in Section 3.1).

To validate this cut-off, we also plot the coefficients/weights distribution obtained from the analysis of the Null dataset. The same cut-off value emerges from the Null dataset analysis (S2C Fig in S1 File). The only difference between the Null and Binomial datasets is that for the Null dataset no “better” algorithm emerges if we only look at the distribution of the M* top-ranked predictive models (S2D Fig in S1 File). Thus, by comparing the SuperLearner coefficients/weights distribution between *M* and M*, we can first check if the CBDA protocol converged to a subset of better-performing algorithms. The overall quality of the CBDA convergence can be then assessed by looking at the overall accuracy as suggested by the Overfitting Test stage of the protocol.

Another way to use the SuperLearner coefficients/weights to gain insights into the CBDA protocol convergence is to compare the Bray-Curtis (BC) dissimilarity index [45] calculated on the Binomial and Null datasets distributions, controlling for M*. S4A-B Fig in S1 File shows the BC index as a function of M*, from 50 to 5,000, calculated on the Binomial dataset with 10,000 cases and 1,000 features. The key information from S4 Fig in S1 File is about the dynamics of the index rather than on its values over M*. The dynamics are unchanged in the Null case if M* is varied, while, in the Binomial case, the lower M* is, the lower the BC index is, suggesting an overall less diverse coefficients/weights distribution. The variance of the coefficients/weights follows a similar pattern when comparing the Binomial (decreasing with a minimum, S4C Fig in S1 File) and the Null (flat, S4D Fig in S1 File) cases. The variance also shows a result consistent with Fig 3A, where the variance of the coefficients/weights can be used to pinpoint the optimal M*. In other words, we can choose M* as the minimum of the variance of the coefficients/weights over M*.

### 3.3 Overfitting test stage

**Fig 4:**
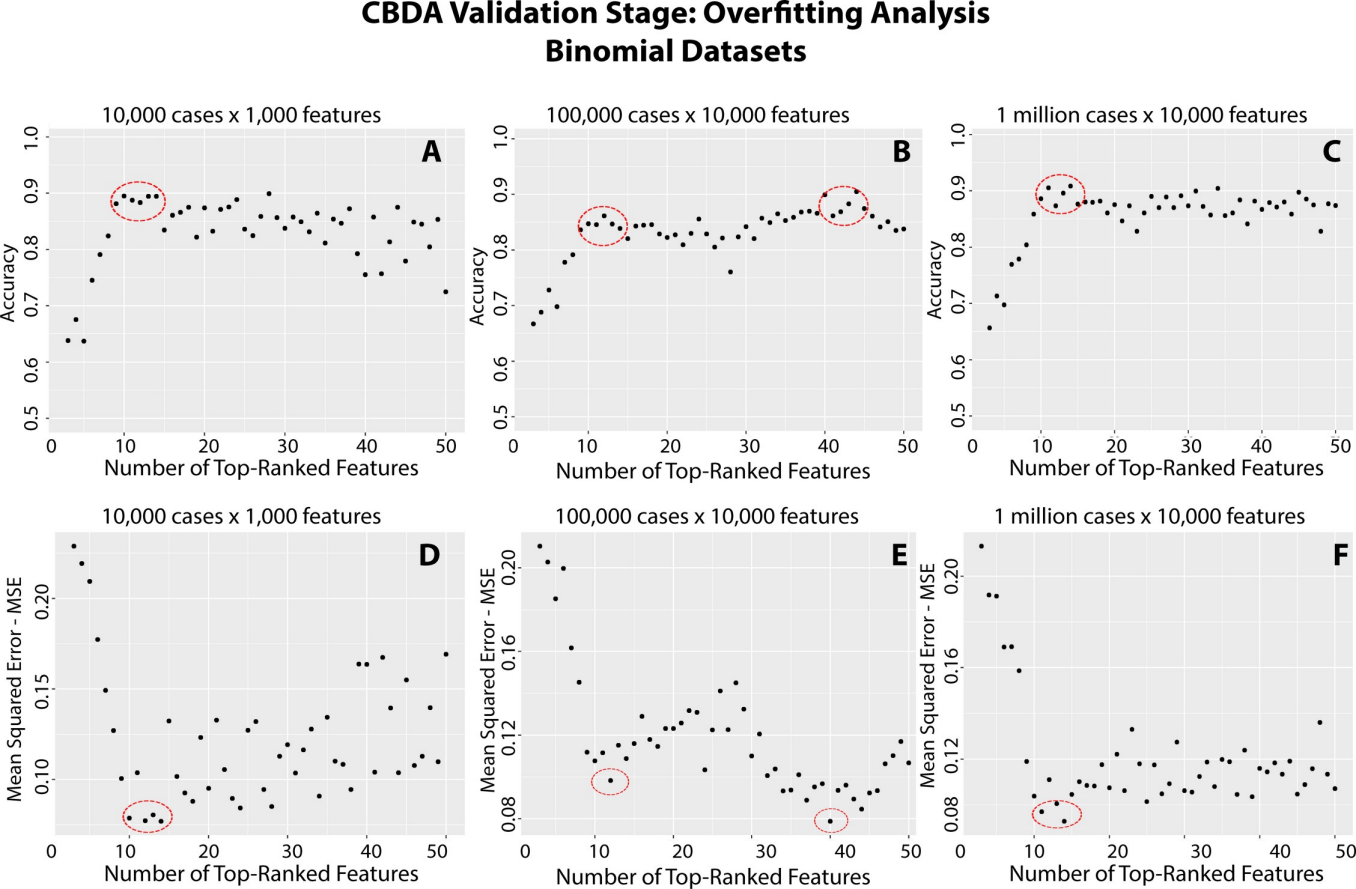
Overfitting analysis for the Binomial datasets. The y-axis shows the performance metric (**Panels A-C**: Accuracy, **Panels D-F**: MSE). The x-axis shows the 50 Top-ranked features resulting from the CBDA Training Stage. These features are used to generate the nested models during the CBDA Overfitting Test Stage and the performance metrics calculations. The red circles identify the likely optimal choices (i.e., max performance without overfitting) for the number of features to include in the best predictive model.

The Overfitting Test Stage (see S1 Fig in S1 File for details) of the CBDA protocol generates nested nonparametric models using the top 50 (or 100) features selected in the training stage. Fig 4 shows the performance metrics Mean Squared Error (MSE) and Accuracy plotted against the number of top features used in each nested predictive model. This example uses the results obtained on the synthetic binomial datasets (as described in Section 2.6). These plots may expose potential overfitting issues. The circles in Fig 4 highlight the optimal number of features to include in the best predictive model, using either Accuracy or MSE as performance criteria. It is worth noting that for the Binomial datasets, the optimal number of top features to be included in the best predictive model is always consistent with the actual number of true features i.e., 10, used to generate the loaded synthetic datasets. In fact, across the three Binomial Datasets, CBDA always ranked at least 8 of the 10 true features among the top 10. As shown by the overfitting plots in Fig 4, having 7–8 true features in the predictive model already ensures an accuracy of ~90%.

### 3.4 Clinical data application to the UK Biobank dataset: Data wrangling stage

We first acquire a subset of cases from the UK Biobank with complete neuroimaging measures (3,297 biomarkers) for a total of 9,914 subjects. Then we expand the data to include all the 4,316 physical features measured on these 9,914 subjects. The resulting initial merged dataset represents a second-order tensor of dimensions 9,914 x 7,614 (with one extra feature representing the subject ID). The next steps were performed to ensure data harmonization and congruency.

For example, we eliminated all the constant features (1,964, all from the subset of physical features), bringing the UKBB dataset down to 2,352 physical features.

**Fig 5:**
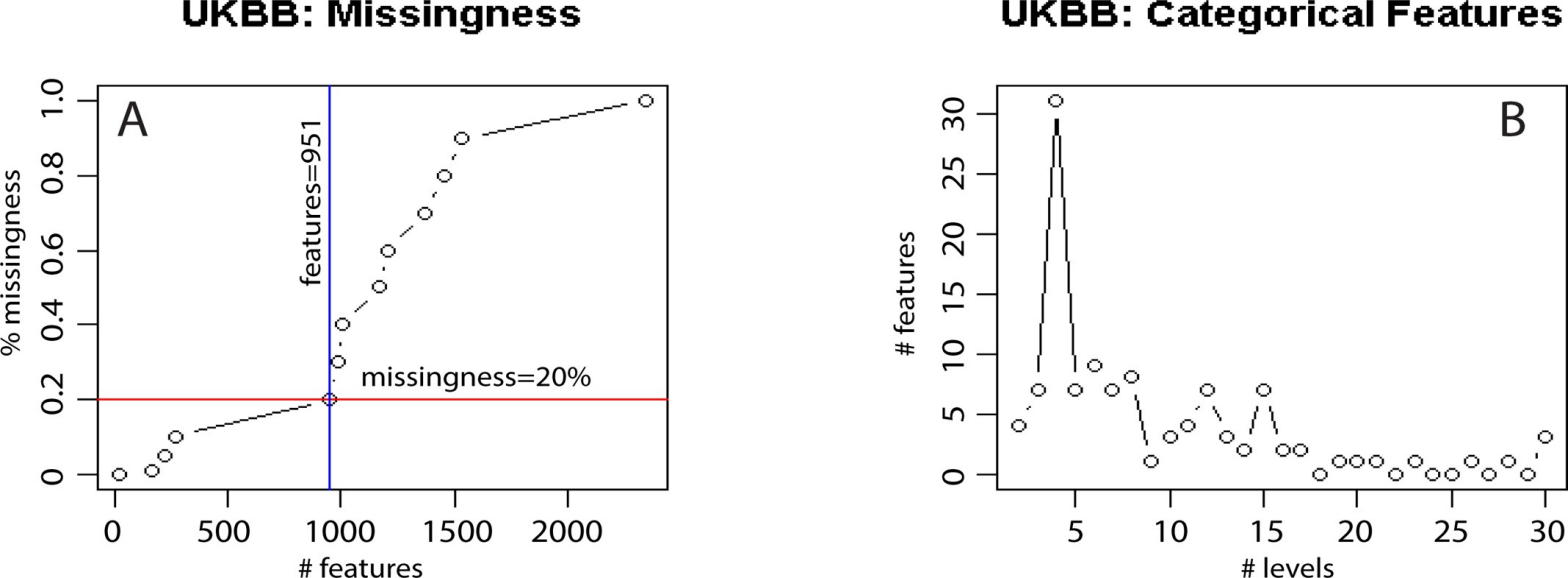
UK Biobank data wrangling statistics and results. Panel A: Missingness analysis. The x axis has the 2,352 features left in the physical dataset after eliminating the constant features. The y axis displays the % of missingness in the dataset, as a function of the number of features added. For example, the red horizontal line indicates a missingness of 20%, and the blue vertical line shows that if we allow for at most 20% missingness in the physical dataset, we end up with 951 features (discarding the other 1,401 features with more than 20% missingness). Panel B: Analysis of the 951 features left in the physical dataset. The x axis shows the number of levels (up to 30) for each of the categorical features in the physical dataset with at most 20% missingness. The goal of this analysis is to identify possible binary target outcomes for the CBDA analysis. The large peak at 4 levels shows features that are actually binary, with some incongruencies (either NAs, or values of -1 and -3 assigned to a binary outcome). The data cleaning step at this stage needs to be done manually and can only rarely be automated. S5 Text in S1 File has some detail on the list of features up to 10 unique levels.

Then we address the magnitude of missingness in the subset of physical features (the neuroimaging subset is complete). The plot below in Fig 5A shows the number of cases/subjects corresponding to different level of missingness. The more missingness we allow the more cases/subjects can be included in the final dataset. However, large missingness can seriously affect our results, no matter how efficient and accurate the imputation is. Based on the landscape shown in Fig 5A, we chose a 20% missingness as the best compromise that allows for a maximum number of additional features with a manageable level of missingness (increasing the missingness to 30% or 40% will only add approximately 50 subjects). Only 19 features are complete (see the list in S5 Text in S1 File).

The subset of physical features was further reduced to 951 features (still 9,914 cases).

Our goal was to clean the data as much as possible before making it available to the CBDA protocol. Thus, the next data wrangling step analyzed the unique values for each feature, merging categorical and numeric (both integer and double) features. Fig 5B shows the number of features that have certain number of levels (or unique values), starting from 2 up to 30. We did not include the missing values code “*NA*” as a level. Fig 5B shows a peak at levels = 4 with 31 features.

Due to the incongruency of the UKBB dataset, some obvious binary features are often coded with 4 levels (e.g., 0, 1, −1, −3), and the same *Field code* or ID is used multiple times for different time points. For these features, we eliminated the levels -1 and -3. A future CBDA protocol update will include a new module to address time-varying longitudinal data. We treated these pseudo-longitudinal data fields as the same feature and depending on the type of the measure, either exclude them or use their mean values. This extra “cleaning” step reduced the physical features to 830. In general, such ad-hoc pre-processing steps cannot be automated, as they require specific knowledge of the case-study, especially when the outcome of interest shows incongruences.

Another important step is to ensure that any of the features included as “predictors” for the outcome of interest are not correlated to the outcome of interest (e.g., they measure the same or similar outcome). For example, the two features “*X2090*: *seen doctor for nerves*, *anxiety*, *tension or depression*" and “*X4598*: *ever depressed for a whole week*” are highly correlated, and using one as the outcome of interest should exclude the other one from the set of predictors. An exhaustive and automated search will require adequate handling of unstructured data, which we will include in a separate dedicated module of the CBDA protocol. To demonstrate the CBDA protocol performance, for this UK Biobank study, we chose outcome of interest Irritability (namely "*X1940*: *Irritability*") as the outcome of interest. The initial levels for “*Irritability*”, −1, −3, 0, 1, were transformed a binary outcome by eliminating the irrelevant levels −1 and −3. The physical features labels and counts for up to 10 unique levels are shown in S5 Text in S1 File. The final UK Biobank subset analyzed to predict the outcome of interest Irritability is 9,569 cases/subject with a total of 4,129 combined features: ID and outcome of interest (i.e., Irritability), 3,297 neuroimaging biomarkers and 830 physical features, the latter with at most 20% missing values.

### 3.5 CBDA applied to the UK Biobank dataset

**Fig 6:**
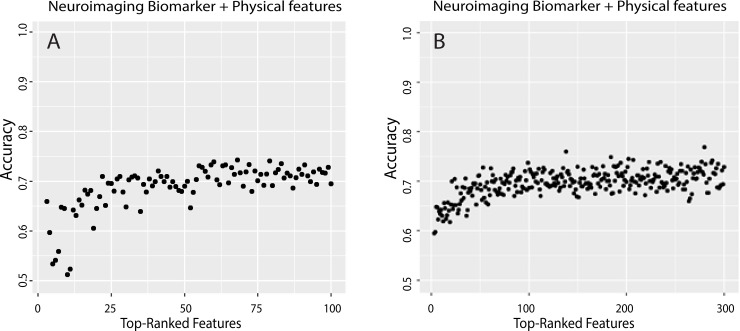
Overfitting plots for the UK Biobank dataset CBDA analysis. The x axis shows the top 100 (Panel A) and top 300 (Panel B) features returned by the CBDA training stage in predicting the outcome of interest Irritability on the UK Biobank dataset with both neuroimaging and clinical biomarkers (see S1 Fig in S1 File for details). The y axis represents the accuracy of the nested models after the CBDA Overfitting Test stage. The details on the features can be found in the S2 Table (for Panel A) and S4 Table (for Panel B) in S1 File.

We applied the new CBDA functions to assess the CBDA performance during the training (i.e., *CBDA_slicer()*, *BCplot()* and *SLcoef_plot()*) and validation (*Overfitting_plot()*) stages. Ultimately the *Overfitting_plot()* results will determine the overall performance of the CBDA protocol on each dataset. Fig 6A shows the accuracy results of CBDA protocol for the top 100 features returned by the CBDA Overfitting Test stage executed on the neuroimaging biomarkers and the physical features as predictors. S3A Fig in S1 File shows the equivalent plot for the neuroimaging biomarkers only.

The overall accuracy in predicting the outcome of interest Irritability converges to approximately 72% for both datasets, with a slightly different dynamic when only the top 5–10 features are included. S1 and S2 Tables in S1 File show the lists of the top 100 features for both datasets analysis. When the physical features are included in the analysis, only 5 of them are selected in the top 100 (ranked from 20 to 24), and they all refer to neuroimaging features (i.e., see the grey cells in the S2 Table in S1 File, for details). There is a minimal overlap if we compare the top100 features, namely 4 features overlap between the 2 datasets analyses (i.e.,"rh_BA45_exvivo_thickness", "rh_middletemporal_meancurv", "lh_temporalpole_gauscurv", "lh_S_circular_insula_ant_curvind").

The highly correlated features in the biomarker dataset can explain this lack of overlap. A pairwise correlation analysis of all the neuroimaging biomarkers shows that 75% of the top 200 features (resulting after merging the 2 lists displayed in S5 Text in S1 File) have a correlation of 0.37 and higher, 50% of the top 200 features have a correlation of 0.55 and higher, 20% of the top 200 features have a correlation of 0.72 and higher, and 10% of the top 200 features have a correlation exceeding 0.82.

We ran the analysis again increasing the FSR up to approximately 180, using a *Variance Inflation Factor* (VIF) of 6. The VIF for the UKBB dataset is determined by the peculiar structure of the biomarker measures. In fact, the same measures are divided between a left and right hemisphere (factor of 2) and of approximately 15 measures on each region of interest that are correlated (e.g., volume, surface, thickness). We assumed a 20% of the number of measures for each ROI to be highly correlated and contribute to an additional scaling factor of 3 (i.e., 20% of 15). Thus, the VIF is calculated as 2×3 = 6, bringing the FSR from 30 to 180. Increasing the FSR further will significantly slow down the CBDA protocol without any advantages in terms of performance.

The results of the CBDA analysis with the VIF = 6 are shown in S3 and S4 Tables in S1 File, where the top 300 selected features are listed for both datasets, respectively. There is an overlap of 34 features between the two analyses (see S5 Table in S1 File for details). S3B Fig in S1 File shows the equivalent of Fig 6B with neuroimaging biomarkers only included in the analysis. Increasing the FSR does not change the results in terms of overlap. If we compare the two CBDA experiments with different FSR, among the top 100 and top 300, there are 19 overlapping features for the experiment using only neuroimaging biomarkers and 9 overlapping features when using both neuroimaging and clinical biomarkers. The accuracy does not change significantly over the top 50 features (although it slightly increases, see Fig 6). In the presence of highly correlated features, the inclusion of additional features does not affect the performance, especially if the pool of the remaining features is highly correlated. Possibly repeating the CBDA experiment a large number of times could shed some light into the correlation structure of the dataset with respect to the most predictive features, alone and in multivariate combinations.

S6 Fig in S1 File shows the *SLcoef_plot()* and *BCplot()* functions output for the UK Biobank dataset with neuroimaging biomarker and physical features. The SuperLearner coefficients distribution shows how the SVM algorithm (with different kernel specifications) is consistently the most adequate to analyze the dataset (S6A-B Fig in S1 File). In fact, across the 55 different classification and machine learning algorithms bagged into the SL.library of our ensemble predictor, the SVM class has the best predictive power. S6A Fig in S1 File shows the mean SuperLearner coefficients assigned during training across the 5,000 subsamples. S6B Fig in S1 File enforces a threshold of 0.05, however most of the algorithms’ coefficients fell well below that, as shown in S6A Fig in S1 File. No specific insights can be gained by looking at the Bray-Curtis and variance plots. The Bray-Curtis dissimilarity trajectory generated by the function *BCplot()* is relatively flat (except for an increase for M*<500), with a set of minima between M* = 1,000 and M* = 3,000 (S6C Fig in S1 File). The variance of the SuperLearner coefficients is consistently decreasing when M* decreases from 5,000 down to 50 (S6D Fig in S1 File). The analysis on the UK Biobank with only the neuroimaging biomarkers returns similar results.

## 4. Discussion and conclusions

There are many challenges and opportunities associated with Big Data and team-based scientific discovery. Key components in this knowledge discovery process are curation, analysis, domain-independent reproducibility, area-specific replicability, organization, management, and sharing of health-related digital objects.

Open-science offers a promising avenue to tackle some of these challenges. The FAIR data principles that we abide by (making data Findable, Accessible, Interoperable and Reusable) [46] promote maximum use of research data and foster continuous development of new methods and approaches to feed data driven discovery in the biomedical and clinical health sciences, as well as in any Big Data field [47].

This work expands the functionality and utility of a new ensemble semi-supervised machine learning technique called Compressive Big Data Analytics (CBDA). We designed and built the CBDA protocol following the FAIR open-source/open-science principles where the scientific community can independently test, validate and expand on our second generation technology. The entire protocol, the updated R software package [4, 20] and the complete high performance computing (HPC) workflow (i.e., LONI pipeline, see [21] for details) are openly shared and publicly accessible on our GitHub repository [19]. As in our previous release (V 1.0), CBDA 2.0 has two open-source implementations: (1) a platform-agnostic stand-alone R package, and (2) a reproducible pipeline graphical workflow (wrapper of the R-package).

In an effort to make the CBDA protocol accessible to a larger pool of researcher so it can be deployed on virtually any HPC server, we are working now on recasting the LONI pipeline workflow into more popular and commonly used batch systems like PBS and SLURM. Currently the pre- and post-processing steps to efficiently perform subsampling are developed as shell and Perl scripts. In order to better and more efficiently handle heterogeneous, unstructured and incongruent data types, we are recasting the scripts for these two critical steps into the Python language.

Currently, time-varying longitudinal and unstructured data require preprocessing before CBDA 2.0 analytics. We are developing methods and approaches to address these challenges in the context of data privacy and utility [47, 48]. We will incorporate the findings and the corresponding R wrappers implementation into the CBDA protocol as soon as they are sufficiently tested and validated. A synoptic summary of current and future developments for the CBDA R packages is illustrated in Table 4.

**Table 4:** Past, present and future CBDA R package developments.

	CBDA R 1.0 [past]	CBDA R 2.0: Large Scale [current]	CBDA R 3.0: Unstructured and Longitudinal Data [future]
***Client implementation*: single and multicore options.**	*Client implementation*: single and multicore options^(*)^.		
***HPC implementation***	*A*vailable for LONI pipeline only.		*A*vailable for PBS, SLURM and LONI pipeline only.
***Client and Server implementation*: *Big Data loading***	*L*oad the Big Data into the R workspace before any CBDA subsampling and training/validation ^(*)^.	*D*oes not load the Big Data into the R workspace.	
***Client and Server implementation*: subsampling**	*Server implementation*: subsampling (both for the CBDA Training and Overfitting Test stages) is done internally in R^(*)^	*Server implementation*: subsampling (both for the CBDA Training and Overfitting Test stages) is performed by a combination of shell and Perl scripts^(**)^	*Server implementation*: subsampling (both for the CBDA Training and Overfitting Test stages) is performed by Python scripts
**Unstructured and Longitudinal Data Types**	Does not support Longitudinal and Unstructured Data	Handles Longitudinal and Unstructured Data	

(*) Not recommended for datasets larger than 1GB.

(**) Faster if subsampling and predictive analytics is performed in the same location as the original data.

We tested the second generation CBDA protocol on both synthetically generated and real clinical datasets. Results on synthetically generated datasets confirm and strengthen our previous study. Even with significantly reduced feature undersampling rates (e.g., from ~1%−5%, down to ~0.03%−0.3%), and increased sizes of the datasets analyzed (e.g., up to 1 million cases and 10,000 features), the CBDA protocol can identify most of the true features. The new CBDA functionalities enable assessment of overfitting and possibly convergence issues. The results of applying the new *Overfitting_plot()* function to the synthetic datasets show that accurate predictions can be generated even if only a subset of the true features is mined and selected.

The CBDA classification results on the UK Biobank population-wide (census-like) study provide empirical evidence of effective prediction, especially when the data is extremely complex, incongruent, higly-correlated, and include a lot of missing records. Overall, CBDA performs well in predicting the outcome of interest, *Irritability*, in the presence of highly-correlated features. Multi-collinearity plays a key role in analyzing the UK Biobank. When the data includes extremely correlated multivariate features, CBDA reproducibility for small number of subsample sizes may naturally exhibit variability of the results, due to uncharacteristic groupings of independent or highly associated clusters of features. In the UKBB study, once the accuracy in predicting the outcome of interest (Irritability) reaches ~70% (fairly quickly, using top 10–20 features), the additional features tend not to alter or improve the overall performance. The top 10–20 features are selected semi-randomly among the top 100–300 non-independent covariates. The ranking is based on random subsampling and the feature selection in such a scenario (highly correlated features) is affected by accuracy values that are very close to each other.

These results illustrate the scalability, efficiency and potential of CBDA to *compress* complex data into structural information. This leads to derived knowledge and translational action, where specific clinical outcomes can be targeted. Combining the CBDA and the UKBtools [49] R packages in the next wave of analysis may streamline the analytical protocol. Such tool-interoperability facilitates the mapping of features, their descriptions and field ID codes, improves the necessary data cleaning and wrangling before CBDA is implemented, and allows for post-hoc analytics.

This exploratory CBDA study paves the way for a deeper analysis of the UK Biobank archive. Our results may also suggest potentially new avenues of research in the context of identifying, tracking, and treating mental health and aging-related disorders, where a priori clinical phenotypes and inherently correlated multivariate predictors may not necessarily be explicitly known.

## Supporting information

S1 File (DOCX)
