# Supplementary material for: Compressive Big Data Analytics: An ensemble meta-algorithm for high-dimensional multisource datasets
Source: PLoS One. 2020 Aug 28;15(8):e0228520. doi: 10.1371/journal.pone.0228520 (PMC7455041; doi:10.1371/journal.pone.0228520)

## SUPPLEMENTARY MATERIALS

## Supplementary Text S1: CBDA Mathematical Formulation

###

### Step 1 Data Cleaning

Let’s label each original dataset as $DSorigin_{p}(p=1,2,...,n)$, and define a data cleaning algorithm as C. Then. $DSclean_{p}=C(DSorigin_{p}),p=1,2,...,n$.

###

### Step 2 Data Harmonization

Define a data harmonization algorithm by $H$. Then, $DS_{p}=H(DSclean_{p}),p=1,2,...,n$, where each $DS_{p}=[X_{p},Y_{p}],p=1,2,...,n$ has the same dimension.

###

### Step 3 Data Aggregation

We define the large dataset $B=[X,Y]\in R^{N\times K}$, which is an aggregation of $DS_{p},p=1,2,...,n$,and where $N$ represents the total number of cases and $K$ represents the total number of features.

Our goal is to find the dictionary matrix $\Phi$ to map the most important features for our predictive model.

###

### Step 4 Random Sampling and Selection of Prediction Dataset

As described earlier in the Methods section, for each sample $j,(j=1,2,3,...,M)$, of the Big Data $B=[X,Y]\in R^{N\times K}$, we sample training datasets ${[X}^{j},Y^{j}]$ with a fix number of cases $n_{j}$ and features $k_{j}$, namely $n_{j}=300$ and $k_{j}=30$. The sample validation datasets ${[X}_{val}^{j},Y_{val}^{j}]$ have $2n_{j}$ number of cases and the same features selected for the training dataset $X^{j}$. Since we are dealing with very large datasets B, the number $n_{j}$ and $k_{j}$ can always be fulfilled. Thus, for each sample $j$, we have the following 4 datasets:

$\left[ X^{j},Y^{j},X_{val}^{j},Y_{val}^{j} \right]$,

where $X^{j}\in R^{n_{j}\times k_{j}},Y^{j}\in R^{n_{j}\times1},X_{val}^{j}\in R^{2n_{j}\times k_{j}},Y_{val}^{j}\in R^{{2n}_{j}\times1}$, with $k_{j}\ll K$ and $n_{j}⋘N$. There is no overlap between the $n_{j}$ cases of $X^{j}$ and the 2$n_{j}$ cases of $X_{val}^{j}.$ Each sample $j$ is sampled with replacement.

###

### Step 5 Data Imputation, Scaling and Re-balancing (optional)

This step is optional and may or may not be necessary depending on the specific case-study.

### *Imputation*

If $X^{j}$ or $X_{val}^{j}$ has missing values as $X_{miss}^{j}$ or $X_{val-miss}^{j},$ we define the R function 'missForest' [51] as $MF$, then we denote

$MF:R^{n_{j}\times k_{j}}\to R^{n_{j}\times k_{j}}$*,* and $X^{j}:=MF(X_{miss}^{j})$ or $X_{val}^{j}:=MF(X_{val-miss}^{j})$*.* Here $X^{j}$ *and* $X_{val}^{j}$ are complete data samples*.*

### *Scaling*

If $X^{j}$ or $X_{val}^{j}$ need to be normalized as $\hat{X}^{j}$, or $\hat{X}_{val}^{j}$ we define a normalization function, $N$. (Normalization functions are different, which to choose depends on demands). Here we take a normalization function for example, which makes each column average equal to 0 and the corresponding standard deviation equal to 1: $N:R^{n_{j}\times k_{j}}\to R^{n_{j}\times k_{j}}$,$X^{j}:=N\left( \hat{X}^{j} \right)$, then the $m^{th}$ row and $p^{th}$ column element in $X^{j}$ (or $X_{val}^{j})$ can be expressed as:

.

### *Cohort Re-balancing*

If the data samples are imbalanced as $[X^{j},Y^{j}]_{imbal}\in[R^{\hat{m}_{j}\times k_{j}},R^{\hat{m}_{j}\times1}]$, we use a statistical rebalancing strategy, like the Synthetic Minority Over-sampling Technique (SMOTE) [52] to approximately equalize the group sizes. Let’s denote that by:

$SMOTE:[R^{\hat{m}_{j}\times k_{j}},R^{\hat{m}_{j}\times1}]\to[R^{m_{j}\times k_{j}},R^{m_{j}\times1}]$, where $m_{j}\geq\hat{m}_{j}$.

###

### Step 6 SuperLearner Algorithm

We define SuperLearner algorithm as ML:

$ML_{j}(C^{j}):R^{n_{j}\times k_{j}}\times R^{n_{j}\times1}\times R^{2n_{j}\times k_{j}}\to R^{{2n}_{j}\times1}$,

$ML_{j}([X^{j},Y^{j},X_{val}^{j}])=\hat{y}^{j}$.

Here we separate the SuperLearner algorithm step into two parts (learning and validation) to obtain a clearer understanding of its effects.

### *Training/Learning*

Let's define the matrix $X\in\boldsymbol{X}$ and the vector $Y\in\boldsymbol{Y}$. Here $X=R^{n_{j}\times k_{j}}$ and $Y=R^{n_{j}\times1}$ (column vector).

We then define $A^{*}$ as the dual space of $\boldsymbol{A}$, here $A=R^{1\times k_{j}}$,and $A^{*}$ includes a finite set of statistical learning algorithms as:$\forall F\in A^{*}$, $F:R^{1\times k_{j}}\to R$.

We then define the Descartes product between $X$ and $Y$ as: $\boldsymbol{X} \bigotimes\boldsymbol{Y}$. The element in $\boldsymbol{X} \bigotimes\boldsymbol{Y}$ has the form of $(X, Y)$. We can express the black box learning process as a function $\phi$:

$\phi:\boldsymbol{X} \bigotimes\boldsymbol{Y}$ $\to A^{*}$, $\phi((X,Y))(a)=F(a)$, where $a\in A$.

For each $j$, $\phi((X^{j},Y^{j}))(a)=F^{j}(a)$. As an example, we can think of $\phi$ as the optimization algorithm embedded into our SuperLearner, with $A^{*}$ the set of algorithms specified in the SuperLearner library.

### *Overfitting Test Stage*

After we obtain the $F^{j}$, we apply it to $X_{val}^{j}$. Namely, $F^{j}(X_{val}^{j})\triangleq\hat{y}^{j}$. Here, $F^{j}$ is function on $X_{val}^{j}$ acting one column at a time, thus $F^{j}$ is a function on $R^{1\times k_{j}}$ outputting $\hat{Y}^{j}\in R^{2n_{j}\times1}$.

###

### Step 7 Ranking

We compare the $\hat{Y}^{j}$ with the $Y_{val}^{j}$ using a performance metric $\tau$ (e.g., accuracy, MSE, etc.) Let’s denote the performance by:

$c_{j}=\tau(\hat{Y}^{j},Y_{val}^{j}),j=1,2,...,M$.

For example, when $\tau$ is MSE, $c_{j}=MSE_{j}$, and when $\tau$ is Accuracy, $c_{j}=Accuracy_{j}$.

Then we apply the following operators:

1. Rank all the $\{c_{j}{\}}_{j=1,2,...,M}$.
2. Select of the top $\{c_{j}{\}}_{j=1,2,...,M}$.

These two steps may be denoted by the following operator: $\{c_{\left( j \right)}{\}}_{j=1}^{q}=O^{q}(\{c_{j}\})$.

###

### Step 8 Feature mining

Let’s define $\Omega$ to be the set of all the features $f_{i}$, then $\Omega=\{f_{i}\},i=1,2,...,K$. The next step involves first counting and then ranking the occurrences of each $f_{i}$ across the top $\{c_{\left( j \right)}{\}}_{j=1}^{q}$. This is expressed by:

1. Setting 1 if the feature occurs and 0 if not, here $j=1,2,...,q$.

$$s_{j}^{(i)}=Dirichlet_{j}\left( f_{i} \right)=\left\{ \begin{matrix} 0, f_{i}\notin sample j \\ 1, f_{i}\in sample j \end{matrix} \right..$$

2. Counting occurrences of each $f_{i}$ across the top q$\{c_{(j)}{\}}_{j=1,2,...,q}$ by:

$S_{i}=\underset{j}{Sum}(s_{j}^{(i)}),i=1,2,...,K$.

1. Ranking the occurrences of each $f_{i}$ and obtain new ranked feature set:

$S_{(i)}=O^{K}(S_{i}),i=1,2,...,K$.

$\Omega^{*}=\{f_{1},...,f_{i},...,f_{K}\}$.

We can also formalize the above protocol as follows: Set up the matrix $F\in R^{q\times K}$, where the rows represent the top samples and the columns represent the features. Then, $\forall b_{ji}\in F,b_{ji}=s_{j}^{(i)}$ and $S_{i}=\sum_{j}^{q} b_{ji},i=1,2,...,K$.

The inference step takes as input the $\Omega^{*}$ defined above and our goal in the inference step is to define the optimal dimension of the dictionary matrix $\Phi$, where we plug the most important features into our predictive model. One way to automate the selection of how many features to include in our predictive model is to find the minimal set of features $l$ that ensures the best performance metric $\tau$. To do so, we run the following post-optimization feature mining scheme on $\Omega^{*}$ the top features. We can make this step more efficient by considering an example of choosing 5 features at a time within the features index $p$:

- - for $p=1$ we select the top 5 features $k_{1}^{*}=5$
  - for $p=2$ we select the top 10 features $k_{2}^{*}=10$
  - ....., and so on.
  - for we select the top $P$ features (stop here).

Here $k_{p}^{*}$ represents the number of top features that we chose and $p=\frac{P}{5}$. Alternatively, we can simply add one feature at a time, starting from a minimal set of features that we want to be built into our predictive model (e.g., the minimal model will start from the top 3 features from $\Omega^{*}$). Also, $P$ can be chosen to be a function of the total number of features $K$ (e.g., $3+3*log(K)$, with $3$ being the number of features in our minimal predictive model).

Then we have:

$Y_{val}^{p}\in R^{m}.$

If the performance metric is given by $\tau(Y_{val}^{p},Y_{val}^{j})$, we can select the $Best_{p=1,2,.....,l}(\tau(Y_{Val}^{p},Y_{val}^{j}))$. For instance, if we use $\tau=Accuracy=A$ as our performance metric, we have:

$A(Y_{val}^{p},Y_{val}^{j}),p=1,2,...,\hat{p}$ .

Then, we can select the set of features to include in the “best” predictive model by following the stopping criteria outlined below. We can also plot $A(Y_{Val}^{p},Y_{val}^{j})$ against the number of features $k_{p}^{*}$ and use the plot to guide our selection of the “best” set of features.

A similar strategy can be implemented if we use $\tau=MSE$ as our performance metric, where we have:

$MSE(Y_{val}^{p},Y_{val}^{j})=\frac{1}{m}\parallel Y_{val}^{p}-Y_{val}^{j}\parallel_{2}^{2}$.

### Stopping Criteria

To avoid overfitting, we can implement several alternative strategies. For example, if we use $\tau=\mathrm{Accuracy}=A$ as our performance metric, we can set a minimal threshold for $A$ (i.e., $A>70$) and

1. Stop if $A(t+1)<=A(t)$, where $A(t+1)$ has 1 more features than $A(t)$ in the predictive model
2. Stop if $A(t+1)/A(t)-1<\lambda$ (with $\lambda=$ 0.1, 0.05 or 0.01). This step ensures at least a 10% or 5% or 1% improvement.

If we use $\tau=MSE$ as our performance metric, we can implement the following stopping criteria:

1. Stop if $MSE(t+1)\geq MSE(t)$, where $MSE(t+1)$ has 1 more features than $MSE(t)$ in the predictive model.
2. Stop if $MSE(t+1)/MSE(t)>F(n_{1},n_{2},\alpha)$, where $F$ represents the Fisher’s F-test, with degrees of freedom $n_{1}$ and $n_{2}$, respectively, and $\alpha$ the level of significance of the test. This test ensures that the 2 $MSEs$ are significantly different from each other. In other words, we want to be sure that adding an extra feature to our predictive model will make a significant impact in reducing the metric $\tau=MSE$.

We can also simply plot the performance metric as a function of the number of top features and evaluate the optimal number of features as the max (for the Accuracy metric) or the min (for the MSE metric) value of the metric (see Figure 4 and 6 in the main manuscript as an example).

## Supplementary Text S2: Description and Default Parameters of the Classification Algorithms included in the SuperLearner library of the CBDA protocol

**Support Vector Machine – SL.svm**

svm(x, y = NULL, scale = TRUE, type = NULL, kernel ="radial",

degree = 3, gamma = if (is.vector(x)) 1 else 1 / ncol(x),

coef0 = 0, cost = 1, nu = 0.5, class.weights = NULL, cachesize = 40, tolerance = 0.001,

epsilon = 0.1,shrinking = TRUE, cross = 0, probability = FALSE, fitted = TRUE,

..., subset, na.action = na.omit)

***Description***

| x | a data matrix, a vector, or a sparse matrix (object of class [Matrix](http://127.0.0.1:23359/help/library/Matrix/html/Matrix.html) provided by the **Matrix** package, or of class [matrix.csr](http://127.0.0.1:23359/help/library/SparseM/html/matrix.csr.html) provided by the **SparseM** package, or of class [simple_triplet_matrix](http://127.0.0.1:23359/help/library/slam/html/simple_triplet_matrix.html) provided by the **slam** package). |
| --- | --- |
| y | a response vector with one label for each row/component of x. Can be either a factor (for classification tasks) or a numeric vector (for regression). |
| scale | A logical vector indicating the variables to be scaled. If scale is of length 1, the value is recycled as many times as needed. Per default, data are scaled internally (both x and y variables) to zero mean and unit variance. The center and scale values are returned and used for later predictions. |
| type | svm can be used as a classification machine, as a regression machine, or for novelty detection. Depending of whether y is a factor or not, the default setting for type is C-classification or eps-regression, respectively, but may be overwritten by setting an explicit value. Valid options are:   - C-classification - nu-classification - one-classification (for novelty detection) - eps-regression - nu-regression |
| kernel | the kernel used in training and predicting. You might consider changing some of the following parameters, depending on the kernel type.  linear:  *u'*v*  polynomial:  *(gamma*u'*v + coef0)^degree*  radial basis:  *exp(-gamma*|u-v|^2)*  sigmoid:  *tanh(gamma*u'*v + coef0)* |
| degree | parameter needed for kernel of type polynomial (default: 3) |
| gamma | parameter needed for all kernels except linear (default: 1/(data dimension)) |
| coef0 | parameter needed for kernels of type polynomial and sigmoid(default: 0) |
| cost | cost of constraints violation (default: 1)—it is the ‘C’-constant of the regularization term in the Lagrange formulation. |
| nu | parameter needed for nu-classification, nu-regression, and one-classification |
| class.weights | a named vector of weights for the different classes, used for asymmetric class sizes. Not all factor levels have to be supplied (default weight: 1). All components have to be named. Specifying "inverse" will choose the weights *inversely* proportional to the class distribution. |
| cachesize | cache memory in MB (default 40) |
| tolerance | tolerance of termination criterion (default: 0.001) |
| epsilon | epsilon in the insensitive-loss function (default: 0.1) |
| shrinking | option whether to use the shrinking-heuristics (default: TRUE) |
| cross | if a integer value k>0 is specified, a k-fold cross validation on the training data is performed to assess the quality of the model: the accuracy rate for classification and the Mean Squared Error for regression |
| fitted | logical indicating whether the fitted values should be computed and included in the model or not (default: TRUE) |
| probability | logical indicating whether the model should allow for probability predictions. |
| ... | additional parameters for the low level fitting function svm.default |
| subset | An index vector specifying the cases to be used in the training sample. (NOTE: If given, this argument must be named.) |
| na.action | A function to specify the action to be taken if NAs are found. The default action is na.omit, which leads to rejection of cases with missing values on any required variable. An alternative is na.fail, which causes an error if NA cases are found. (NOTE: If given, this argument must be named.) |

**SL.svm() wrappers used in the SuperLearner library of the CBDA protocol**

SL.svm.radial.10 <- function(..., family="binomial", kernel = "radial", gamma = 10){

SL.svm(..., family = family, kernel = kernel, gamma = gamma)

}

SL.svm.radial.0.1 <- function(...,family="binomial", kernel = "radial", gamma = 0.1){

SL.svm(...,family = family, kernel = kernel, gamma = gamma)

}

SL.svm.radial.default <- function(...,family="binomial", kernel = "radial"){

SL.svm(...,family = family, kernel = kernel)

}

SL.svm.poly.2.0 <- function(...,family="binomial", kernel = "polynomial", gamma = 1, coef0 = 0, degree = 2){

SL.svm(...,family = family, kernel = kernel, gamma = gamma, coef0 = coef0, degree = degree )

}

SL.svm.poly.3.0 <- function(...,family="binomial", kernel = "polynomial", gamma = 1, coef0 = 0, degree = 3){

SL.svm(...,family = family, kernel = kernel, gamma = gamma, coef0 = coef0, degree = degree )

}

SL.svm.poly.3.10 <- function(...,family="binomial", kernel = "polynomial", gamma = 1, coef0 = 10, degree = 3){

SL.svm(...,family = family, kernel = kernel, gamma = gamma, coef0 = coef0, degree = degree )

}

SL.svm.poly.3.n10 <- function(...,family="binomial", kernel = "polynomial", gamma = 1, coef0 = -10, degree = 3){

SL.svm(...,family = family, kernel = kernel, gamma = gamma, coef0 = coef0, degree = degree )

}

SL.svm.poly.6.0 <- function(...,family="binomial", kernel = "polynomial", gamma = 1, coef0 = 0, degree = 6){

SL.svm(...,family = family, kernel = kernel, gamma = gamma, coef0 = coef0, degree = degree )

}

SL.svm.poly.6.10 <- function(...,family="binomial", kernel = "polynomial", gamma = 1, coef0 = 10, degree = 6){

SL.svm(...,family = family, kernel = kernel, gamma = gamma, coef0 = coef0, degree = degree )

}

SL.svm.poly.6.n10 <- function(...,family="binomial", kernel = "polynomial", gamma = 1, coef0 = -10, degree = 6){

SL.svm(...,family = family, kernel = kernel, gamma = gamma, coef0 = coef0, degree = degree )

}

SL.svm.linear <- function(...,family="binomial", kernel = "linear"){

SL.svm(...,family = family, kernel = kernel)

}

SL.svm.sigmoid <- function(...,family="binomial", kernel = "sigmoid", gamma = 1, coef0 = 0){

SL.svm(...,family = family, kernel = kernel, gamma = gamma, coef0 = coef0)

}

**Random Forest - SL.randomForest**

randomForest(x, y=NULL, xtest=NULL, ytest=NULL, ntree=500,

mtry=if (!is.null(y) && !is.factor(y))

max(floor(ncol(x)/3), 1) else floor(sqrt(ncol(x))),

replace=TRUE, classwt=NULL, cutoff, strata,

sampsize = if (replace) nrow(x) else ceiling(.632*nrow(x)),

nodesize = if (!is.null(y) && !is.factor(y)) 5 else 1,

maxnodes = NULL,

importance=FALSE, localImp=FALSE, nPerm=1,

proximity, oob.prox=proximity,

norm.votes=TRUE, do.trace=FALSE,

keep.forest=!is.null(y) && is.null(xtest), corr.bias=FALSE,

keep.inbag=FALSE, ...)

***Description***

| x | a data frame or a matrix of predictors. |
| --- | --- |
| y | A response vector. If a factor, classification is assumed, otherwise regression is assumed. If omitted, randomForest will run in unsupervised mode. |
| xtest | a data frame or matrix (like x) containing predictors for the test set. |
| ytest | response for the test set. |
| ntree | Number of trees to grow. This should not be set to too small a number, to ensure that every input row gets predicted at least a few times. |
| mtry | Number of variables randomly sampled as candidates at each split. Note that the default values are different for classification (sqrt(p) where p is number of variables in x) and regression (p/3) |
| replace | Should sampling of cases be done with or without replacement? |
| classwt | Priors of the classes. Need not add up to one. Ignored for regression. |
| cutoff | (Classification only) A vector of length equal to number of classes. The ‘winning’ class for an observation is the one with the maximum ratio of proportion of votes to cutoff. Default is 1/k where k is the number of classes (i.e., majority vote wins). |
| strata | A (factor) variable that is used for stratified sampling. |
| sampsize | Size(s) of sample to draw. For classification, if sampsize is a vector of the length the number of strata, then sampling is stratified by strata, and the elements of sampsize indicate the numbers to be drawn from the strata. |
| nodesize | Minimum size of terminal nodes. Setting this number larger causes smaller trees to be grown (and thus take less time). Note that the default values are different for classification (1) and regression (5). |
| maxnodes | Maximum number of terminal nodes trees in the forest can have. If not given, trees are grown to the maximum possible (subject to limits by nodesize). If set larger than maximum possible, a warning is issued. |
| importance | Should importance of predictors be assessed? |
| localImp | Should casewise importance measure be computed? (Setting this to TRUEwill override importance.) |
| nPerm | Number of times the OOB data are permuted per tree for assessing variable importance. Number larger than 1 gives slightly more stable estimate, but not very effective. Currently only implemented for regression. |
| proximity | Should proximity measure among the rows be calculated? |
| oob.prox | Should proximity be calculated only on “out-of-bag” data? |
| norm.votes | If TRUE (default), the final result of votes are expressed as fractions. If FALSE, raw vote counts are returned (useful for combining results from different runs). Ignored for regression. |
| do.trace | If set to TRUE, give a more verbose output as randomForest is run. If set to some integer, then running output is printed for every do.trace trees. |
| keep.forest | If set to FALSE, the forest will not be retained in the output object. If xtest is given, defaults to FALSE. |
| corr.bias | perform bias correction for regression? Note: Experimental. Use at your own risk. |
| keep.inbag | Should an n by ntree matrix be returned that keeps track of which samples are “in-bag” in which trees (but not how many times, if sampling with replacement) |
| ... | optional parameters to be passed to the low level functionrandomForest.default. |

**SL.randomforest() wrappers used in the SuperLearner library of the CBDA protocol**

SL.randomForest.1000 <- function(..., ntree=1000,family = "binomial"){

SL.randomForest(..., ntree = ntree,family = family)

}

SL.randomForest.500 <- function(..., ntree=500,family = "binomial"){

SL.randomForest(..., ntree = ntree,family = family)

}

SL.randomForest.300 <- function(..., ntree=300,family = "binomial"){

SL.randomForest(..., ntree = ntree,family = family)

}

SL.randomForest.100 <- function(..., ntree=100,family = "binomial"){

SL.randomForest(..., ntree = ntree,family = family)

}

SL.randomForest.50 <- function(..., ntree=50,family = "binomial"){

SL.randomForest(..., ntree = ntree,family = family)

}

SL.randomForest.20 <- function(..., ntree=20,family = "binomial"){

SL.randomForest(..., ntree = ntree,family = family)

}

## eXtreme Gradient Boosting Training – SL.xgboost

xgb.train(params = list(), data, nrounds, watchlist = list(), obj = NULL,

feval = NULL, verbose = 1, print_every_n = 1L,

early_stopping_rounds = NULL, maximize = NULL, save_period = NULL,

save_name = "xgboost.model", xgb_model = NULL, callbacks = list(), ...)

xgboost(data = NULL, label = NULL, missing = NA, weight = NULL,

params = list(), nrounds, verbose = 1, print_every_n = 1L,

early_stopping_rounds = NULL, maximize = NULL, save_period = NULL,

save_name = "xgboost.model", xgb_model = NULL, callbacks = list(), ...)

***Description***

| params | the list of parameters. The complete list of parameters is available at <http://xgboost.readthedocs.io/en/latest/parameter.html>. Below is a shorter summary:  1. General Parameters   - booster which booster to use, can be gbtree or gblinear. Default: gbtree.   2. Booster Parameters  2.1. Parameter for Tree Booster   - eta control the learning rate: scale the contribution of each tree by a factor of 0 < eta < 1 when it is added to the current approximation. Used to prevent overfitting by making the boosting process more conservative. Lower value for eta implies larger value for nrounds: low eta value means model more robust to overfitting but slower to compute. Default: 0.3 - gamma minimum loss reduction required to make a further partition on a leaf node of the tree. the larger, the more conservative the algorithm will be. - max_depth maximum depth of a tree. Default: 6 - min_child_weight minimum sum of instance weight (hessian) needed in a child. If the tree partition step results in a leaf node with the sum of instance weight less than min_child_weight, then the building process will give up further partitioning. In linear regression mode, this simply corresponds to minimum number of instances needed to be in each node. The larger, the more conservative the algorithm will be. Default: 1 - subsample subsample ratio of the training instance. Setting it to 0.5 means that xgboost randomly collected half of the data instances to grow trees and this will prevent overfitting. It makes computation shorter (because less data to analyze). It is advised to use this parameter with eta and increase nrounds. Default: 1 - colsample_bytree subsample ratio of columns when constructing each tree. Default: 1 - num_parallel_tree Experimental parameter. number of trees to grow per round. Useful to test Random Forest through Xgboost (set colsample_bytree < 1, subsample < 1 and round = 1) accordingly. Default: 1 - monotone_constraints A numerical vector consists of 1, 0 and -1 with its length equals to the number of features in the training data. 1 is increasing, -1 is decreasing and 0 is no constraint. - interaction_constraints A list of vectors specifying feature indices of permitted interactions. Each item of the list represents one permitted interaction where specified features are allowed to interact with each other. Feature index values should start from 0 (0 references the first column). Leave argument unspecified for no interaction constraints.   2.2. Parameter for Linear Booster   - lambda L2 regularization term on weights. Default: 0 - lambda_bias L2 regularization term on bias. Default: 0 - alpha L1 regularization term on weights. (there is no L1 reg on bias because it is not important). Default: 0   3. Task Parameters   - objective specify the learning task and the corresponding learning objective, users can pass a self-defined function to it. The default objective options are below:   - reg:squarederror Regression with squared loss (Default).   - reg:logistic logistic regression.   - binary:logistic logistic regression for binary classification. Output probability.   - binary:logitraw logistic regression for binary classification, output score before logistic transformation.   - num_class set the number of classes. To use only with multiclass objectives.   - multi:softmax set xgboost to do multiclass classification using the softmax objective. Class is represented by a number and should be from 0 to num_class - 1.   - multi:softprob same as softmax, but prediction outputs a vector of ndata * nclass elements, which can be further reshaped to ndata, nclass matrix. The result contains predicted probabilities of each data point belonging to each class.   - rank:pairwise set xgboost to do ranking task by minimizing the pairwise loss. - base_score the initial prediction score of all instances, global bias. Default: 0.5 - eval_metric evaluation metrics for validation data. Users can pass a self-defined function to it. Default: metric will be assigned according to objective (rmse for regression, and error for classification, mean average precision for ranking). List is provided in detail section. |
| --- | --- |
| data | training dataset. xgb.train accepts only an xgb.DMatrix as the input. xgboost, in addition, also accepts matrix, dgCMatrix, or name of a local data file. |
| nrounds | max number of boosting iterations. |
| watchlist | named list of xgb.DMatrix datasets to use for evaluating model performance. Metrics specified in either eval_metric or feval will be computed for each of these datasets during each boosting iteration, and stored in the end as a field named evaluation_log in the resulting object. When either verbose>=1 or [cb.print.evaluation](http://127.0.0.1:23359/help/library/xgboost/help/cb.print.evaluation) callback is engaged, the performance results are continuously printed out during the training. E.g., specifying watchlist=list(validation1=mat1, validation2=mat2) allows to track the performance of each round's model on mat1 and mat2. |
| obj | customized objective function. Returns gradient and second order gradient with given prediction and dtrain. |
| feval | customized evaluation function. Returnslist(metric='metric-name', value='metric-value') with given prediction and dtrain. |
| verbose | If 0, xgboost will stay silent. If 1, it will print information about performance. If 2, some additional information will be printed out. Note that setting verbose > 0 automatically engages the cb.print.evaluation(period=1) callback function. |
| print_every_n | Print each n-th iteration evaluation messages when verbose>0. Default is 1 which means all messages are printed. This parameter is passed to the [cb.print.evaluation](http://127.0.0.1:23359/help/library/xgboost/help/cb.print.evaluation) callback. |
| early_stopping_rounds | If NULL, the early stopping function is not triggered. If set to an integer k, training with a validation set will stop if the performance doesn't improve for k rounds. Setting this parameter engages the [cb.early.stop](http://127.0.0.1:23359/help/library/xgboost/help/cb.early.stop) callback. |
| maximize | If feval and early_stopping_rounds are set, then this parameter must be set as well. When it is TRUE, it means the larger the evaluation score the better. This parameter is passed to the [cb.early.stop](http://127.0.0.1:23359/help/library/xgboost/help/cb.early.stop) callback. |
| save_period | when it is non-NULL, model is saved to disk after every save_period rounds, 0 means save at the end. The saving is handled by the [cb.save.model](http://127.0.0.1:23359/help/library/xgboost/help/cb.save.model) callback. |
| save_name | the name or path for periodically saved model file. |
| xgb_model | a previously built model to continue the training from. Could be either an object of class xgb.Booster, or its raw data, or the name of a file with a previously saved model. |
| callbacks | a list of callback functions to perform various task during boosting. See [callbacks](http://127.0.0.1:23359/help/library/xgboost/help/callbacks). Some of the callbacks are automatically created depending on the parameters' values. User can provide either existing or their own callback methods in order to customize the training process. |
| ... | other parameters to pass to params. |
| label | vector of response values. Should not be provided when data is a local data file name or an xgb.DMatrix. |
| missing | by default is set to NA, which means that NA values should be considered as 'missing' by the algorithm. Sometimes, 0 or other extreme value might be used to represent missing values. This parameter is only used when input is a dense matrix. |
| weight | a vector indicating the weight for each row of the input. |

**SL.xgboost() wrappers used in the SuperLearner library of the CBDA protocol**

SL.xgboost.500 <- function(..., ntrees=500,family = "binomial"){

SL.xgboost(..., ntrees=500,family = family)

}

SL.xgboost.300 <- function(..., ntrees=300,family = "binomial"){

SL.xgboost(..., ntrees=300,family = family)

}

SL.xgboost.2000 <- function(..., ntrees=2000,family = "binomial"){

SL.xgboost(..., ntrees=2000,family = family)

}

SL.xgboost.100 <- function(..., ntrees=100,family = "binomial"){

SL.xgboost(..., ntrees=100,family = family)

}

SL.xgboost.1500 <- function(..., ntrees=1500,family = "binomial"){

SL.xgboost(..., ntrees=1500,family = family)

}

SL.xgboost.d3 <- function(..., max_depth=3, family = "binomial"){

SL.xgboost(..., max_depth=3, family = family)

}

SL.xgboost.d5 <- function(..., max_depth=5, family = "binomial"){

SL.xgboost(..., max_depth=3, family = family)

}

SL.xgboost.d6 <- function(..., max_depth=6, family = "binomial"){

SL.xgboost(..., max_depth=3, family = family)

}

SL.xgboost.gau <- function(..., family = "gaussian"){

SL.xgboost(..., family=family)

}

SL.xgboost.shrink.15 <- function(..., shrinkage=0.15, family="binomial"){

SL.xgboost(..., shrinkage=0.15, family=family)

}

SL.xgboost.shrink.2 <- function(..., shrinkage=0.2, family="binomial"){

SL.xgboost(..., shrinkage=0.2, family=family)

}

SL.xgboost.shrink.05 <- function(..., shrinkage=0.05, family="binomial"){

SL.xgboost(..., shrinkage=0.05, family=family)

}

SL.xgboost.shrink.25 <- function(..., shrinkage=0.25, family="binomial"){

SL.xgboost(..., shrinkage=0.25, family=family)

}

**bartMachine – SL.bartMachine()**

bartMachine(X = NULL, y = NULL, Xy = NULL,

num_trees = 50, num_burn_in = 250, num_iterations_after_burn_in = 1000,

alpha = 0.95, beta = 2, k = 2, q = 0.9, nu = 3, prob_rule_class = 0.5,

mh_prob_steps = c(2.5, 2.5, 4)/9,debug_log = FALSE,

run_in_sample = TRUE, s_sq_y = "mse",sig_sq_est = NULL,

cov_prior_vec = NULL, use_missing_data = FALSE,

covariates_to_permute = NULL, num_rand_samps_in_library = 10000,

use_missing_data_dummies_as_covars = FALSE,

replace_missing_data_with_x_j_bar = FALSE,

impute_missingness_with_rf_impute = FALSE,

impute_missingness_with_x_j_bar_for_lm = TRUE,

mem_cache_for_speed = TRUE,

serialize = FALSE,

seed = NULL,

verbose = TRUE)

***Description***

| X | Data frame of predictors. Factors are automatically converted to dummies internally. |
| --- | --- |
| y | Vector of response variable. If y is numeric or integer, a BART model for regression is built. If y is a factor with two levels, a BART model for classification is built. |
| Xy | A data frame of predictors and the response. The response column must be named “y”. |
| num_trees | The number of trees to be grown in the sum-of-trees model. |
| num_burn_in | Number of MCMC samples to be discarded as “burn-in”. |
| num_iterations_after_burn_in | Number of MCMC samples to draw from the posterior distribution of *\hat{f}(x)*. |
| alpha | Base hyperparameter in tree prior for whether a node is nonterminal or not. |
| beta | Power hyperparameter in tree prior for whether a node is nonterminal or not. |
| k | For regression, k determines the prior probability that *E(Y|X)* is contained in the interval *(y_{min}, y_{max})*, based on a normal distribution. For example, when *k=2*, the prior probability is 95%. For classification, k determines the prior probability that *E(Y|X)* is between *(-3,3)*. Note that a larger value of k results in more shrinkage and a more conservative fit. |
| q | Quantile of the prior on the error variance at which the data-based estimate is placed. Note that the larger the value of q, the more aggressive the fit as you are placing more prior weight on values lower than the data-based estimate. Not used for classification. |
| nu | Degrees of freedom for the inverse *χ^2* prior. Not used for classification. |
| prob_rule_class | Threshold for classification. Any observation with a conditional probability greater than prob_class_rule is assigned the “positive” outcome. Note that the first level of the response is treated as the “negative” outcome and the second is treated as the “positive” outcome. |
| mh_prob_steps | Vector of prior probabilities for proposing changes to the tree structures: (GROW, PRUNE, CHANGE) |
| debug_log | If TRUE, additional information about the model construction are printed to a file in the working directory. |
| run_in_sample | If TRUE, in-sample statistics such as *\hat{f}(x)*, Pseudo-*R^2*, and RMSE are computed. Setting this to FALSE when not needed can decrease computation time. |
| s_sq_y | If “mse”, a data-based estimated of the error variance is computed as the MSE from ordinary least squares regression. If “var”., the data-based estimate is computed as the variance of the response. Not used in classification. |
| sig_sq_est | Pass in an estimate of the maximum sig_sq of the model. This is useful to cache somewhere and then pass in during cross-validation since the default method of estimation is a linear model. In large dimensions, linear model estimation is slow. |
| cov_prior_vec | Vector assigning relative weights to how often a particular variable should be proposed as a candidate for a split. The vector is internally normalized so that the weights sum to 1. Note that the length of this vector must equal the length of the design matrix after dummification and augmentation of indicators of missingness (if used). To see what the dummified matrix looks like, use [dummify_data](http://127.0.0.1:23359/help/library/bartMachine/help/dummify_data). See Bleich et al. (2013) for more details on when this feature is most appropriate. |
| use_missing_data | If TRUE, the missing data feature is used to automatically handle missing data without imputation. See Kapelner and Bleich (2013) for details. |
| covariates_to_permute | Private argument for [cov_importance_test](http://127.0.0.1:23359/help/library/bartMachine/help/cov_importance_test). Not needed by user. |
| num_rand_samps_in_library | Before building a BART model, samples from the Standard Normal and *χ^2(ν)* are drawn to be used in the MCMC steps. This parameter determines the number of samples to be taken. |
| use_missing_data_dummies_as_covars | If TRUE, additional indicator variables for whether or not an observation in a particular column is missing are included. See Kapelner and Bleich (2013) for details. |
| replace_missing_data_with_x_j_bar | If TRUE ,missing entries in X are imputed with average value or modal category. |
| impute_missingness_with_rf_impute | If TRUE, missing entries are filled in using the rf.impute() function from the randomForest library. |
| impute_missingness_with_x_j_bar_for_lm | If TRUE, when computing the data-based estimate of *σ^2*, missing entries are imputed with average value or modal category. |
| mem_cache_for_speed | Speed enhancement that caches the predictors and the split values that are available at each node for selecting new rules. If the number of predictors is large, the memory requirements become large. We recommend keeping this on (default) and turning it off if you experience out-of-memory errors. |
| serialize | Setting this option to TRUE will allow serialization of bartMachine objects which allows for persistence between R sessions if the object is saved and reloaded. Note that serialized objects can take up a large amount of memory. Thus, the default is FALSE. |
| seed | Optional: sets the seed in both R and Java. Default is NULL which does not set the seed in R nor Java. |
| verbose | Prints information about progress of the algorithm to the screen. |

**SL.bartMachine() wrappers used in the SuperLearner library of the CBDA protocol**

SL.bartMachine.20 <- function(..., family = "binomial", ntrees = 20){

SL.bartMachine(..., family = family, ntrees = ntrees)

}

SL.bartMachine.100 <- function(..., family = "binomial", ntrees = 100){

SL.bartMachine(..., family = family, ntrees = ntrees)

}

SL.bartMachine.500 <- function(..., family = "binomial", ntrees = 500){

SL.bartMachine(..., family = family, ntrees = ntrees)

}

**glmnet – SL.glmnet()**

glmnet(x, y, family=c("gaussian","binomial","poisson","multinomial","cox","mgaussian"),

weights, offset=NULL, alpha = 1, nlambda = 100,

lambda.min.ratio = ifelse(nobs<nvars,0.01,0.0001), lambda=NULL,

standardize = TRUE, intercept=TRUE, thresh = 1e-07, dfmax = nvars + 1,

pmax = min(dfmax * 2+20, nvars), exclude, penalty.factor = rep(1, nvars),

lower.limits=-Inf, upper.limits=Inf, maxit=100000,

type.gaussian=ifelse(nvars<500,"covariance","naive"),

type.logistic=c("Newton","modified.Newton"),

standardize.response=FALSE, type.multinomial=c("ungrouped","grouped"))

***Description***

| x | input matrix, of dimension nobs x nvars; each row is an observation vector. Can be in sparse matrix format (inherit from class "sparseMatrix" as in package Matrix; not yet available for family="cox") |
| --- | --- |
| y | response variable. Quantitative for family="gaussian", or family="poisson" (non-negative counts). Forfamily="binomial" should be either a factor with two levels, or a two-column matrix of counts or proportions (the second column is treated as the target class; for a factor, the last level in alphabetical order is the target class). For family="multinomial", can be a nc>=2 level factor, or a matrix with nc columns of counts or proportions. For either "binomial" or "multinomial", if y is presented as a vector, it will be coerced into a factor. For family="cox", y should be a two-column matrix with columns named 'time' and 'status'. The latter is a binary variable, with '1' indicating death, and '0' indicating right censored. The function Surv() in package **survival** produces such a matrix. For family="mgaussian", y is a matrix of quantitative responses. |
| family | Response type (see above) |
| weights | observation weights. Can be total counts if responses are proportion matrices. Default is 1 for each observation |
| offset | A vector of length nobs that is included in the linear predictor (a nobs x nc matrix for the "multinomial" family). Useful for the "poisson" family (e.g. log of exposure time), or for refining a model by starting at a current fit. Default is NULL. If supplied, then values must also be supplied to the predict function. |
| alpha | The elasticnet mixing parameter, with *0≤α≤ 1*. The penalty is defined as  *(1-α)/2||β||_2^2+α||β||_1.*  alpha=1 is the lasso penalty, and alpha=0 the ridge penalty. |
| nlambda | The number of lambda values - default is 100. |
| lambda.min.ratio | Smallest value for lambda, as a fraction of lambda.max, the (data derived) entry value (i.e. the smallest value for which all coefficients are zero). The default depends on the sample size nobs relative to the number of variables nvars. If nobs > nvars, the default is 0.0001, close to zero. If nobs < nvars, the default is 0.01. A very small value oflambda.min.ratio will lead to a saturated fit in the nobs < nvars case. This is undefined for "binomial" and "multinomial" models, and glmnet will exit gracefully when the percentage deviance explained is almost 1. |
| lambda | A user supplied lambda sequence. Typical usage is to have the program compute its own lambda sequence based on lambda and lambda.min.ratio. Supplying a value of lambda overrides this. WARNING: use with care. Avoid supplying a single value for lambda (for predictions after CV use predict() instead). Supply instead a decreasing sequence of lambda values. glmnet relies on its warms starts for speed, and its often faster to fit a whole path than compute a single fit. |
| standardize | Logical flag for x variable standardization, prior to fitting the model sequence. The coefficients are always returned on the original scale. Default is standardize=TRUE. If variables are in the same units already, you might not wish to standardize. See details below for y standardization with family="gaussian". |
| intercept | Should intercept(s) be fitted (default=TRUE) or set to zero (FALSE) |
| thresh | Convergence threshold for coordinate descent. Each inner coordinate-descent loop continues until the maximum change in the objective after any coefficient update is less than thresh times the null deviance. Defaults value is 1E-7. |
| dfmax | Limit the maximum number of variables in the model. Useful for very large nvars, if a partial path is desired. |
| pmax | Limit the maximum number of variables ever to be nonzero |
| exclude | Indices of variables to be excluded from the model. Default is none. Equivalent to an infinite penalty factor (next item). |
| penalty.factor | Separate penalty factors can be applied to each coefficient. This is a number that multiplies lambda to allow differential shrinkage. Can be 0 for some variables, which implies no shrinkage, and that variable is always included in the model. Default is 1 for all variables (and implicitly infinity for variables listed in exclude). Note: the penalty factors are internally rescaled to sum to nvars, and the lambda sequence will reflect this change. |
| lower.limits | Vector of lower limits for each coefficient; default -Inf. Each of these must be non-positive. Can be presented as a single value (which will then be replicated), else a vector of length nvars |
| upper.limits | Vector of upper limits for each coefficient; default Inf. See lower.limits |
| maxit | Maximum number of passes over the data for all lambda values; default is 10^5. |
| type.gaussian | Two algorithm types are supported for (only)family="gaussian". The default when nvar<500 istype.gaussian="covariance", and saves all inner-products ever computed. This can be much faster thantype.gaussian="naive", which loops through nobsevery time an inner-product is computed. The latter can be far more efficient for nvar >> nobs situations, or when nvar > 500. |
| type.logistic | If "Newton" then the exact hessian is used (default), while "modified.Newton" uses an upper-bound on the hessian, and can be faster. |
| standardize.response | This is for the family="mgaussian" family, and allows the user to standardize the response variables |
| type.multinomial | If "grouped" then a grouped lasso penalty is used on the multinomial coefficients for a variable. This ensures they are all in our out together. The default is "ungrouped" |

**SL.glmnet() wrappers used in the SuperLearner library of the CBDA protocol**

SL.glmnet.0 <- function(..., alpha = 0,family="binomial"){

SL.glmnet(..., alpha = alpha , family = family)

}

SL.glmnet.0.75 <- function(..., alpha = 0.75,family="binomial"){

SL.glmnet(..., alpha = alpha , family = family)

}

SL.glmnet.0.25 <- function(..., alpha = 0.25,family="binomial"){

SL.glmnet(..., alpha = alpha, family = family)

}

SL.glmnet.0.50 <- function(..., alpha = 0.50,family="binomial"){

SL.glmnet(..., alpha = alpha, family = family)

}

**k-Nearest Neighbor – SL.knn**

knn(train, test, cl, k = 1, l = 0, prob = FALSE, use.all = TRUE)

***Description***

| train | matrix or data frame of training set cases. |
| --- | --- |
| test | matrix or data frame of test set cases. A vector will be interpreted as a row vector for a single case. |
| cl | factor of true classifications of training set |
| k | number of neighbors considered. |
| l | minimum vote for definite decision, otherwise doubt. (More precisely, less than k-l dissenting votes are allowed, even if k is increased by ties.) |
| prob | If this is true, the proportion of the votes for the winning class are returned as attribute prob. |
| use.all | controls handling of ties. If true, all distances equal to the kth largest are included. If false, a random selection of distances equal to the kth is chosen to use exactly k neighbors. |

**SL.knn() wrappers used in the SuperLearner library of the CBDA protocol**

SL.knn.5 <- function(..., k = 5,family = "binomial"){

SL.knn(..., k = k,family = family)

}

SL.knn.25 <- function(..., k = 25,family = "binomial"){

SL.knn(..., k = k,family = family)

}

SL.knn.50 <- function(..., k = 50,family = "binomial"){

SL.knn(..., k = k,family = family)

}

SL.knn.100 <- function(..., k = 100,family = "binomial"){

SL.knn(..., k = k,family = family)

}

## Supplementary Text S3: CBDA Convergence

This section outlines two alternative approaches for testing CBDA convergence and evaluate the overall performance on a generic dataset. Both approaches rely on the analysis of the ensemble predictor output. More specifically we first look at the overall distributions of the weights/coefficients that the ensemble predictor assigns to each of the machine learning and classification algorithms throughout the training stage of the CBDA protocol (see **Text S3.1** for details). The working hypothesis is that if there is sufficient energy, signal information, in the data, it will be reflected in some, at least a few, algorithms being consistently more predictive than others. The assumption behind each machine learning and classification algorithm is that there is a specific correlation structure in the data, and that we can exploit it for building better predictive models. For example, when we use logistic regression models, we assume a specific relationship (e.g., log-linear) between the output to be predicted and the features (covariates or regressors) available in the dataset. Other algorithms do not return explicit relationships between outcomes and features (i.e., support vector machine, neural network, random forest), however they still superimpose specific joint distributions on the data for the purpose of improving our predictions.

The second approach examines the similarity and variability among the ensemble predictor weights/coefficients across the top predictive models. Here the assumption is that higher similarity and lower variance can suggest the convergence of the CBDA protocol (see **Text S3.2** for details).

# *S3.1 Ensemble predictor’s weights distribution analysis*

The ensemble CBDA-SuperLearner model utilizes Non-Negative Least Squares (NNLS) to estimate the coefficients of a linear combination of predictive models:

$$\hat{f}^{SL}\left( \cdot\right)=\lambda_{1} f_{1}\left( \cdot\right)+\lambda_{2}f_{2}\left( \cdot\right)+...+\lambda_{s} f_{s}\left( \cdot\right), \sum_{j=1}^{s} \lambda_{j}=1 .$$

Each of the $\left\{ f_{j} \right\}_{j=1}^{s}$ is a base learner, e.g., weak learners available in the SL.library (see **Table 2** for the list of all the base learners used in the current CBDA implementation).

For each ensemble prediction model $\hat{f}^{SL}\left( \cdot\right)$, the weights/coefficients $\lambda_{j}$ are optimized to return the best ensemble prediction $F^{SL}(X,Y)$. In other words, the NNLS step weights the performance of each algorithm in the SuperLearner training/learning step and combines them in a way that the performance of $F^{SL}$ is as good as the best among the $f_{j}$. Note that some algorithms might perform better than others in certain “regions” of the Big Data and by combining them, we can improve the overall meta-algorithm performance. Early studies suggested some approaches for defining asymptotic and ergodic properties of ensemble predictors [13, 28, 53, 54].

If we assume that only one or very few statistical models (e.g., priors and likelihoods function assumptions) are the “true” assumptions/models explaining the data, then only a few $\lambda_{j}$ will be nonzero (or significantly non-trivial), or equivalently the set $\lambda_{j}\neq0$ , $j=1,2,\ldots,s$, will be sparse. If we populate the SL.library with a large enough classes of learning algorithms, then a sparse set $\lambda_{j}\neq0$ , $j=1,2,\ldots,s$, will very likely return a better prediction than a dense one.

We will investigate the relationship between the sparsity of $\left\{ \lambda_{j}\neq0 \right\}_{j=1}^{s}$ and the CBDA performance by generating enough empirical evidences to support our hypothesis. For example, we will compare the distributions of weights/coefficients between the studies on synthetic datasets generated without signal (e.g., Null datasets) and with signal, an ideal controlled scenario of white noise vs perfect information. We will then investigate the correlation between ensemble predictor accuracy or performance and the $\left\{ \lambda_{j} \right\}_{j=1}^{s}$ distributions using large biomedical data.

**Figures S2** and **S4** show some of the results of how this approach can shed some light into the overall performance of the CBDA protocol. A new function called *SLcoef_plot()* generates a barplot of the means of the SuperLearner coefficients for each algorithm in the SuperLearner library (across the M predictive model outputs) resulting from the CBDA training stage. Details on the *SLcoef_plot()* function can be found in the **Text S4** as well as in the help of the new package [19].

# *S3.2 Dissimilarity analysis of the ensemble predictor weights*

The convergence performance of ensemble predictors is typically assessed by evaluating the variance of the models predictions. For instance, in bagging, by simply calculating the variance of prediction results for each model, it is possible to assess whether the added model is useful or not. The classical strategy in bagging is described below,

$$f\left( x \right)=\sum_{\{\beta\in B\}} \lambda_{\beta} f^{\beta\left( x \right)}, e.g., f\left( x \right)=\frac{1}{B}\sum_{\{\beta\in B\}} f^{\beta\left( x \right)} .$$

If $\sum_{x} Var_{\beta} \left( f^{\beta\left( x \right)} \right)\leq\alpha$, for some $\alpha$, then the adding and averaging step are meaningful.

In CBDA, we use a similar strategy but the ensemble model predictions are now based on subsamples of the original Big Data. CBDA does not add models, but it ranks them based on two prediction performance metrics (i.e., Mean Squared Error-MSE and Accuracy). By evaluating the similarity and variability of the weights assigned to each algorithm of the ensemble predictor across the top-ranked predictive models, we mimic the bagging strategy of tracking the variance of the model predictions to ensure convergence.

# *CBDA Implementation:* After the initial steps of the CBDA protocol (random subsampling, training, ranking) of CBDA (see [4] for details), we obtain the models listed below (note: they've already been ranked by the accuracy/MSE):

$$\mathrm{SuperLearner}\left( \left[ X^{j},Y^{j} \right] \right)\to\hat{f}^{j}\left( \cdot\right), j=1,...,M.$$

Here $M$ is the total number of subsample cases, and $\hat{f}^{j}(\cdot)$ is a linear combination of the models trained by base learning algorithms in SuperLearner, which has the form of

$$\hat{f}^{j}\left( \cdot\right)=\lambda_{1}^{j} f_{1}^{j}\left( \cdot\right)+\lambda_{2}^{j} f_{2}^{j}\left( \cdot\right)+...+\lambda_{s}^{j} f_{s}^{j}\left( \cdot\right), j=1,...,M.$$

We denote the weights of the base learners as a vector equivalent to the weights matrix:

$$\Lambda^{M^{'}}=\left( \begin{matrix} \lambda_{1}^{1} & \cdots& \lambda_{s}^{1} \\ \vdots& \ddots& \vdots\\ \lambda_{1}^{M^{'}} & \cdots& \lambda_{s}^{M^{'}} \end{matrix} \right) .$$

Here $M^{'}$ is a changing parameter, which ranges from the total number of subsample experiments $M$, down to a small number. In detail, we design an algorithm calculating the change of similarity among coefficient matrix by using Bray-Curtis distance [46], described as $BC_{d}=\frac{\sum|x_{i}-x_{j} |}{\sum\left( x_{i}+x_{j} \right)}$. Ideally we want to see higher similarity with smaller values of $M^{'}.$ If the $BC_{d}$ does not make any significant change across the decreasing values of $M^{'}$, we can speculate that the performance of the CBDA protocol was not adequate. **Figure S2** shows some results of how this approach can guide our selection of the top predictive models at the end of the training stage of the CBDA protocol.

A new function called *BCplot()* generates Bray-Curtis index and variance trajectories of the M vectors of SuperLearner coefficients resulting from the CBDA training stage.

Details on the *BCplot()* function can be found in the **Text S4** as well as in the help() function of the new package [19].

## Supplementary Text S4: Description of some of the new CBDA 2.0 R package functions

**The CBDA_slicer function**

CBDA_slicer_real <- function(label_AUC=NULL,TotFeat=NULL,M=5000,

nonzero=NULL, top_list = c(50,100,500,1000,2000,3000,5000),

RData_to_load=NULL, alpha_list=c(9e-1,5e-1,1e-1,5e-2,1e-2,1e-3,1e-5,1e-8,1e-10,1e-13,1e-16,1e-20), RealCase=FALSE, TrueFeat=100)

The *CBDA_slicer()* function loads the RData workspace (very large) generated after the end of the training stage of the CBDA protocol and first saves a lighter version of it with only the necessary objects and parameters to perform several tasks.

The RData workspace is loaded based on the input *RData_to_load* and any output generated and saved by the function will be labeled with the *label_AUC* input. By default, the total number of subsamples *M* is set to 5000, and the values at which the top-ranked models M* are evaluated is listed in the *top_list* input. The *nonzero* input defines the true features for the synthetic datasets. By default, the function operates with synthetic datasets (input *RealCase*=FALSE). If *RealCase*=TRUE, the input nonzero is discarded and the input *TrueFeat* is used (by default set to 100). The input *TrueFeat* only indicates the number of possibly true features, not the exact true features (like the input *nonzero*). If *RealCase*=TRUE, and *TrueFeat* is set to NULL, the *TrueFeat* will be set to *3*log(TotFeat)*. For each of the *top_list* values, a bar plot with the frequency of each feature in the top $M^{*}$ subsamples is created, with horizontal lines based on the *alpha_list*. Each horizontal line value comes from the normal distribution of the feature densities resulting from $M^{*}=M$ (see **Figure 2** in the main manuscript for details). A Precision-Recall (PR) plot is also generated for each *top_list* value. The area under the curve (AUC) of each PR plot is calculated and then plotted in a final plot (AUC plot) that displays AUCs as a function of $M^{*}$ (see **Figure 3** in the main manuscript for details).

Due to the many plots created, they are not automatically generated and saved but only displayed in the R Studio viewer.

**The SLcoef_plot() function**

SLcoef_plot <- function(label_AUC=NULL,top_list = c(1000,5000),

workspace = NULL, RData_to_load=NULL,threshold=0.0)

The *SLcoef_plot()* function generates JPEG images (labeled with the *label_AUC* input) with the distributions of the ensemble predictor (SuperLearner) mean coefficients after the CBDA training stage is completed.

The function first loads the RData workspace specified in *RData_to_load* . By default, two plots are generated: one without enforcing any threshold (*threshold* input) for the values of the coefficients to display and one with the threshold parameter enforced. The input *workspace* is used as the location where to save the images. If not specified, the current working directory is used, and the images placed in the directory SLcoeff-plots, which must be created before running the script.

**The BCplot() function**

BCplot <- function(label_AUC=NULL,M=5000,

top_list = c(50,100,500,1000,2000,3000,5000), workspace = NULL, RData_to_load=NULL,lambda=0.02,binary=FALSE)

The *BCplot()* function generates two JPEG images (labeled with the *label_AUC* input) with (1) the Bray-Curtis dissimilarity index and (2) the variance of the SuperLearner coefficients distribution. The function first loads the RData workspace specified in *RData_to_load* . By default, two plots are generated where the x axis is represented by M* or *top_list* input. The input *workspace* is used as the location where to save the images. If not specified, the current working directory is used, and the images placed in the directory BC-Var-plots (which must be created before running the script). The input *lambda* (threshold for each coefficient to be counted as non-zero in the Bray-Curtis distances calculations) and *binary* (any coefficient above lambda is set to 1, the rest to 0) are used for the Bray-Curtis calculation and they are set to default values of 0.02 and FALSE, respectively.

**The Overfitting_plot() function**

Overfitting_plot <- function(label_AUC=NULL,

workspace = NULL,

RData_to_load=NULL)

The *Overfitting_plot* *()* function generates two JPEG images (labeled with the *label_AUC* input) with the Accuracy and MSE values returned by the ensemble predictor (SuperLearner) after the CBDA Overfitting Test stage is completed. Since the parameter max_cov in the CBDA workflow is usually set to 100, the *Overfitting_plot* *()* function generates plot with the x axis from 3 (i.e., min_cov parameter in the CBDA workflow, minimum number of features, namely the top 3, to include in the first model) to 100. The function first loads the RData workspace specified in *RData_to_load*. The input workspace is used as the location where to save the images.

## Supplementary Text S5: Data Wrangling of the UK Biobank dataset

**Complete physical features (with Field ID)**

"Sex", "Year of Birth:34", "Month of Birth:52", "Date of attending assessment centre 53.0", "Date of attending assessment centre 53.2", "UK Biobank assessment centre 54.0","Month of attending assessment centre 55.0","Month of attending assessment centre 55.2","Number of self-reported cancers 134","Number of self-reported non-cancer illnesses 135", "Date of consenting to join UK Biobank 200","Age when attended assessment centre 21003","Age at recruitment 21022","Genotype measurement batch 22000","Discrepancy b/t T1&standard-space(linearly) 25731","Discrepancy b/t T1&standard-space(nonlinearly) 25732","Amount of warping (non-linearly T1 to standard-space) 25733","Inverted signal-to-noise ratio in T1 25734", "Inverted contrast-to-noise ratio in T1 25735"

**Physical features with 2 levels (with Field ID)**

“Sex: 31”, “Test completion status: 4287”, ”Attempted Fluid intelligence FI test: 4924”, “Ever smoked: 20160”

**Physical features with 3 levels (with Field ID)**

"Number of times snap-button pressed 403","Nap during day 1190","Current tobacco smoking 1239", "Final attempt correct 4294","Brain MRI measuring method: 12187","Brain MRI measurement completed: 12188", "Prospective memory result: 20018"

**Physical features with 4 levels (with Field ID)**

"X134: Number of self-reported cancers " "X398: number of correct matches in round" "X403: Number of times snap-button pressed " "X1190: Nap during day " "X1200: sleeplessness/Insomnia" "X1210: snoring" "X1239: current tobacco smoking" "X1259:Smoking/Smokers in household" "X1920: Mood Swings" "X1930: Miserableness" "X1940: Irritability" "X1950: Sensitivity/Hurt feelings" "X1960: Fed-up feelings" , "X1970: Nervous feelings" "X1980: worrier/anxious feelings" "X1990: tense/highly strung" "X2000: worry too long after embarrassment" "X2010: suffer from nerves" "X2020: loneliness/isolation" "X2030: guilty feelings" "X2040: risk taking" "X2090: seen doctor for nerves, anxiety, tension or depression" "X2100 seen psychiatrist for nerves, anxiety, tension or depression " "X4292: PM initial answer" "X4293: PM final answer " "X4598: ever depressed for a whole week" , "X4631: Ever unenthusiastic/disinterested for a whole week " "X4642: ever manic/hyper for 2 days" "X4653: Ever highly irritable/argumentative for 2 days " "X20116" "X20117: alcohol drinker".

**Physical features with 5 levels (with Field ID,** excluding NA**)**

# "X134" "X398" "X403" "X1220" "X1249" "X1618" "X1628"

**Physical features with 6 levels (with Field ID,** excluding NA**)**

#"X403" "X1170" "X1180" "X2050" "X2060" "X2070" "X2080" "X4946" "X4968"

**Physical features with 7 levels (with Field ID,** excluding NA**)**

#"X1558" "X4935" "X4957" "X4979" "X4990" "X5001" "X20118"

**Physical features with 8 levels (with Field ID,** excluding NA**)**

#"X403" "X4526" "X4548" "X4559" "X4570" "X4581" "X6138" "X6145"

**Physical features with 9 levels (with Field ID,** excluding NA**)**

#"X4537"

**Physical features with 10 levels (with Field ID,** excluding NA**)**

#"X401" "X402" "X403"

**Field ID and descriptions of the features that were eliminated from the analysis for being redundant or very similar to the outcome to be predicted, namely “Irritability”**

| Field ID | Description | Correlation to Irritability |
| --- | --- | --- |
| X1940.0.0 | Irritability | 1 |
| X20127.0.0 | neuroticism score | 0.54 |
| X1940.2.0 | Irritability (2^nd^ time point) | 0.49 |
| X1920.0.0 | Mood Swings | 0.38 |
| X1960.0.0 | Fed-up feelings | 0.36 |
| X1990.0.0 | Tense/highly strung | 0.33 |
| X1920.2.0 | Mood Swings (2^nd^ time point) | 0.29 |
| X1930.0.0 | Miserableness | 0.29 |
| X1960.2.0 | Fed-up feelings (2^nd^ time point) | 0.27 |
| X1990.2.0 | Tense/highly strung (2^nd^ time point) | 0.25 |

## Supplementary Table S1

## Top 100 features resulting from the CBDA analysis on the UK Biobank with neuroimaging biomarkers only (Figure S5-A)

| **Ranking** | **Atlas** | **Description** |
| --- | --- | --- |
| 1 | lh_aparc.a2009s_thickness | lh_G_cingul.Post.ventral_thickness |
| 2 | lh_aparc.DKTatlas_volume | lh_insula_volume |
| 3 | rh_aparc.DKTatlas_area | rh_precentral_area |
| 4 | lh_aparc_volume | lh_inferiorparietal_volume |
| 5 | lh_aparc.a2009s_foldind | lh_S_intrapariet.P_trans_foldind |
| 6 | lh_aparc.a2009s_gauscurv | lh_Lat_Fis.ant.Vertical_gauscurv |
| 7 | lh_aparc.a2009s_thickness | lh_S_collat_transv_ant_thickness |
| 8 | lh_aparc.DKTatlas_thickness | lh_rostralmiddlefrontal_thickness |
| 9 | lh_aparc.DKTatlas_volume | lh_pericalcarine_volume |
| 10 | lh_BA_exvivo.thresh_thickness | lh_BA4a_exvivo_thickness |
| 11 | rh_aparc.a2009s_volume | rh_S_calcarine_volume |
| 12 | rh_aparc.DKTatlas_gauscurv | rh_precuneus_gauscurv |
| 13 | rh_aparc.DKTatlas_thickness | rh_rostralmiddlefrontal_thickness |
| 14 | rh_aparc.DKTatlas_thicknessstd | rh_superiortemporal_thicknessstd |
| 15 | rh_BA_exvivo_thicknessstd | rh_BA44_exvivo_thicknessstd |
| 16 | rh_BA_exvivo_volume | rh_V1_exvivo_volume |
| 17 | rh_BA_exvivo.thresh_thickness | rh_BA45_exvivo_thickness |
| 18 | lh.w.g.pct.mean | insula |
| 19 | lh.w.g.pct.std | bankssts |
| 20 | lh_aparc_meancurv | lh_middletemporal_meancurv |
| 21 | lh_aparc_thickness | lh_posteriorcingulate_thickness |
| 22 | lh_aparc_thickness | lh_rostralmiddlefrontal_thickness |
| 23 | lh_aparc_thickness | lh_insula_thickness |
| 24 | lh_aparc_thicknessstd | lh_cuneus_thicknessstd |
| 25 | lh_aparc_thicknessstd | lh_inferiorparietal_thicknessstd |
| 26 | lh_aparc.a2009s_area | lh_G_parietal_sup_area |
| 27 | lh_aparc.a2009s_curvind | lh_S_pericallosal_curvind |
| 28 | lh_aparc.a2009s_foldind | lh_G_temp_sup.G_T_transv_foldind |
| 29 | lh_aparc.a2009s_foldind | lh_S_precentral.inf.part_foldind |
| 30 | lh_aparc.a2009s_gauscurv | lh_G_oc.temp_med.Lingual_gauscurv |
| 31 | lh_aparc.a2009s_meancurv | lh_G.S_frontomargin_meancurv |
| 32 | lh_aparc.a2009s_meancurv | lh_S_interm_prim.Jensen_meancurv |
| 33 | lh_aparc.a2009s_thickness | lh_S_oc_middle.Lunatus_thickness |
| 34 | lh_aparc.a2009s_thickness | lh_S_orbital_med.olfact_thickness |
| 35 | lh_aparc.a2009s_thicknessstd | lh_G_rectus_thicknessstd |
| 36 | lh_aparc.a2009s_thicknessstd | lh_S_circular_insula_ant_thicknessstd |
| 37 | lh_aparc.a2009s_thicknessstd | lh_S_circular_insula_inf_thicknessstd |
| 38 | lh_aparc.a2009s_volume | lh_G.S_occipital_inf_volume |
| 39 | lh_aparc.a2009s_volume | lh_S_suborbital_volume |
| 40 | lh_aparc.pial_meancurv | lh_precentral_meancurv |
| 41 | lh_BA_exvivo_meancurv | lh_BA6_exvivo_meancurv |
| 42 | lh_BA_exvivo.thresh_gauscurv | lh_entorhinal_exvivo_gauscurv |
| 43 | lh_BA_exvivo.thresh_volume | lh_BA6_exvivo_volume |
| 44 | rh_aparc_area | rh_postcentral_area |
| 45 | rh_aparc_meancurv | rh_lateraloccipital_meancurv |
| 46 | rh_aparc_thickness | rh_paracentral_thickness |
| 47 | rh_aparc_thicknessstd | rh_lateraloccipital_thicknessstd |
| 48 | rh_aparc_thicknessstd | rh_pericalcarine_thicknessstd |
| 49 | rh_aparc.a2009s_curvind | rh_G_front_inf.Orbital_curvind |
| 50 | rh_aparc.a2009s_curvind | rh_S_temporal_transverse_curvind |
| 51 | rh_aparc.a2009s_foldind | rh_G_front_inf.Opercular_foldind |
| 52 | rh_aparc.a2009s_gauscurv | rh_G.S_cingul.Ant_gauscurv |
| 53 | rh_aparc.a2009s_gauscurv | rh_G_front_inf.Triangul_gauscurv |
| 54 | rh_aparc.a2009s_gauscurv | rh_G_insular_short_gauscurv |
| 55 | rh_aparc.a2009s_gauscurv | rh_S_intrapariet.P_trans_gauscurv |
| 56 | rh_aparc.a2009s_meancurv | rh_Lat_Fis.ant.Vertical_meancurv |
| 57 | rh_aparc.a2009s_meancurv | rh_S_circular_insula_inf_meancurv |
| 58 | rh_aparc.a2009s_meancurv | rh_S_occipital_ant_meancurv |
| 59 | rh_aparc.a2009s_thickness | rh_G_occipital_sup_thickness |
| 60 | rh_aparc.a2009s_thickness | rh_S_collat_transv_post_thickness |
| 61 | rh_aparc.a2009s_thicknessstd | rh_G_occipital_middle_thicknessstd |
| 62 | rh_aparc.a2009s_volume | rh_S_front_inf_volume |
| 63 | rh_aparc.DKTatlas_meancurv | rh_middletemporal_meancurv |
| 64 | rh_aparc.DKTatlas_thickness | rh_entorhinal_thickness |
| 65 | rh_aparc.DKTatlas_thickness | rh_transversetemporal_thickness |
| 66 | rh_aparc.DKTatlas_volume | rh_precentral_volume |
| 67 | rh_aparc.pial_area | rh_transversetemporal_area |
| 68 | rh_aparc.pial_curvind | rh_parstriangularis_curvind |
| 69 | rh_aparc.pial_gauscurv | rh_precuneus_gauscurv |
| 70 | rh_BA_exvivo_volume | rh_entorhinal_exvivo_volume |
| 71 | rh_BA_exvivo.thresh_gauscurv | rh_BA4p_exvivo_gauscurv |
| 72 | lh.w.g.pct.std | frontalpole |
| 73 | rh.w.g.pct.mean | parsopercularis |
| 74 | rh.w.g.pct.std | pericalcarine |
| 75 | lh_aparc_area | lh_medialorbitofrontal_area |
| 76 | lh_aparc_area | lh_parahippocampal_area |
| 77 | lh_aparc_area | lh_paracentral_area |
| 78 | lh_aparc_curvind | lh_lingual_curvind |
| 79 | lh_aparc_curvind | lh_medialorbitofrontal_curvind |
| 80 | lh_aparc_curvind | lh_parsopercularis_curvind |
| 81 | lh_aparc_foldind | lh_lingual_foldind |
| 82 | lh_aparc_foldind | lh_pericalcarine_foldind |
| 83 | lh_aparc_gauscurv | lh_bankssts_gauscurv |
| 84 | lh_aparc_gauscurv | lh_temporalpole_gauscurv |
| 85 | lh_aparc_meancurv | lh_rostralmiddlefrontal_meancurv |
| 86 | lh_aparc_meancurv | lh_superiorparietal_meancurv |
| 87 | lh_aparc_thicknessstd | lh_posteriorcingulate_thicknessstd |
| 88 | lh_aparc_volume | lh_parahippocampal_volume |
| 89 | lh_aparc_volume | lh_posteriorcingulate_volume |
| 90 | lh_aparc_volume | lh_rostralanteriorcingulate_volume |
| 91 | lh_aparc.a2009s_area | lh_G_temp_sup.Plan_tempo_area |
| 92 | lh_aparc.a2009s_area | lh_S_parieto_occipital_area |
| 93 | lh_aparc.a2009s_curvind | lh_G_precuneus_curvind |
| 94 | lh_aparc.a2009s_curvind | lh_G_temporal_middle_curvind |
| 95 | lh_aparc.a2009s_curvind | lh_S_circular_insula_ant_curvind |
| 96 | lh_aparc.a2009s_curvind | lh_S_front_sup_curvind |
| 97 | lh_aparc.a2009s_curvind | lh_S_intrapariet.P_trans_curvind |
| 98 | lh_aparc.a2009s_curvind | lh_S_postcentral_curvind |
| 99 | lh_aparc.a2009s_foldind | lh_G.S_transv_frontopol_foldind |
| 100 | lh_aparc.a2009s_foldind | lh_G_front_sup_foldind |

## Supplementary Table S2

## Top 100 features resulting from the CBDA analysis on the UK Biobank with neuroimaging biomarkers and physical features (Figure 6A)

**Grey highlights: clinical features**

| **Ranking** | **ID** | **Description or Field ID** |
| --- | --- | --- |
| 1 | lh_aparc.a2009s_curvind | lh_Pole_temporal_curvind |
| 2 | rh_aparc.DKTatlas_curvind | rh_supramarginal_curvind |
| 3 | lh_aparc_thicknessstd | lh_rostralmiddlefrontal_thicknessstd |
| 4 | lh_aparc.a2009s_foldind | lh_G_temporal_middle_foldind |
| 5 | lh_aparc.a2009s_meancurv | lh_G_cingul.Post.dorsal_meancurv |
| 6 | lh_aparc.a2009s_meancurv | lh_S_calcarine_meancurv |
| 7 | lh_aparc.DKTatlas_volume | lh_isthmuscingulate_volume |
| 8 | rh_aparc_gauscurv | rh_lateralorbitofrontal_gauscurv |
| 9 | rh_aparc_thicknessstd | rh_isthmuscingulate_thicknessstd |
| 10 | rh_aparc.a2009s_area | rh_G_temporal_inf_area |
| 11 | rh_aparc.a2009s_gauscurv | rh_G_parietal_sup_gauscurv |
| 12 | rh_aparc.a2009s_gauscurv | rh_S_central_gauscurv |
| 13 | rh_aparc.a2009s_thicknessstd | rh_G_oc.temp_lat.fusifor_thicknessstd |
| 14 | rh_aparc.pial_curvind | rh_posteriorcingulate_curvind |
| 15 | rh_aparc.pial_gauscurv | rh_caudalanteriorcingulate_gauscurv |
| 16 | rh_BA_exvivo_thickness | rh_BA45_exvivo_thickness |
| 17 | rh_BA_exvivo_thickness | rh_V2_exvivo_thickness |
| 18 | rh_BA_exvivo.thresh_meancurv | rh_BA4p_exvivo_meancurv |
| 19 | rh_BA_exvivo.thresh_volume | rh_BA1_exvivo_volume |
| 20 | X25062.2.0 = [Mean FA in corticospinal tract on FA skeleton (right)](http://biobank.ctsu.ox.ac.uk/crystal/field.cgi?id=25062) | |
| 21 | X25285.2.0 = Mean L2 in cingulum hippocampus on FA skeleton (left) | |
| 22 | X25324.2.0 = Mean L3 in posterior thalamic radiation on FA skeleton (right) | |
| 23 | X25541.2.0 = Weighted-mean MD in tract uncinate fasciculus (right) | |
| 24 | X25701.2.0 = Weighted-mean OD in tract superior thalamic radiation (right) | |
| 25 | lh_aparc_curvind | lh_caudalmiddlefrontal_curvind |
| 26 | lh_aparc_curvind | lh_temporalpole_curvind |
| 27 | lh_aparc_foldind | lh_inferiorparietal_foldind |
| 28 | lh_aparc_gauscurv | lh_inferiorparietal_gauscurv |
| 29 | lh_aparc_meancurv | lh_transversetemporal_meancurv |
| 30 | lh_aparc_thickness | lh_bankssts_thickness |
| 31 | lh_aparc_thickness | lh_medialorbitofrontal_thickness |
| 32 | lh_aparc_volume | lh_postcentral_volume |
| 33 | lh_aparc_volume | lh_superiorparietal_volume |
| 34 | lh_aparc.a2009s_area | lh_G_pariet_inf.Supramar_area |
| 35 | lh_aparc.a2009s_area | lh_G_temporal_inf_area |
| 36 | lh_aparc.a2009s_area | lh_S_oc.temp_lat_area |
| 37 | lh_aparc.a2009s_curvind | lh_G_front_inf.Orbital_curvind |
| 38 | lh_aparc.a2009s_curvind | lh_G_front_middle_curvind |
| 39 | lh_aparc.a2009s_curvind | lh_S_circular_insula_ant_curvind |
| 40 | lh_aparc.a2009s_gauscurv | lh_G_front_inf.Opercular_gauscurv |
| 41 | lh_aparc.a2009s_gauscurv | lh_G_pariet_inf.Angular_gauscurv |
| 42 | lh_aparc.a2009s_meancurv | lh_G.S_transv_frontopol_meancurv |
| 43 | lh_aparc.a2009s_meancurv | lh_G_pariet_inf.Angular_meancurv |
| 44 | lh_aparc.a2009s_meancurv | lh_S_central_meancurv |
| 45 | lh_aparc.a2009s_meancurv | lh_S_precentral.sup.part_meancurv |
| 46 | lh_aparc.a2009s_thicknessstd | lh_G_temporal_inf_thicknessstd |
| 47 | lh_aparc.a2009s_thicknessstd | lh_Lat_Fis.ant.Vertical_thicknessstd |
| 48 | lh_aparc.a2009s_thicknessstd | lh_Pole_occipital_thicknessstd |
| 49 | lh_aparc.a2009s_volume | lh_G_pariet_inf.Angular_volume |
| 50 | lh_aparc.a2009s_volume | lh_G_temp_sup.Lateral_volume |
| 51 | lh_aparc.DKTatlas_area | lh_lateraloccipital_area |
| 52 | lh_aparc.DKTatlas_curvind | lh_superiortemporal_curvind |
| 53 | lh_aparc.DKTatlas_foldind | lh_postcentral_foldind |
| 54 | lh_aparc.DKTatlas_thickness | lh_precentral_thickness |
| 55 | lh_aparc.pial_gauscurv | lh_precentral_gauscurv |
| 56 | lh_aparc.pial_gauscurv | lh_temporalpole_gauscurv |
| 57 | lh_aparc.pial_meancurv | lh_paracentral_meancurv |
| 58 | lh_BA_exvivo_area | lh_perirhinal_exvivo_area |
| 59 | lh_BA_exvivo_foldind | lh_BA3a_exvivo_foldind |
| 60 | lh_BA_exvivo.thresh_gauscurv | lh_BA3b_exvivo_gauscurv |
| 61 | lh_BA_exvivo.thresh_meancurv | lh_BA45_exvivo_meancurv |
| 62 | lh_BA_exvivo.thresh_thickness | lh_MT_exvivo_thickness |
| 63 | lh_BA_exvivo.thresh_thicknessstd | lh_BA4p_exvivo_thicknessstd |
| 64 | lh_BA_exvivo.thresh_thicknessstd | lh_BA6_exvivo_thicknessstd |
| 65 | rh_aparc_area | rh_superiorparietal_area |
| 66 | rh_aparc_curvind | rh_parsorbitalis_curvind |
| 67 | rh_aparc_curvind | rh_temporalpole_curvind |
| 68 | rh_aparc_foldind | rh_pericalcarine_foldind |
| 69 | rh_aparc_meancurv | rh_middletemporal_meancurv |
| 70 | rh_aparc_meancurv | rh_parahippocampal_meancurv |
| 71 | rh_aparc_meancurv | rh_precuneus_meancurv |
| 72 | rh_aparc_thickness | rh_insula_thickness |
| 73 | rh_aparc_thicknessstd | rh_inferiorparietal_thicknessstd |
| 74 | rh_aparc.a2009s_area | rh_G_cuneus_area |
| 75 | rh_aparc.a2009s_area | rh_G_front_inf.Triangul_area |
| 76 | rh_aparc.a2009s_area | rh_G_insular_short_area |
| 77 | rh_aparc.a2009s_curvind | rh_G_temp_sup.Lateral_curvind |
| 78 | rh_aparc.a2009s_curvind | rh_Pole_temporal_curvind |
| 79 | rh_aparc.a2009s_foldind | rh_G.S_cingul.Mid.Ant_foldind |
| 80 | rh_aparc.a2009s_foldind | rh_S_precentral.inf.part_foldind |
| 81 | rh_aparc.a2009s_foldind | rh_S_temporal_inf_foldind |
| 82 | rh_aparc.a2009s_gauscurv | rh_G_front_inf.Orbital_gauscurv |
| 83 | rh_aparc.a2009s_gauscurv | rh_G_temp_sup.Plan_tempo_gauscurv |
| 84 | rh_aparc.a2009s_gauscurv | rh_S_temporal_transverse_gauscurv |
| 85 | rh_aparc.a2009s_meancurv | rh_S_oc_middle.Lunatus_meancurv |
| 86 | rh_aparc.a2009s_meancurv | rh_S_parieto_occipital_meancurv |
| 87 | rh_aparc.a2009s_thickness | rh_G_front_inf.Opercular_thickness |
| 88 | rh_aparc.a2009s_thickness | rh_G_temp_sup.Plan_polar_thickness |
| 89 | rh_aparc.a2009s_thickness | rh_Pole_temporal_thickness |
| 90 | rh_aparc.a2009s_thickness | rh_S_intrapariet.P_trans_thickness |
| 91 | rh_aparc.a2009s_thicknessstd | rh_G_oc.temp_med.Lingual_thicknessstd |
| 92 | rh_aparc.a2009s_thicknessstd | rh_G_rectus_thicknessstd |
| 93 | rh_aparc.a2009s_thicknessstd | rh_S_temporal_inf_thicknessstd |
| 94 | rh_aparc.a2009s_volume | rh_G_oc.temp_med.Lingual_volume |
| 95 | rh_aparc.DKTatlas_area | rh_caudalanteriorcingulate_area |
| 96 | rh_aparc.DKTatlas_area | rh_superiorfrontal_area |
| 97 | rh_aparc.DKTatlas_meancurv | rh_rostralanteriorcingulate_meancurv |
| 98 | rh_aparc.DKTatlas_thicknessstd | rh_medialorbitofrontal_thicknessstd |
| 99 | rh_aparc.pial_area | rh_posteriorcingulate_area |
| 100 | rh_aparc.pial_area | rh_superiortemporal_area |

## Supplementary Table S3

**Top 300 features selected from the CBDA analysis on the UK Biobank** **with neuroimaging biomarkers only (Figure S5-B)**

**Green highlights: overlap with Table S4**

| **Rank** | **Atlas** | **Description** |
| --- | --- | --- |
| 1 | lh_aparc_thicknessstd | lh_supramarginal_thicknessstd |
| 2 | lh_aparc.a2009s_gauscurv | lh_G_oc.temp_lat.fusifor_gauscurv |
| 3 | rh_aparc.a2009s_volume | rh_S_cingul.Marginalis_volume |
| 4 | lh_aparc.pial_meancurv | lh_frontalpole_meancurv |
| 5 | lh_BA_exvivo.thresh_meancurv | lh_BA2_exvivo_meancurv |
| 6 | rh_aparc_meancurv | rh_parstriangularis_meancurv |
| 7 | rh_aparc_thickness | rh_transversetemporal_thickness |
| 8 | rh_BA_exvivo.thresh_foldind | rh_BA4p_exvivo_foldind |
| 9 | rh_BA_exvivo.thresh_meancurv | rh_BA4p_exvivo_meancurv |
| 10 | lh_aparc_gauscurv | lh_pericalcarine_gauscurv |
| 11 | lh_aparc.a2009s_area | lh_G_occipital_middle_area |
| 12 | lh_aparc.a2009s_meancurv | lh_S_precentral.inf.part_meancurv |
| 13 | lh_aparc.a2009s_thickness | lh_G_precentral_thickness |
| 14 | lh_aparc.a2009s_thickness | lh_G_temp_sup.G_T_transv_thickness |
| 15 | lh_aparc.a2009s_thickness | lh_S_collat_transv_ant_thickness |
| 16 | lh_aparc.a2009s_thicknessstd | lh_S_suborbital_thicknessstd |
| 17 | lh_aparc.a2009s_volume | lh_G_occipital_sup_volume |
| 18 | lh_aparc.DKTatlas_thicknessstd | lh_fusiform_thicknessstd |
| 19 | lh_aparc.pial_curvind | lh_inferiortemporal_curvind |
| 20 | lh_aparc.pial_curvind | lh_isthmuscingulate_curvind |
| 21 | lh_aparc.pial_curvind | lh_lateraloccipital_curvind |
| 22 | rh_aparc.a2009s_foldind | rh_G_temp_sup.G_T_transv_foldind |
| 23 | rh_aparc.a2009s_gauscurv | rh_G_occipital_sup_gauscurv |
| 24 | rh_aparc.a2009s_thicknessstd | rh_G_cingul.Post.ventral_thicknessstd |
| 25 | rh_aparc.DKTatlas_gauscurv | rh_paracentral_gauscurv |
| 26 | rh_aparc.DKTatlas_meancurv | rh_parsorbitalis_meancurv |
| 27 | lh_aparc_foldind | lh_parahippocampal_foldind |
| 28 | lh_aparc.a2009s_area | lh_S_orbital_med.olfact_area |
| 29 | lh_aparc.a2009s_area | lh_WhiteSurfArea_area |
| 30 | lh_aparc.a2009s_curvind | lh_G.S_subcentral_curvind |
| 31 | lh_aparc.a2009s_curvind | lh_G_postcentral_curvind |
| 32 | lh_aparc.a2009s_foldind | lh_Lat_Fis.post_foldind |
| 33 | lh_aparc.a2009s_foldind | lh_S_suborbital_foldind |
| 34 | lh_aparc.a2009s_meancurv | lh_G_precentral_meancurv |
| 35 | lh_aparc.a2009s_volume | lh_S_collat_transv_post_volume |
| 36 | lh_aparc.DKTatlas_area | lh_rostralmiddlefrontal_area |
| 37 | lh_aparc.DKTatlas_gauscurv | lh_lingual_gauscurv |
| 38 | lh_aparc.DKTatlas_gauscurv | lh_transversetemporal_gauscurv |
| 39 | lh_aparc.DKTatlas_thicknessstd | lh_isthmuscingulate_thicknessstd |
| 40 | lh_aparc.pial_foldind | lh_caudalanteriorcingulate_foldind |
| 41 | lh_aparc.pial_meancurv | lh_temporalpole_meancurv |
| 42 | rh_aparc_gauscurv | rh_pericalcarine_gauscurv |
| 43 | rh_aparc_meancurv | rh_bankssts_meancurv |
| 44 | rh_aparc_meancurv | rh_parsopercularis_meancurv |
| 45 | rh_aparc_thickness | rh_parahippocampal_thickness |
| 46 | rh_aparc.a2009s_area | rh_G.S_subcentral_area |
| 47 | rh_aparc.a2009s_area | rh_G.S_cingul.Mid.Post_area |
| 48 | rh_aparc.a2009s_curvind | rh_S_intrapariet.P_trans_curvind |
| 49 | rh_aparc.a2009s_curvind | rh_S_suborbital_curvind |
| 50 | rh_aparc.a2009s_foldind | rh_G_orbital_foldind |
| 51 | rh_aparc.a2009s_gauscurv | rh_S_circular_insula_inf_gauscurv |
| 52 | rh_aparc.a2009s_thickness | rh_G.S_cingul.Mid.Ant_thickness |
| 53 | rh_aparc.DKTatlas_meancurv | rh_parahippocampal_meancurv |
| 54 | rh_aparc.pial_area | rh_entorhinal_area |
| 55 | rh_BA_exvivo.thresh_volume | rh_perirhinal_exvivo_volume |
| 56 | aseg | MaskVol.to.eTIV |
| 57 | lh.w.g.pct.std | cuneus |
| 58 | lh_aparc_area | lh_middletemporal_area |
| 59 | lh_aparc_area | lh_parahippocampal_area |
| 60 | lh_aparc_area | lh_parsopercularis_area |
| 61 | lh_aparc_foldind | lh_caudalmiddlefrontal_foldind |
| 62 | lh_aparc_foldind | lh_lateraloccipital_foldind |
| 63 | lh_aparc_gauscurv | lh_insula_gauscurv |
| 64 | lh_aparc_meancurv | lh_posteriorcingulate_meancurv |
| 65 | lh_aparc_thickness | lh_fusiform_thickness |
| 66 | lh_aparc_thicknessstd | lh_bankssts_thicknessstd |
| 67 | lh_aparc_volume | lh_parsopercularis_volume |
| 68 | lh_aparc.a2009s_curvind | lh_G_oc.temp_med.Parahip_curvind |
| 69 | lh_aparc.a2009s_curvind | lh_S_front_middle_curvind |
| 70 | lh_aparc.a2009s_curvind | lh_S_oc_middle.Lunatus_curvind |
| 71 | lh_aparc.a2009s_curvind | lh_S_oc.temp_med.Lingual_curvind |
| 72 | lh_aparc.a2009s_gauscurv | lh_G_occipital_middle_gauscurv |
| 73 | lh_aparc.a2009s_gauscurv | lh_S_temporal_inf_gauscurv |
| 74 | lh_aparc.a2009s_meancurv | lh_G_front_inf.Orbital_meancurv |
| 75 | lh_aparc.a2009s_thickness | lh_G_temporal_middle_thickness |
| 76 | lh_aparc.a2009s_thicknessstd | lh_G.S_subcentral_thicknessstd |
| 77 | lh_aparc.DKTatlas_gauscurv | lh_rostralanteriorcingulate_gauscurv |
| 78 | lh_aparc.DKTatlas_meancurv | lh_precuneus_meancurv |
| 79 | lh_aparc.pial_curvind | lh_bankssts_curvind |
| 80 | lh_aparc.pial_gauscurv | lh_cuneus_gauscurv |
| 81 | lh_aparc.pial_gauscurv | lh_fusiform_gauscurv |
| 82 | lh_aparc.pial_gauscurv | lh_isthmuscingulate_gauscurv |
| 83 | lh_aparc.pial_gauscurv | lh_lateralorbitofrontal_gauscurv |
| 84 | lh_aparc.pial_meancurv | lh_lateralorbitofrontal_meancurv |
| 85 | lh_aparc.pial_meancurv | lh_superiorfrontal_meancurv |
| 86 | lh_BA_exvivo_area | lh_perirhinal_exvivo_area |
| 87 | lh_BA_exvivo_curvind | lh_BA44_exvivo_curvind |
| 88 | lh_BA_exvivo_foldind | lh_entorhinal_exvivo_foldind |
| 89 | lh_BA_exvivo.thresh_area | lh_BA6_exvivo_area |
| 90 | lh_BA_exvivo.thresh_curvind | lh_V1_exvivo_curvind |
| 91 | lh_BA_exvivo.thresh_meancurv | lh_BA1_exvivo_meancurv |
| 92 | lh_BA_exvivo.thresh_thickness | lh_entorhinal_exvivo_thickness |
| 93 | lh_BA_exvivo.thresh_thicknessstd | lh_BA4p_exvivo_thicknessstd |
| 94 | lh_BA_exvivo.thresh_volume | lh_BA4a_exvivo_volume |
| 95 | rh_aparc_area | rh_medialorbitofrontal_area |
| 96 | rh_aparc_area | rh_insula_area |
| 97 | rh_aparc_curvind | rh_isthmuscingulate_curvind |
| 98 | rh_aparc_curvind | rh_posteriorcingulate_curvind |
| 99 | rh_aparc_gauscurv | rh_rostralmiddlefrontal_gauscurv |
| 100 | rh_aparc_thickness | rh_frontalpole_thickness |
| 101 | rh_aparc_thicknessstd | rh_lateraloccipital_thicknessstd |
| 102 | rh_aparc.a2009s_area | rh_Lat_Fis.ant.Horizont_area |
| 103 | rh_aparc.a2009s_curvind | rh_S_interm_prim.Jensen_curvind |
| 104 | rh_aparc.a2009s_curvind | rh_S_orbital.H_Shaped_curvind |
| 105 | rh_aparc.a2009s_foldind | rh_G_insular_short_foldind |
| 106 | rh_aparc.a2009s_gauscurv | rh_G_postcentral_gauscurv |
| 107 | rh_aparc.a2009s_meancurv | rh_S_front_inf_meancurv |
| 108 | rh_aparc.a2009s_thickness | rh_G.S_transv_frontopol_thickness |
| 109 | rh_aparc.a2009s_thickness | rh_S_postcentral_thickness |
| 110 | rh_aparc.a2009s_thicknessstd | rh_G.S_paracentral_thicknessstd |
| 111 | rh_aparc.a2009s_thicknessstd | rh_G_cuneus_thicknessstd |
| 112 | rh_aparc.a2009s_thicknessstd | rh_G_orbital_thicknessstd |
| 113 | rh_aparc.a2009s_volume | rh_G_parietal_sup_volume |
| 114 | rh_aparc.DKTatlas_foldind | rh_inferiorparietal_foldind |
| 115 | rh_aparc.DKTatlas_gauscurv | rh_fusiform_gauscurv |
| 116 | rh_aparc.DKTatlas_thickness | rh_insula_thickness |
| 117 | rh_aparc.DKTatlas_volume | rh_entorhinal_volume |
| 118 | rh_aparc.pial_curvind | rh_cuneus_curvind |
| 119 | rh_aparc.pial_gauscurv | rh_isthmuscingulate_gauscurv |
| 120 | rh_aparc.pial_gauscurv | rh_superiorfrontal_gauscurv |
| 121 | rh_aparc.pial_meancurv | rh_medialorbitofrontal_meancurv |
| 122 | rh_aparc.pial_meancurv | rh_pericalcarine_meancurv |
| 123 | rh_BA_exvivo_thickness | rh_BA2_exvivo_thickness |
| 124 | rh_BA_exvivo.thresh_gauscurv | rh_entorhinal_exvivo_gauscurv |
| 125 | rh.w.g.pct.std | bankssts |
| 126 | wmparc | wm.rh.middletemporal |
| 127 | lh_aparc_area | lh_fusiform_area |
| 128 | lh_aparc_area | lh_supramarginal_area |
| 129 | lh_aparc_curvind | lh_medialorbitofrontal_curvind |
| 130 | lh_aparc_curvind | lh_parsorbitalis_curvind |
| 131 | lh_aparc_curvind | lh_transversetemporal_curvind |
| 132 | lh_aparc_foldind | lh_caudalanteriorcingulate_foldind |
| 133 | lh_aparc_foldind | lh_cuneus_foldind |
| 134 | lh_aparc_foldind | lh_pericalcarine_foldind |
| 135 | lh_aparc_gauscurv | lh_medialorbitofrontal_gauscurv |
| 136 | lh_aparc_meancurv | lh_bankssts_meancurv |
| 137 | lh_aparc_meancurv | lh_entorhinal_meancurv |
| 138 | lh_aparc_meancurv | lh_superiorparietal_meancurv |
| 139 | lh_aparc_thickness | lh_inferiortemporal_thickness |
| 140 | lh_aparc_thickness | lh_parsorbitalis_thickness |
| 141 | lh_aparc_thicknessstd | lh_entorhinal_thicknessstd |
| 142 | lh_aparc_thicknessstd | lh_isthmuscingulate_thicknessstd |
| 143 | lh_aparc_volume | lh_entorhinal_volume |
| 144 | lh_aparc_volume | lh_lateralorbitofrontal_volume |
| 145 | lh_aparc_volume | lh_superiorparietal_volume |
| 146 | lh_aparc.a2009s_area | lh_G.S_cingul.Mid.Ant_area |
| 147 | lh_aparc.a2009s_area | lh_G_oc.temp_lat.fusifor_area |
| 148 | lh_aparc.a2009s_area | lh_S_collat_transv_post_area |
| 149 | lh_aparc.a2009s_area | lh_S_front_inf_area |
| 150 | lh_aparc.a2009s_curvind | lh_G_pariet_inf.Angular_curvind |
| 151 | lh_aparc.a2009s_curvind | lh_G_temporal_middle_curvind |
| 152 | lh_aparc.a2009s_curvind | lh_Pole_temporal_curvind |
| 153 | lh_aparc.a2009s_curvind | lh_S_orbital_med.olfact_curvind |
| 154 | lh_aparc.a2009s_foldind | lh_G_postcentral_foldind |
| 155 | lh_aparc.a2009s_gauscurv | lh_G_postcentral_gauscurv |
| 156 | lh_aparc.a2009s_gauscurv | lh_Lat_Fis.ant.Horizont_gauscurv |
| 157 | lh_aparc.a2009s_meancurv | lh_G_front_middle_meancurv |
| 158 | lh_aparc.a2009s_thickness | lh_G_occipital_middle_thickness |
| 159 | lh_aparc.a2009s_thickness | lh_S_calcarine_thickness |
| 160 | lh_aparc.a2009s_thickness | lh_S_orbital_med.olfact_thickness |
| 161 | lh_aparc.a2009s_thicknessstd | lh_G_insular_short_thicknessstd |
| 162 | lh_aparc.a2009s_thicknessstd | lh_S_circular_insula_inf_thicknessstd |
| 163 | lh_aparc.a2009s_thicknessstd | lh_S_oc.temp_med.Lingual_thicknessstd |
| 164 | lh_aparc.a2009s_volume | lh_G.S_frontomargin_volume |
| 165 | lh_aparc.a2009s_volume | lh_G_orbital_volume |
| 166 | lh_aparc.a2009s_volume | lh_G_rectus_volume |
| 167 | lh_aparc.a2009s_volume | lh_Pole_temporal_volume |
| 168 | lh_aparc.a2009s_volume | lh_S_circular_insula_ant_volume |
| 169 | lh_aparc.a2009s_volume | lh_S_suborbital_volume |
| 170 | lh_aparc.DKTatlas_area | lh_paracentral_area |
| 171 | lh_aparc.DKTatlas_area | lh_rostralanteriorcingulate_area |
| 172 | lh_aparc.DKTatlas_curvind | lh_lingual_curvind |
| 173 | lh_aparc.DKTatlas_curvind | lh_rostralmiddlefrontal_curvind |
| 174 | lh_aparc.DKTatlas_foldind | lh_caudalanteriorcingulate_foldind |
| 175 | lh_aparc.DKTatlas_gauscurv | lh_inferiortemporal_gauscurv |
| 176 | lh_aparc.DKTatlas_gauscurv | lh_isthmuscingulate_gauscurv |
| 177 | lh_aparc.DKTatlas_gauscurv | lh_posteriorcingulate_gauscurv |
| 178 | lh_aparc.DKTatlas_meancurv | lh_paracentral_meancurv |
| 179 | lh_aparc.DKTatlas_meancurv | lh_parsopercularis_meancurv |
| 180 | lh_aparc.DKTatlas_thicknessstd | lh_pericalcarine_thicknessstd |
| 181 | lh_aparc.DKTatlas_volume | lh_caudalmiddlefrontal_volume |
| 182 | lh_aparc.DKTatlas_volume | lh_superiorfrontal_volume |
| 183 | lh_aparc.pial_area | lh_lateralorbitofrontal_area |
| 184 | lh_aparc.pial_area | lh_medialorbitofrontal_area |
| 185 | lh_aparc.pial_area | lh_parahippocampal_area |
| 186 | lh_aparc.pial_gauscurv | lh_rostralmiddlefrontal_gauscurv |
| 187 | lh_aparc.pial_gauscurv | lh_superiorfrontal_gauscurv |
| 188 | lh_aparc.pial_gauscurv | lh_temporalpole_gauscurv |
| 189 | lh_BA_exvivo_curvind | lh_V1_exvivo_curvind |
| 190 | lh_BA_exvivo_thickness | lh_BA1_exvivo_thickness |
| 191 | lh_BA_exvivo_volume | lh_BA4p_exvivo_volume |
| 192 | lh_BA_exvivo.thresh_area | lh_BA1_exvivo_area |
| 193 | lh_BA_exvivo.thresh_curvind | lh_BA3b_exvivo_curvind |
| 194 | lh_BA_exvivo.thresh_meancurv | lh_V2_exvivo_meancurv |
| 195 | lh_BA_exvivo.thresh_volume | lh_BA2_exvivo_volume |
| 196 | rh_aparc_area | rh_caudalanteriorcingulate_area |
| 197 | rh_aparc_area | rh_middletemporal_area |
| 198 | rh_aparc_area | rh_temporalpole_area |
| 199 | rh_aparc_gauscurv | rh_paracentral_gauscurv |
| 200 | rh_aparc_meancurv | rh_caudalanteriorcingulate_meancurv |
| 201 | rh_aparc_thicknessstd | rh_superiorfrontal_thicknessstd |
| 202 | rh_aparc_volume | rh_parahippocampal_volume |
| 203 | rh_aparc.a2009s_area | rh_G.S_transv_frontopol_area |
| 204 | rh_aparc.a2009s_area | rh_G_pariet_inf.Angular_area |
| 205 | rh_aparc.a2009s_area | rh_G_postcentral_area |
| 206 | rh_aparc.a2009s_area | rh_S_front_inf_area |
| 207 | rh_aparc.a2009s_area | rh_WhiteSurfArea_area |
| 208 | rh_aparc.a2009s_foldind | rh_G_front_middle_foldind |
| 209 | rh_aparc.a2009s_foldind | rh_G_oc.temp_lat.fusifor_foldind |
| 210 | rh_aparc.a2009s_foldind | rh_G_subcallosal_foldind |
| 211 | rh_aparc.a2009s_foldind | rh_Pole_temporal_foldind |
| 212 | rh_aparc.a2009s_gauscurv | rh_G_occipital_middle_gauscurv |
| 213 | rh_aparc.a2009s_gauscurv | rh_G_oc.temp_lat.fusifor_gauscurv |
| 214 | rh_aparc.a2009s_gauscurv | rh_Lat_Fis.post_gauscurv |
| 215 | rh_aparc.a2009s_gauscurv | rh_S_orbital.H_Shaped_gauscurv |
| 216 | rh_aparc.a2009s_meancurv | rh_G_front_inf.Opercular_meancurv |
| 217 | rh_aparc.a2009s_meancurv | rh_G_front_middle_meancurv |
| 218 | rh_aparc.a2009s_meancurv | rh_G_oc.temp_lat.fusifor_meancurv |
| 219 | rh_aparc.a2009s_meancurv | rh_G_oc.temp_med.Parahip_meancurv |
| 220 | rh_aparc.a2009s_meancurv | rh_G_precuneus_meancurv |
| 221 | rh_aparc.a2009s_meancurv | rh_G_rectus_meancurv |
| 222 | rh_aparc.a2009s_meancurv | rh_S_oc_sup.transversal_meancurv |
| 223 | rh_aparc.a2009s_meancurv | rh_S_suborbital_meancurv |
| 224 | rh_aparc.a2009s_thickness | rh_G_cingul.Post.ventral_thickness |
| 225 | rh_aparc.a2009s_thicknessstd | rh_S_intrapariet.P_trans_thicknessstd |
| 226 | rh_aparc.a2009s_thicknessstd | rh_S_orbital_lateral_thicknessstd |
| 227 | rh_aparc.a2009s_volume | rh_G.S_cingul.Mid.Ant_volume |
| 228 | rh_aparc.a2009s_volume | rh_G_occipital_middle_volume |
| 229 | rh_aparc.a2009s_volume | rh_S_precentral.inf.part_volume |
| 230 | rh_aparc.a2009s_volume | rh_S_temporal_sup_volume |
| 231 | rh_aparc.DKTatlas_area | rh_transversetemporal_area |
| 232 | rh_aparc.DKTatlas_curvind | rh_inferiortemporal_curvind |
| 233 | rh_aparc.DKTatlas_foldind | rh_precuneus_foldind |
| 234 | rh_aparc.DKTatlas_gauscurv | rh_inferiorparietal_gauscurv |
| 235 | rh_aparc.DKTatlas_gauscurv | rh_parahippocampal_gauscurv |
| 236 | rh_aparc.DKTatlas_gauscurv | rh_posteriorcingulate_gauscurv |
| 237 | rh_aparc.DKTatlas_meancurv | rh_parstriangularis_meancurv |
| 238 | rh_aparc.DKTatlas_thicknessstd | rh_fusiform_thicknessstd |
| 239 | rh_aparc.DKTatlas_volume | rh_caudalanteriorcingulate_volume |
| 240 | rh_aparc.DKTatlas_volume | rh_superiortemporal_volume |
| 241 | rh_aparc.pial_area | rh_medialorbitofrontal_area |
| 242 | rh_aparc.pial_area | rh_postcentral_area |
| 243 | rh_aparc.pial_meancurv | rh_parsopercularis_meancurv |
| 244 | rh_BA_exvivo_area | rh_perirhinal_exvivo_area |
| 245 | rh_BA_exvivo_foldind | rh_perirhinal_exvivo_foldind |
| 246 | rh_BA_exvivo_gauscurv | rh_entorhinal_exvivo_gauscurv |
| 247 | rh_BA_exvivo_meancurv | rh_perirhinal_exvivo_meancurv |
| 248 | rh_BA_exvivo_thickness | rh_BA4p_exvivo_thickness |
| 249 | rh_BA_exvivo_thickness | rh_entorhinal_exvivo_thickness |
| 250 | rh_BA_exvivo_thicknessstd | rh_BA1_exvivo_thicknessstd |
| 251 | rh_BA_exvivo_thicknessstd | rh_BA2_exvivo_thicknessstd |
| 252 | rh_BA_exvivo_thicknessstd | rh_perirhinal_exvivo_thicknessstd |
| 253 | rh_BA_exvivo_volume | rh_BA4p_exvivo_volume |
| 254 | rh_BA_exvivo.thresh_area | rh_MT_exvivo_area |
| 255 | rh_BA_exvivo.thresh_curvind | rh_BA3a_exvivo_curvind |
| 256 | rh_BA_exvivo.thresh_foldind | rh_BA2_exvivo_foldind |
| 257 | aseg | CC_Posterior |
| 258 | aseg | EstimatedTotalIntraCranialVol |
| 259 | lh.w.g.pct.mean | medialorbitofrontal |
| 260 | rh.w.g.pct.mean | entorhinal |
| 261 | rh.w.g.pct.mean | superiorparietal |
| 262 | rh.w.g.pct.std | medialorbitofrontal |
| 263 | rh.w.g.pct.std | middletemporal |
| 264 | rh.w.g.pct.std | parahippocampal |
| 265 | wmparc | wm.rh.temporalpole |
| 266 | lh_aparc_area | lh_inferiortemporal_area |
| 267 | lh_aparc_area | lh_temporalpole_area |
| 268 | lh_aparc_curvind | lh_inferiorparietal_curvind |
| 269 | lh_aparc_curvind | lh_rostralmiddlefrontal_curvind |
| 270 | lh_aparc_foldind | lh_inferiorparietal_foldind |
| 271 | lh_aparc_gauscurv | lh_precentral_gauscurv |
| 272 | lh_aparc_gauscurv | lh_precuneus_gauscurv |
| 273 | lh_aparc_gauscurv | lh_superiorfrontal_gauscurv |
| 274 | lh_aparc_meancurv | lh_postcentral_meancurv |
| 275 | lh_aparc_meancurv | lh_rostralmiddlefrontal_meancurv |
| 276 | lh_aparc_thickness | lh_caudalanteriorcingulate_thickness |
| 277 | lh_aparc_thickness | lh_medialorbitofrontal_thickness |
| 278 | lh_aparc_thickness | lh_parahippocampal_thickness |
| 279 | lh_aparc_thickness | lh_parsopercularis_thickness |
| 280 | lh_aparc_thickness | lh_precuneus_thickness |
| 281 | lh_aparc_thicknessstd | lh_paracentral_thicknessstd |
| 282 | lh_aparc_volume | lh_rostralanteriorcingulate_volume |
| 283 | lh_aparc_volume | lh_transversetemporal_volume |
| 284 | lh_aparc.a2009s_area | lh_G_Ins_lg.S_cent_ins_area |
| 285 | lh_aparc.a2009s_area | lh_G_temp_sup.Lateral_area |
| 286 | lh_aparc.a2009s_area | lh_G_temporal_middle_area |
| 287 | lh_aparc.a2009s_area | lh_S_calcarine_area |
| 288 | lh_aparc.a2009s_area | lh_S_circular_insula_inf_area |
| 289 | lh_aparc.a2009s_area | lh_S_oc_sup.transversal_area |
| 290 | lh_aparc.a2009s_curvind | lh_G.S_cingul.Mid.Post_curvind |
| 291 | lh_aparc.a2009s_curvind | lh_G_front_inf.Triangul_curvind |
| 292 | lh_aparc.a2009s_curvind | lh_G_front_middle_curvind |
| 293 | lh_aparc.a2009s_curvind | lh_G_rectus_curvind |
| 294 | lh_aparc.a2009s_curvind | lh_Lat_Fis.post_curvind |
| 295 | lh_aparc.a2009s_foldind | lh_G.S_subcentral_foldind |
| 296 | lh_aparc.a2009s_foldind | lh_G.S_cingul.Ant_foldind |
| 297 | lh_aparc.a2009s_foldind | lh_G_cuneus_foldind |
| 298 | lh_aparc.a2009s_foldind | lh_G_precentral_foldind |
| 299 | lh_aparc.a2009s_foldind | lh_G_temp_sup.G_T_transv_foldind |
| 300 | lh_aparc.a2009s_foldind | lh_S_collat_transv_ant_foldind |

## Supplementary Table S4

**Top 300 features selected from the CBDA analysis on the UK Biobank with neuroimaging biomarkers and clinical features (Figure 6B)**

**Yellow highlights: clinical features**

**Green highlights: overlap with Table S3**

| **Rank** | **Atlas** | **Description** |
| --- | --- | --- |
| 1 | X2020.0.0 Loneliness/isolation | |
| 2 | rh_aparc_meancurv | rh_medialorbitofrontal_meancurv |
| 3 | rh_aparc.pial_gauscurv | rh_insula_gauscurv |
| 4 | lh.w.g.pct.mean | caudalanteriorcingulate |
| 5 | lh_aparc.DKTatlas_meancurv | lh_insula_meancurv |
| 6 | lh_BA_exvivo.thresh_gauscurv | lh_perirhinal_exvivo_gauscurv |
| 7 | rh_aparc.a2009s_meancurv | rh_G.S_transv_frontopol_meancurv |
| 8 | rh_aparc.a2009s_volume | rh_S_collat_transv_ant_volume |
| 9 | rh_aparc.DKTatlas_area | rh_superiorfrontal_area |
| 10 | rh_aparc.DKTatlas_thickness | rh_superiortemporal_thickness |
| 11 | 25018 Volume of pallidum (right) T1 structural brain MRI | |
| 12 | 25094 Mean FA in fornix cres+stria terminalis on FA skeleton (right) dMRI skeleton | |
| 13 | lh_aparc_gauscurv | lh_fusiform_gauscurv |
| 14 | lh_aparc.a2009s_foldind | lh_S_subparietal_foldind |
| 15 | lh_aparc.a2009s_meancurv | lh_S_cingul.Marginalis_meancurv |
| 16 | lh_aparc.DKTatlas_area | lh_pericalcarine_area |
| 17 | lh_aparc.DKTatlas_volume | lh_superiorparietal_volume |
| 18 | lh_aparc.pial_area | lh_parsopercularis_area |
| 19 | lh_aparc.pial_area | lh_frontalpole_area |
| 20 | rh_aparc.DKTatlas_volume | rh_superiortemporal_volume |
| 21 | rh_aparc.pial_curvind | rh_temporalpole_curvind |
| 22 | rh_aparc.pial_gauscurv | rh_superiorparietal_gauscurv |
| 23 | rh_BA_exvivo_foldind | rh_MT_exvivo_foldind |
| 24 | 20160 Ever smoked Smoking | |
| 25 | 25498 Weighted-mean FA in tract forceps major dMRI weighted means | |
| 26 | 25704 Weighted-mean ISOVF in tract acoustic radiation (left) dMRI weighted means | |
| 27 | lh_aparc_curvind | lh_entorhinal_curvind |
| 28 | lh_aparc_meancurv | lh_caudalanteriorcingulate_meancurv |
| 29 | lh_aparc.a2009s_foldind | lh_G.S_cingul.Mid.Post_foldind |
| 30 | lh_aparc.a2009s_foldind | lh_G_temp_sup.Plan_tempo_foldind |
| 31 | lh_aparc.a2009s_foldind | lh_S_interm_prim.Jensen_foldind |
| 32 | lh_aparc.a2009s_meancurv | lh_S_suborbital_meancurv |
| 33 | lh_aparc.a2009s_volume | lh_G_pariet_inf.Angular_volume |
| 34 | lh_aparc.DKTatlas_curvind | lh_parsorbitalis_curvind |
| 35 | lh_aparc.DKTatlas_foldind | lh_inferiortemporal_foldind |
| 36 | lh_aparc.DKTatlas_volume | lh_parstriangularis_volume |
| 37 | lh_aparc.pial_curvind | lh_cuneus_curvind |
| 38 | lh_aparc.pial_meancurv | lh_parsorbitalis_meancurv |
| 39 | rh_aparc_foldind | rh_inferiortemporal_foldind |
| 40 | rh_aparc_meancurv | rh_rostralanteriorcingulate_meancurv |
| 41 | rh_aparc.a2009s_area | rh_S_occipital_ant_area |
| 42 | rh_aparc.a2009s_meancurv | rh_G_rectus_meancurv |
| 43 | rh_aparc.a2009s_meancurv | rh_S_cingul.Marginalis_meancurv |
| 44 | rh_aparc.a2009s_thickness | rh_G_temp_sup.Plan_tempo_thickness |
| 45 | rh_aparc.a2009s_thicknessstd | rh_S_temporal_transverse_thicknessstd |
| 46 | rh_aparc.DKTatlas_foldind | rh_lateraloccipital_foldind |
| 47 | rh_aparc.DKTatlas_thicknessstd | rh_parsopercularis_thicknessstd |
| 48 | rh_aparc.DKTatlas_volume | rh_rostralanteriorcingulate_volume |
| 49 | rh_aparc.pial_area | rh_precentral_area |
| 50 | rh_BA_exvivo_area | rh_BA3a_exvivo_area |
| 51 | rh_BA_exvivo.thresh_thicknessstd | rh_MT_exvivo_thicknessstd |
| 52 | wmparc | wm.lh.cuneus |
| 53 | 25121 Mean MD in anterior limb of internal capsule on FA skeleton (left) dMRI skeleton | |
| 54 | 25238 Mean L1 in fornix cres+stria terminalis on FA skeleton (right) dMRI skeleton | |
| 55 | 25274 Mean L2 in posterior corona radiata on FA skeleton (right) dMRI skeleton | |
| 56 | 25353 Mean ICVF in medial lemniscus on FA skeleton (left) dMRI skeleton | |
| 57 | 25530 Weighted-mean MD in tract inferior longitudinal fasciculus (right) dMRI weighted means | |
| 58 | 25595 Weighted-mean L1 in tract uncinate fasciculus (right) dMRI weighted means | |
| 59 | 25607 Weighted-mean L2 in tract forceps minor dMRI weighted means | |
| 60 | lh_aparc_gauscurv | lh_parsorbitalis_gauscurv |
| 61 | lh_aparc_meancurv | lh_lateralorbitofrontal_meancurv |
| 62 | lh_aparc.a2009s_area | lh_G_oc.temp_med.Parahip_area |
| 63 | lh_aparc.a2009s_curvind | lh_G_occipital_middle_curvind |
| 64 | lh_aparc.a2009s_curvind | lh_S_front_inf_curvind |
| 65 | lh_aparc.a2009s_foldind | lh_Lat_Fis.post_foldind |
| 66 | lh_aparc.a2009s_meancurv | lh_S_subparietal_meancurv |
| 67 | lh_aparc.a2009s_thicknessstd | lh_S_oc_middle.Lunatus_thicknessstd |
| 68 | lh_aparc.DKTatlas_area | lh_lingual_area |
| 69 | lh_aparc.DKTatlas_foldind | lh_posteriorcingulate_foldind |
| 70 | lh_aparc.DKTatlas_foldind | lh_transversetemporal_foldind |
| 71 | lh_aparc.DKTatlas_meancurv | lh_precuneus_meancurv |
| 72 | lh_aparc.DKTatlas_thicknessstd | lh_fusiform_thicknessstd |
| 73 | lh_aparc.DKTatlas_thicknessstd | lh_lateraloccipital_thicknessstd |
| 74 | lh_aparc.pial_meancurv | lh_pericalcarine_meancurv |
| 75 | lh_BA_exvivo_thickness | lh_BA3a_exvivo_thickness |
| 76 | lh_BA_exvivo_thickness | lh_BA6_exvivo_thickness |
| 77 | lh_BA_exvivo.thresh_thicknessstd | lh_BA1_exvivo_thicknessstd |
| 78 | rh_aparc_thickness | rh_entorhinal_thickness |
| 79 | rh_aparc_thickness | rh_precuneus_thickness |
| 80 | rh_aparc_thickness | rh_temporalpole_thickness |
| 81 | rh_aparc_volume | rh_entorhinal_volume |
| 82 | rh_aparc.a2009s_area | rh_S_orbital_lateral_area |
| 83 | rh_aparc.a2009s_area | rh_S_suborbital_area |
| 84 | rh_aparc.a2009s_curvind | rh_G.S_cingul.Ant_curvind |
| 85 | rh_aparc.a2009s_curvind | rh_G_front_sup_curvind |
| 86 | rh_aparc.a2009s_curvind | rh_G_occipital_middle_curvind |
| 87 | rh_aparc.a2009s_curvind | rh_G_oc.temp_lat.fusifor_curvind |
| 88 | rh_aparc.a2009s_foldind | rh_G.S_frontomargin_foldind |
| 89 | rh_aparc.a2009s_foldind | rh_S_intrapariet.P_trans_foldind |
| 90 | rh_aparc.a2009s_meancurv | rh_G_postcentral_meancurv |
| 91 | rh_aparc.a2009s_meancurv | rh_S_occipital_ant_meancurv |
| 92 | rh_aparc.a2009s_thickness | rh_G_temporal_inf_thickness |
| 93 | rh_aparc.a2009s_thickness | rh_S_suborbital_thickness |
| 94 | rh_aparc.a2009s_thicknessstd | rh_S_pericallosal_thicknessstd |
| 95 | rh_aparc.a2009s_volume | rh_G_rectus_volume |
| 96 | rh_aparc.a2009s_volume | rh_G_subcallosal_volume |
| 97 | rh_aparc.a2009s_volume | rh_S_circular_insula_sup_volume |
| 98 | rh_aparc.a2009s_volume | rh_S_collat_transv_post_volume |
| 99 | rh_aparc.DKTatlas_area | rh_paracentral_area |
| 100 | rh_aparc.DKTatlas_foldind | rh_superiorparietal_foldind |
| 101 | rh_aparc.DKTatlas_thickness | rh_parahippocampal_thickness |
| 102 | rh_aparc.DKTatlas_thickness | rh_parstriangularis_thickness |
| 103 | rh_aparc.DKTatlas_thickness | rh_superiorfrontal_thickness |
| 104 | rh_aparc.pial_area | rh_supramarginal_area |
| 105 | rh_BA_exvivo_foldind | rh_BA4a_exvivo_foldind |
| 106 | rh_BA_exvivo.thresh_area | rh_BA3b_exvivo_area |
| 107 | rh_BA_exvivo.thresh_meancurv | rh_MT_exvivo_meancurv |
| 108 | aseg | Left.Putamen |
| 109 | aseg | Right.choroid.plexus |
| 110 | lh.w.g.pct.std | lateraloccipital |
| 111 | rh.w.g.pct.std | inferiortemporal |
| 112 | rh.w.g.pct.std | parstriangularis |
| 113 | wmparc | wm.rh.lateralorbitofrontal |
| 114 | wmparc | wm.rh.parsopercularis |
| 115 | 2050 Frequency of depressed mood in last 2 weeks Mental health | |
| 116 | 25001 Volume of peripheral cortical grey matter (normalised for head size) T1 structural brain MRI | |
| 117 | 25035 Median T2star in hippocampus (right) Susceptibility weighted brain MRI | |
| 118 | 25086 Mean FA in sagittal stratum on FA skeleton (right) dMRI skeleton | |
| 119 | 25234 Mean L1 in cingulum cingulate gyrus on FA skeleton (right) dMRI skeleton | |
| 120 | 25261 Mean L2 in superior cerebellar peduncle on FA skeleton (left) dMRI skeleton | |
| 121 | 25262 Mean L2 in cerebral peduncle on FA skeleton (right) dMRI skeleton | |
| 122 | 25273 Mean L2 in superior corona radiata on FA skeleton (left) dMRI skeleton | |
| 123 | 25307 Mean L3 in inferior cerebellar peduncle on FA skeleton (left) dMRI skeleton | |
| 124 | 25338 Mean L3 in superior fronto-occipital fasciculus on FA skeleton (right) dMRI skeleton | |
| 125 | 25350 Mean ICVF in corticospinal tract on FA skeleton (right) dMRI skeleton | |
| 126 | 25436 Mean OD in uncinate fasciculus on FA skeleton (right) dMRI skeleton | |
| 127 | 25486 Mean ISOVF in tapetum on FA skeleton (right) dMRI skeleton | |
| 128 | 25533 Weighted-mean MD in tract medial lemniscus (right) dMRI weighted means | |
| 129 | 25543 Weighted-mean MO in tract acoustic radiation (right) dMRI weighted means | |
| 130 | 25551 Weighted-mean MO in tract corticospinal tract (right) dMRI weighted means | |
| 131 | 25575 Weighted-mean L1 in tract parahippocampal part of cingulum (left) dMRI weighted means | |
| 132 | 25591 Weighted-mean L1 in tract superior longitudinal fasciculus (right) dMRI weighted means | |
| 133 | 25703 Weighted-mean OD in tract uncinate fasciculus (right) dMRI weighted means | |
| 134 | lh_aparc_thicknessstd | lh_supramarginal_thicknessstd |
| 135 | lh_aparc.a2009s_area | lh_G.S_cingul.Ant_area |
| 136 | lh_aparc.a2009s_area | lh_G.S_cingul.Mid.Ant_area |
| 137 | lh_aparc.a2009s_area | lh_G_cingul.Post.dorsal_area |
| 138 | lh_aparc.a2009s_area | lh_G_cuneus_area |
| 139 | lh_aparc.a2009s_area | lh_G_front_sup_area |
| 140 | lh_aparc.a2009s_curvind | lh_Lat_Fis.ant.Vertical_curvind |
| 141 | lh_aparc.a2009s_curvind | lh_S_occipital_ant_curvind |
| 142 | lh_aparc.a2009s_curvind | lh_S_temporal_transverse_curvind |
| 143 | lh_aparc.a2009s_foldind | lh_G.S_subcentral_foldind |
| 144 | lh_aparc.a2009s_foldind | lh_G_Ins_lg.S_cent_ins_foldind |
| 145 | lh_aparc.a2009s_foldind | lh_G_rectus_foldind |
| 146 | lh_aparc.a2009s_foldind | lh_G_temp_sup.Lateral_foldind |
| 147 | lh_aparc.a2009s_foldind | lh_S_front_sup_foldind |
| 148 | lh_aparc.a2009s_foldind | lh_S_precentral.inf.part_foldind |
| 149 | lh_aparc.a2009s_meancurv | lh_G_temp_sup.Plan_polar_meancurv |
| 150 | lh_aparc.a2009s_meancurv | lh_S_front_middle_meancurv |
| 151 | lh_aparc.a2009s_meancurv | lh_S_interm_prim.Jensen_meancurv |
| 152 | lh_aparc.a2009s_thickness | lh_G_front_inf.Triangul_thickness |
| 153 | lh_aparc.a2009s_thickness | lh_G_orbital_thickness |
| 154 | lh_aparc.a2009s_thickness | lh_G_precentral_thickness |
| 155 | lh_aparc.a2009s_thicknessstd | lh_G.S_cingul.Mid.Post_thicknessstd |
| 156 | lh_aparc.a2009s_thicknessstd | lh_S_oc.temp_med.Lingual_thicknessstd |
| 157 | lh_aparc.a2009s_volume | lh_G_cuneus_volume |
| 158 | lh_aparc.a2009s_volume | lh_G_temporal_middle_volume |
| 159 | lh_aparc.a2009s_volume | lh_S_temporal_transverse_volume |
| 160 | lh_aparc.DKTatlas_curvind | lh_entorhinal_curvind |
| 161 | lh_aparc.DKTatlas_foldind | lh_superiorfrontal_foldind |
| 162 | lh_aparc.DKTatlas_gauscurv | lh_lateralorbitofrontal_gauscurv |
| 163 | lh_aparc.DKTatlas_thickness | lh_parahippocampal_thickness |
| 164 | lh_aparc.DKTatlas_volume | lh_entorhinal_volume |
| 165 | lh_aparc.pial_area | lh_lingual_area |
| 166 | lh_aparc.pial_area | lh_superiortemporal_area |
| 167 | lh_aparc.pial_curvind | lh_rostralmiddlefrontal_curvind |
| 168 | lh_aparc.pial_gauscurv | lh_inferiortemporal_gauscurv |
| 169 | lh_aparc.pial_gauscurv | lh_supramarginal_gauscurv |
| 170 | lh_aparc.pial_meancurv | lh_entorhinal_meancurv |
| 171 | lh_BA_exvivo_area | lh_BA4p_exvivo_area |
| 172 | lh_BA_exvivo_foldind | lh_BA45_exvivo_foldind |
| 173 | lh_BA_exvivo_gauscurv | lh_perirhinal_exvivo_gauscurv |
| 174 | lh_BA_exvivo_thicknessstd | lh_BA4p_exvivo_thicknessstd |
| 175 | lh_BA_exvivo.thresh_thicknessstd | lh_V2_exvivo_thicknessstd |
| 176 | rh_aparc_area | rh_middletemporal_area |
| 177 | rh_aparc_gauscurv | rh_lateralorbitofrontal_gauscurv |
| 178 | rh_aparc_meancurv | rh_superiorparietal_meancurv |
| 179 | rh_aparc_thickness | rh_rostralanteriorcingulate_thickness |
| 180 | rh_aparc_thickness | rh_superiorfrontal_thickness |
| 181 | rh_aparc_thicknessstd | rh_lateraloccipital_thicknessstd |
| 182 | rh_aparc_thicknessstd | rh_postcentral_thicknessstd |
| 183 | rh_aparc_thicknessstd | rh_superiorfrontal_thicknessstd |
| 184 | rh_aparc_thicknessstd | rh_superiortemporal_thicknessstd |
| 185 | rh_aparc.a2009s_area | rh_G_front_inf.Opercular_area |
| 186 | rh_aparc.a2009s_area | rh_G_Ins_lg.S_cent_ins_area |
| 187 | rh_aparc.a2009s_area | rh_G_oc.temp_med.Lingual_area |
| 188 | rh_aparc.a2009s_area | rh_G_temp_sup.Plan_polar_area |
| 189 | rh_aparc.a2009s_area | rh_G_temp_sup.Plan_tempo_area |
| 190 | rh_aparc.a2009s_area | rh_S_calcarine_area |
| 191 | rh_aparc.a2009s_curvind | rh_G_front_inf.Opercular_curvind |
| 192 | rh_aparc.a2009s_curvind | rh_S_cingul.Marginalis_curvind |
| 193 | rh_aparc.a2009s_curvind | rh_S_interm_prim.Jensen_curvind |
| 194 | rh_aparc.a2009s_foldind | rh_G.S_occipital_inf_foldind |
| 195 | rh_aparc.a2009s_foldind | rh_S_orbital.H_Shaped_foldind |
| 196 | rh_aparc.a2009s_foldind | rh_S_suborbital_foldind |
| 197 | rh_aparc.a2009s_gauscurv | rh_S_front_middle_gauscurv |
| 198 | rh_aparc.a2009s_gauscurv | rh_S_occipital_ant_gauscurv |
| 199 | rh_aparc.a2009s_gauscurv | rh_S_orbital.H_Shaped_gauscurv |
| 200 | rh_aparc.a2009s_gauscurv | rh_S_postcentral_gauscurv |
| 201 | rh_aparc.a2009s_meancurv | rh_G_temp_sup.Plan_tempo_meancurv |
| 202 | rh_aparc.a2009s_meancurv | rh_S_oc_sup.transversal_meancurv |
| 203 | rh_aparc.a2009s_thickness | rh_G_front_sup_thickness |
| 204 | rh_aparc.a2009s_thickness | rh_G_pariet_inf.Supramar_thickness |
| 205 | rh_aparc.a2009s_thickness | rh_S_circular_insula_sup_thickness |
| 206 | rh_aparc.a2009s_thicknessstd | rh_S_suborbital_thicknessstd |
| 207 | rh_aparc.a2009s_volume | rh_G_front_middle_volume |
| 208 | rh_aparc.a2009s_volume | rh_G_temporal_inf_volume |
| 209 | rh_aparc.DKTatlas_area | rh_entorhinal_area |
| 210 | rh_aparc.DKTatlas_curvind | rh_paracentral_curvind |
| 211 | rh_aparc.DKTatlas_foldind | rh_fusiform_foldind |
| 212 | rh_aparc.DKTatlas_foldind | rh_isthmuscingulate_foldind |
| 213 | rh_aparc.DKTatlas_gauscurv | rh_parsopercularis_gauscurv |
| 214 | rh_aparc.DKTatlas_meancurv | rh_cuneus_meancurv |
| 215 | rh_aparc.DKTatlas_meancurv | rh_superiortemporal_meancurv |
| 216 | rh_aparc.DKTatlas_meancurv | rh_transversetemporal_meancurv |
| 217 | rh_aparc.DKTatlas_thickness | rh_caudalmiddlefrontal_thickness |
| 218 | rh_aparc.DKTatlas_thickness | rh_paracentral_thickness |
| 219 | rh_aparc.DKTatlas_thicknessstd | rh_cuneus_thicknessstd |
| 220 | rh_aparc.DKTatlas_thicknessstd | rh_precuneus_thicknessstd |
| 221 | rh_aparc.DKTatlas_volume | rh_parahippocampal_volume |
| 222 | rh_aparc.DKTatlas_volume | rh_rostralmiddlefrontal_volume |
| 223 | rh_aparc.pial_area | rh_frontalpole_area |
| 224 | rh_aparc.pial_foldind | rh_precuneus_foldind |
| 225 | rh_aparc.pial_gauscurv | rh_caudalanteriorcingulate_gauscurv |
| 226 | rh_aparc.pial_gauscurv | rh_caudalmiddlefrontal_gauscurv |
| 227 | rh_aparc.pial_gauscurv | rh_paracentral_gauscurv |
| 228 | rh_aparc.pial_gauscurv | rh_supramarginal_gauscurv |
| 229 | rh_aparc.pial_meancurv | rh_parstriangularis_meancurv |
| 230 | rh_BA_exvivo_curvind | rh_BA2_exvivo_curvind |
| 231 | rh_BA_exvivo_gauscurv | rh_perirhinal_exvivo_gauscurv |
| 232 | rh_BA_exvivo_volume | rh_BA4p_exvivo_volume |
| 233 | rh_BA_exvivo_volume | rh_V2_exvivo_volume |
| 234 | rh_BA_exvivo.thresh_gauscurv | rh_BA3a_exvivo_gauscurv |
| 235 | aseg | Left.Amygdala |
| 236 | aseg | CortexVol |
| 237 | lh.w.g.pct.mean | pericalcarine |
| 238 | lh.w.g.pct.mean | precentral |
| 239 | rh.w.g.pct.mean | inferiorparietal |
| 240 | rh.w.g.pct.mean | rostralanteriorcingulate |
| 241 | rh.w.g.pct.mean | insula |
| 242 | wmparc | wm.lh.inferiortemporal |
| 243 | wmparc | wm.rh.supramarginal |
| 244 | X22000.0.0: Genotype measurement batch | |
| 245 | X25015.2.0:Volume of putamen (left) | |
| 246 | X25021.2.0:Volume of amygdala (left) | |
| 247 | X25071.2.0:Mean FA in cerebral peduncle on FA skeleton (left) | |
| 248 | X25072.2.0:Mean FA in anterior limb of internal capsule on FA skeleton (right) | |
| 249 | X25081.2.0:Mean FA in superior corona radiata on FA skeleton (left) | |
| 250 | X25092.2.0:Mean FA in cingulum hippocampus on FA skeleton (right) | |
| 251 | X25103.2.0:Mean FA in tapetum on FA skeleton (left) | |
| 252 | X25143.2.0:Mean MD in fornix cres+stria terminalis on FA skeleton (left) | |
| 253 | X25145.2.0:Mean MD in superior longitudinal fasciculus on FA skeleton (left) | |
| 254 | X25256.2.0:Mean L2 in medial lemniscus on FA skeleton (right) | |
| 255 | X25257.2.0:Mean L2 in medial lemniscus on FA skeleton (left) | |
| 256 | X25270.2.0:Mean L2 in anterior corona radiata on FA skeleton (right) | |
| 257 | X25303.2.0:Mean L3 in corticospinal tract on FA skeleton (left) | |
| 258 | X25328.2.0:Mean L3 in external capsule on FA skeleton (right) | |
| 259 | X25331.2.0:Mean L3 in cingulum cingulate gyrus on FA skeleton (left) | |
| 260 | X25414.2.0:Mean OD in anterior corona radiata on FA skeleton (right) | |
| 261 | X25474.2.0:Mean ISOVF in cingulum cingulate gyrus on FA skeleton (right) | |
| 262 | X25568.2.0:Weighted-mean MO in tract uncinate fasciculus (right) | |
| 263 | X25571.2.0:Weighted-mean L1 in tract anterior thalamic radiation (left) | |
| 264 | X25647.2.0:Weighted-mean L3 in tract superior thalamic radiation (right) | |
| 265 | X25725.2.0:Weighted-mean ISOVF in tract superior longitudinal fasciculus (left) | |
| 266 | X25735.2.0:Inverted contrast-to-noise ratio in T1 structural brain MRI | |
| 267 | X25766.2.0:90th percentile of z-statistic (in group-defined mask) for faces-shapes contrast | |
| 268 | lh_aparc_area | lh_inferiorparietal_area |
| 269 | lh_aparc_foldind | lh_caudalanteriorcingulate_foldind |
| 270 | lh_aparc_foldind | lh_cuneus_foldind |
| 271 | lh_aparc_foldind | lh_fusiform_foldind |
| 272 | lh_aparc_foldind | lh_parsopercularis_foldind |
| 273 | lh_aparc_foldind | lh_supramarginal_foldind |
| 274 | lh_aparc_foldind | lh_temporalpole_foldind |
| 275 | lh_aparc_gauscurv | lh_parsopercularis_gauscurv |
| 276 | lh_aparc_thickness | lh_caudalanteriorcingulate_thickness |
| 277 | lh_aparc_thickness | lh_inferiortemporal_thickness |
| 278 | lh_aparc_thickness | lh_temporalpole_thickness |
| 279 | lh_aparc_thicknessstd | lh_medialorbitofrontal_thicknessstd |
| 280 | lh_aparc_volume | lh_isthmuscingulate_volume |
| 281 | lh_aparc.a2009s_area | lh_G.S_paracentral_area |
| 282 | lh_aparc.a2009s_area | lh_G_insular_short_area |
| 283 | lh_aparc.a2009s_curvind | lh_G.S_frontomargin_curvind |
| 284 | lh_aparc.a2009s_curvind | lh_G_pariet_inf.Supramar_curvind |
| 285 | lh_aparc.a2009s_foldind | lh_G_front_sup_foldind |
| 286 | lh_aparc.a2009s_foldind | lh_S_circular_insula_sup_foldind |
| 287 | lh_aparc.a2009s_gauscurv | lh_G.S_transv_frontopol_gauscurv |
| 288 | lh_aparc.a2009s_gauscurv | lh_G.S_cingul.Ant_gauscurv |
| 289 | lh_aparc.a2009s_gauscurv | lh_G_pariet_inf.Supramar_gauscurv |
| 290 | lh_aparc.a2009s_gauscurv | lh_S_central_gauscurv |
| 291 | lh_aparc.a2009s_gauscurv | lh_S_orbital.H_Shaped_gauscurv |
| 292 | lh_aparc.a2009s_meancurv | lh_S_intrapariet.P_trans_meancurv |
| 293 | lh_aparc.a2009s_thickness | lh_S_oc_sup.transversal_thickness |
| 294 | lh_aparc.a2009s_thicknessstd | lh_G.S_occipital_inf_thicknessstd |
| 295 | lh_aparc.a2009s_thicknessstd | lh_G_occipital_sup_thicknessstd |
| 296 | lh_aparc.a2009s_thicknessstd | lh_G_pariet_inf.Angular_thicknessstd |
| 297 | lh_aparc.a2009s_thicknessstd | lh_S_front_middle_thicknessstd |
| 298 | lh_aparc.a2009s_thicknessstd | lh_S_oc_sup.transversal_thicknessstd |
| 299 | lh_aparc.a2009s_volume | lh_G_precuneus_volume |
| 300 | lh_aparc.a2009s_volume | lh_Lat_Fis.post_volume |

## Supplementary Table S5

**Overlap between the top 300 features listed in the Supplementary Tables S3 and S4**

| **Feature** | **Region** | **Hemisphere** | **Morphometric** |
| --- | --- | --- | --- |
| "lh_entorhinal_volume" | Entorhinal Cortex | L | **3D Volume** |
| "rh_entorhinal_volume” |  | R |  |
| "rh_parahippocampal_volume” | Para-hippocampal |  |  |
| ”rh_BA4p_exvivo_volume” | Primary Motor Cortex (BA4p) |  |  |
| "lh_superiorparietal_volume" | Superior-parietal Lobe | L |  |
| "rh_superiortemporal_volume” | Superior-temporal | R |  |
| ”lh_caudalanteriorcingulate_thickness" | Caudal-anterior-cingulate Gyrus | L | **Cortical thickness** |
| "lh_fusiform_thicknessstd” | Fusiform gyrus |  |  |
| ”lh_inferiortemporal_thickness" | Inferior-temporal |  |  |
| ”rh_lateraloccipital_thicknessstd" | Lateral-occipital | R |  |
| "lh_parahippocampal_thickness" | Para-hippocampal | L |  |
| "rh_parahippocampal_thickness” |  | R |  |
| "lh_G_precentral_thickness" | Precentral gyrus | L |  |
| ”lh_BA4p_exvivo_thicknessstd” | Primary Motor Cortex (BA4p) |  |  |
| ”rh_superiorfrontal_thicknessstd" | Superior-frontal | R |  |
| "lh_supramarginal_thicknessstd” | Supra-marginal | L |  |
| ”lh_fusiform_gauscurv” | Fusiform Gyrus |  | **Curvature (Gaussian)** |
| "lh_inferiortemporal_gauscurv” | Inferior-temporal |  |  |
| ”lh_lateralorbitofrontal_gauscurv" | Lateral-orbito-frontal Gyrus |  |  |
| ”rh_paracentral_gauscurv” | Paracentral Gyrus | R |  |
| ”lh_parsorbitalis_curvind" | Parsorbitalis | L | **Curvature (Index)** |
| "lh_rostralmiddlefrontal_curvind" | Rostral-middle-frontal |  |  |
| ”lh_entorhinal_meancurv” | Entorhinal Cortex |  | **Curvature (mean)** |
| "lh_lateralorbitofrontal_meancurv” | Lateral-orbito-frontal Gyrus |  |  |
| ”rh_medialorbitofrontal_meancurv” | Medial-orbito-frontal | R |  |
| ”rh_parstriangularis_meancurv" | Parstriangularis |  |  |
| "lh_precuneus_meancurv” | Precuneus | L |  |
| ”rh_G_rectus_meancurv” | Rectus Gyrus | R |  |
| ”lh_caudalanteriorcingulate_foldind" | Caudal-anterior-cingulate Gyrus | L | **Gyral Folding Index** |
| "lh_cuneus_foldind” | Cuneus |  |  |
| ”rh_precuneus_foldind" | Precuneus | R |  |
| ”rh_entorhinal_area” | Entorhinal |  | **Surface Area** |
| ”rh_middletemporal_area” | Middle-temporal |  |  |
| ”lh_parsopercularis_area" | Parsopercularis | L |  |

## Supplementary Figure S1 - Schematic of the Pseudocode of the CBDA 2.0 algorithm


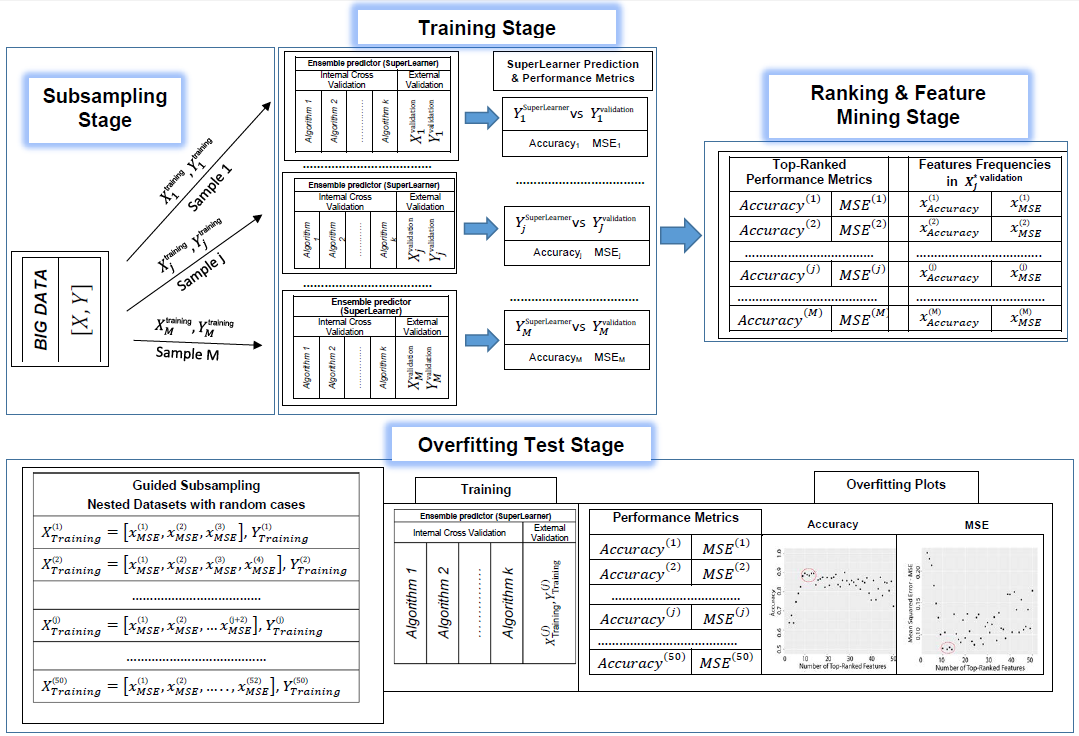


**Supplementary Figure S2**


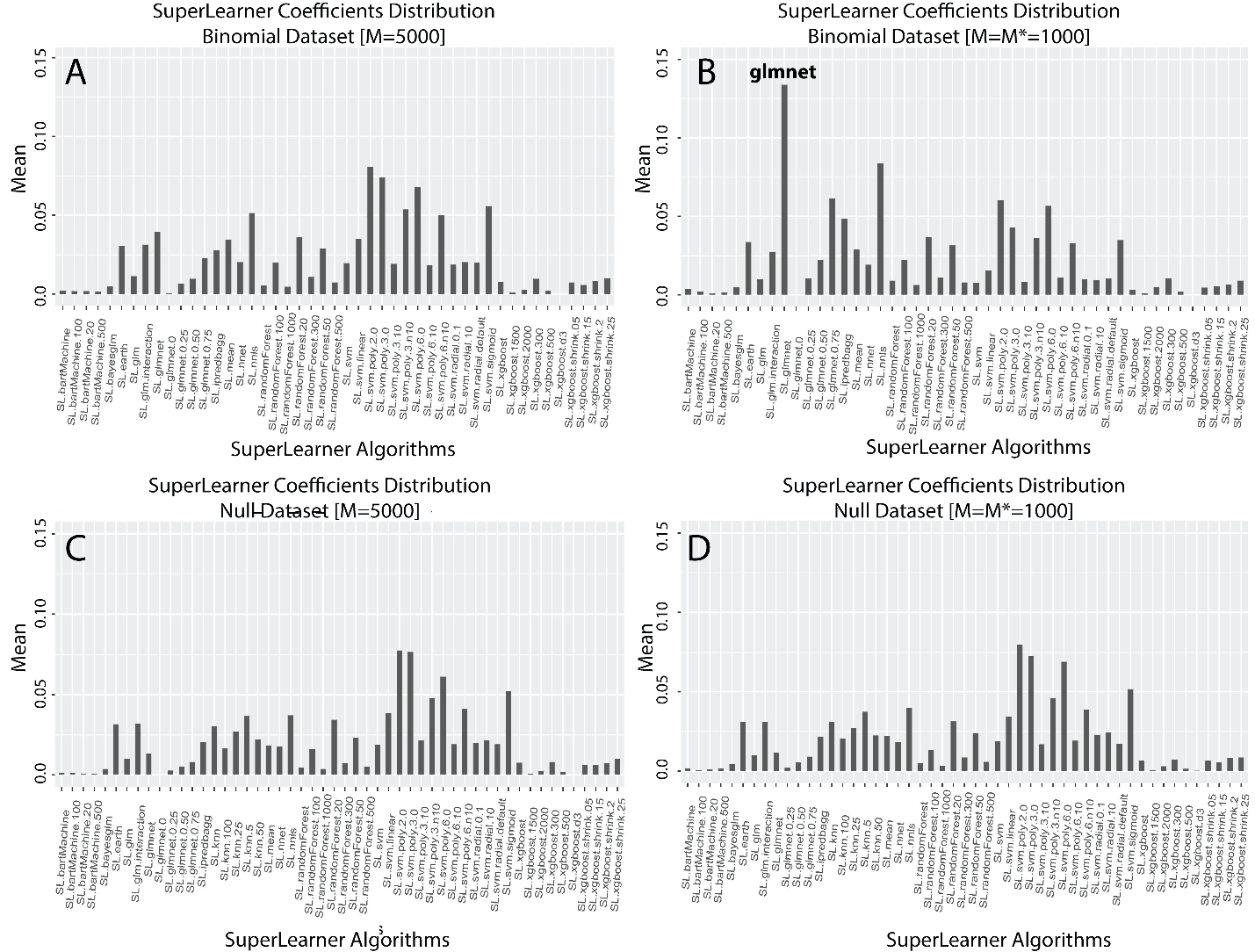


**Figure S2**: **Ensemble predictor’s coefficients/weights distribution analysis**, Binomial (**Panels A-B**) and Null (**Panels C-D**) datasets analysis (each with 10,000 cases and 1,000 features). The x axis displays the list of the algorithm in the SuperLearner library. The y axis shows the mean value of each SuperLearner coefficient across the $M=5,000$ (**Panels A** and **C**) and $M=M^{*}=1,000$ (**Panels B** and **D**) top-ranked models.

## Supplementary Figure S3

## SuperLearner coefficient distribution for the synthetic datasets with 100,000 and 1 million cases, and 10,000 features

| 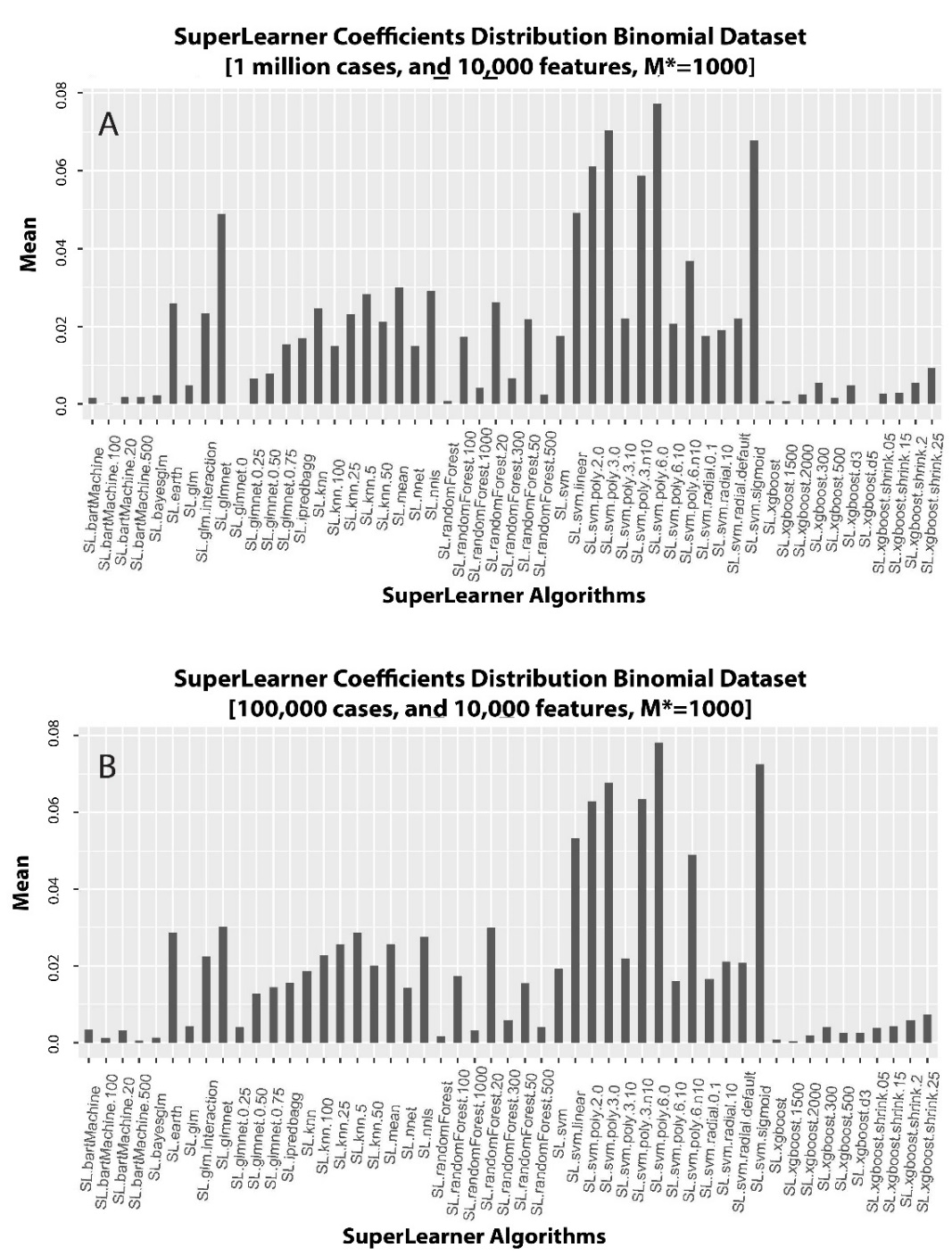 |
| --- |
| **Figure S3**: **Ensemble predictor’s coefficients/weights distribution analysis of the Binomial dataset CBDA analysis**. **Panel A**: Binomial dataset 1 million cases and 10,000 features. **Panel B**: 100,000 cases and 10,000 features. |

## Supplementary Figure S4

**Dissimilarity and variance analysis of the coefficients/weights distributions of the ensemble predictor**


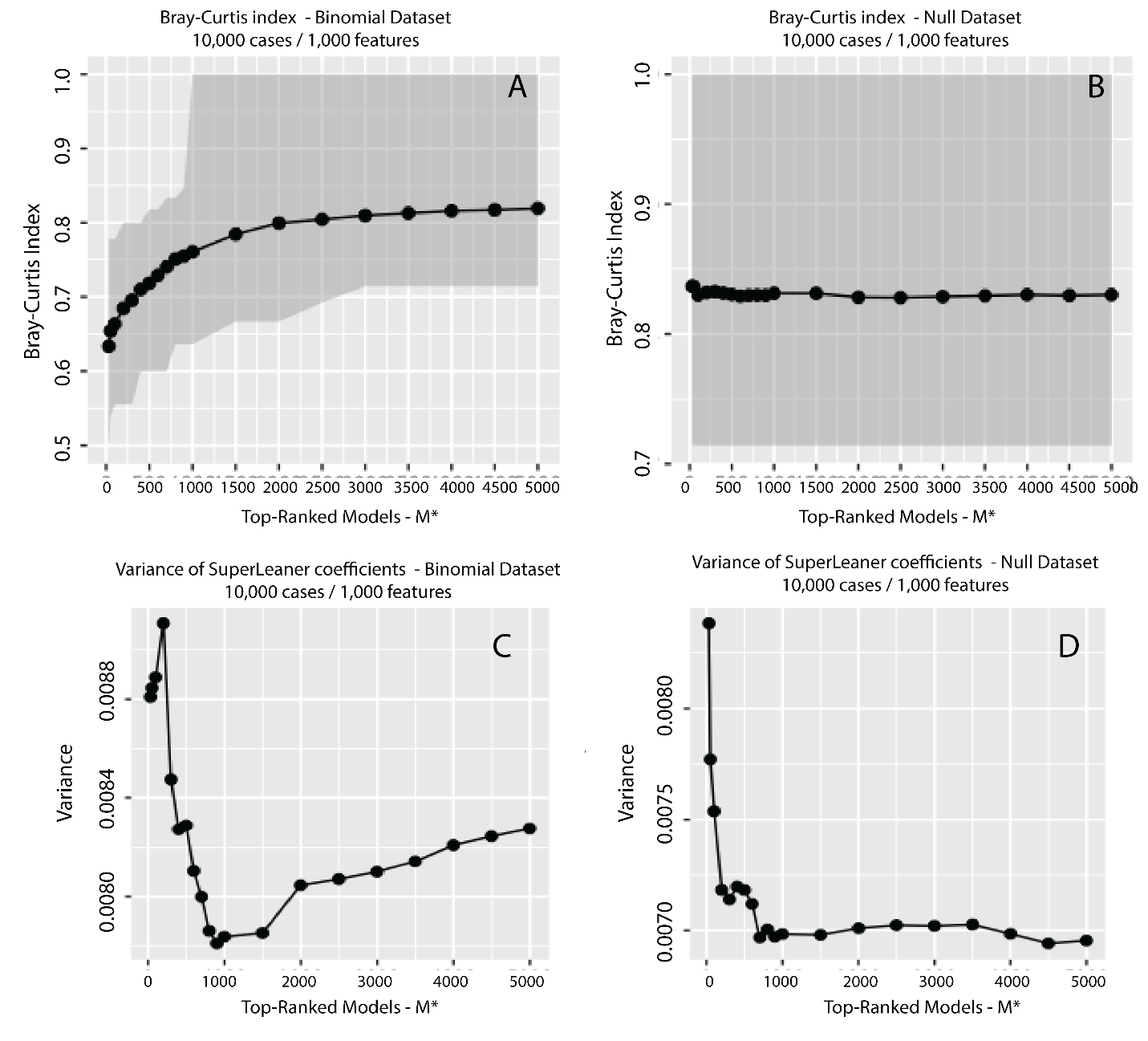


**Figure S4: Dissimilarity and variance analysis of the coefficients/weights distributions of the ensemble predictor.** Binomial (**Panels A-C**) and Null (**Panels B-D**) datasets analysis (each with 10,000 cases and 1,000 features). The x axis displays the top-ranked models $M^{*}$ (from 50 to 5,000). The y axis shows the mean value of the Bray-Curtis dissimilarity distance within the SuperLearner coefficients (**Panels A-B**) and the variance of the SuperLearner coefficients (**Panels C** and **D**).

## Supplementary Figure S5

## Overfitting plots for the UK Biobank dataset CBDA analysis with FSR=180

| 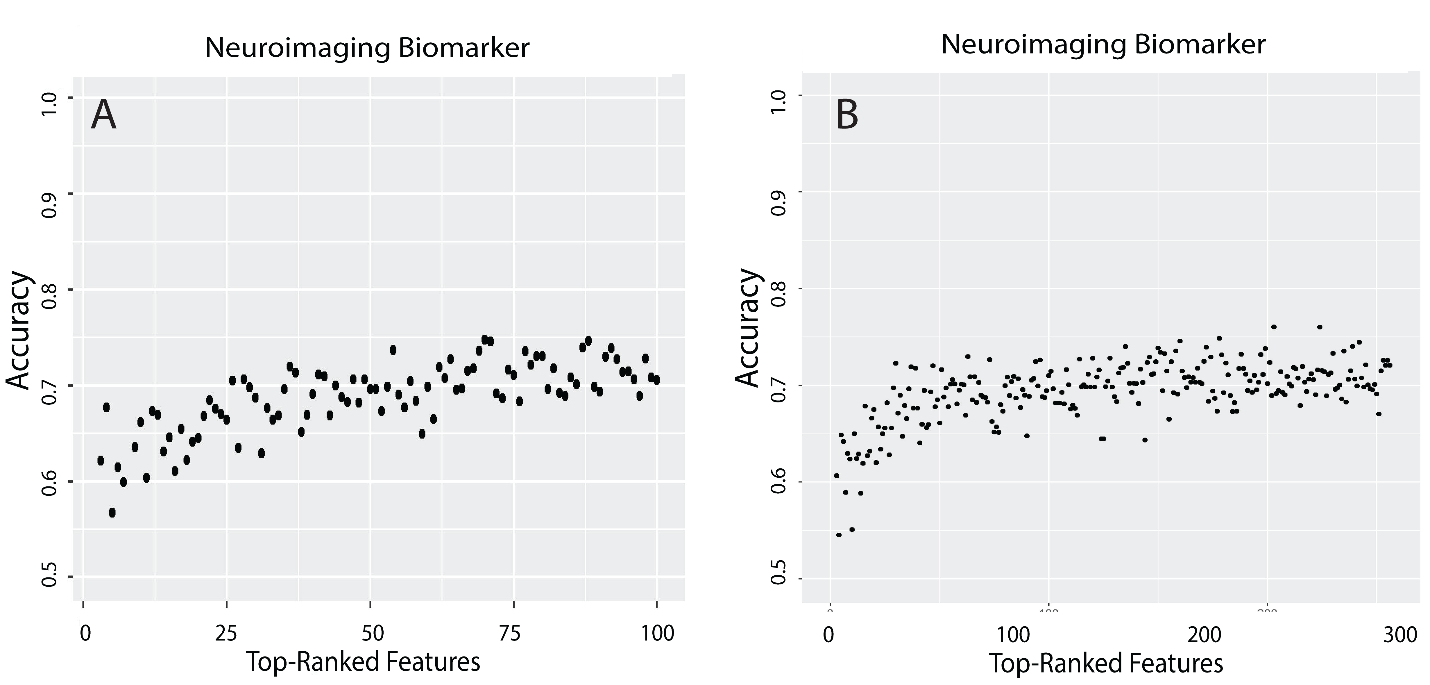 |
| --- |
| **Figure S5**: **Overfitting plots for the UK Biobank dataset CBDA analysis with FSR=180**. The x axis shows the top 300 features returned by the CBDA training stage on the UK Biobank dataset. The y axis represents the accuracy of the nested models including the top 300 features after the CBDA Overfitting Test stage. **Panel A**: subsampling with 30 features. The complete list of **Panel B**: subsampling with 180 features. |

**Supplementary Figure S6**: **CBDA training stage for the UK Biobank dataset**. SuperLearner coefficients distribution analysis (**Panels A** and **B**), **Panel C**: Bray-Curtis similarity index on the SuperLearner coefficients distribution as a function of the top-ranked models $M^{*}$. **Panel D**: variance of the SuperLearner coefficients distribution as a function of the top-ranked models $M^{*}$.


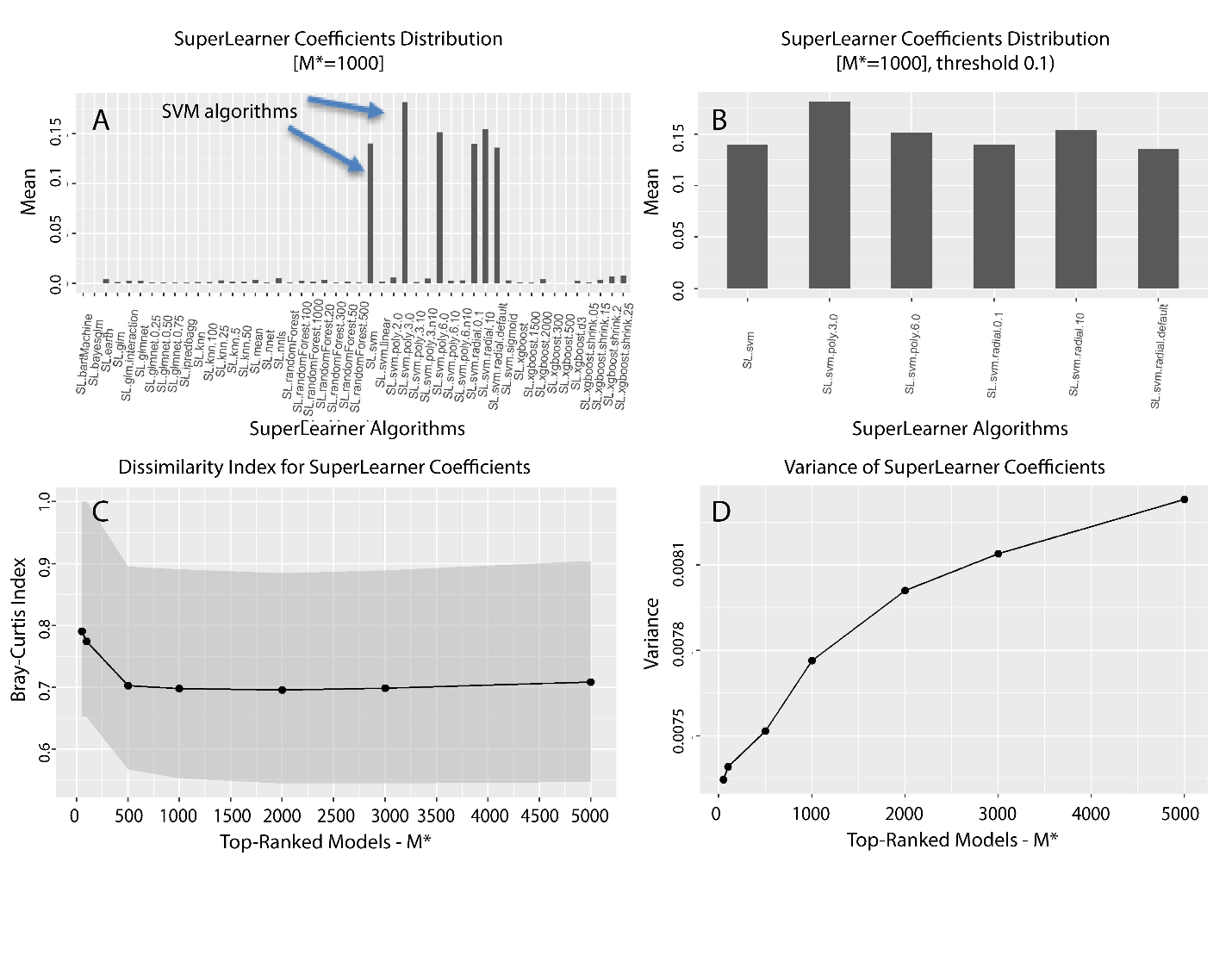

Supplement: S1 File — (DOCX) [file pone.0228520.s001.docx]
